# Pentaphosphaferrocene-mediated synthesis of asymmetric organo-phosphines starting from white phosphorus

**DOI:** 10.1038/s41467-021-26002-7

**Published:** 2021-10-01

**Authors:** Stephan Reichl, Eric Mädl, Felix Riedlberger, Martin Piesch, Gábor Balázs, Michael Seidl, Manfred Scheer

**Affiliations:** Institute of Inorganic Chemistry, University of Regensburg, Universitätsstraße 31, 93053 Regensburg, Germany

**Keywords:** Asymmetric synthesis, Ligands

## Abstract

The synthesis of phosphines is based on white phosphorus, which is usually converted to PCl_3_, to be afterwards substituted step by step in a non-atomic efficient manner. Herein, we describe an alternative efficient transition metal-mediated process to form asymmetrically substituted phosphines directly from white phosphorus (P_4_). Thereby, P_4_ is converted to [Cp*Fe(η^5^-P_5_)] (**1**) (Cp* = η^5^-C_5_(CH_3_)_5_) in which one of the phosphorus atoms is selectively functionalized to the 1,1-diorgano-substituted complex [Cp*Fe(η^4^-P_5_R′R″)] (**3**). In a subsequent step, the phosphine PR′R″R‴ (R′ ≠ R″ ≠ R‴ = alky, aryl) (**4**) is released by reacting it with a nucleophile R‴M (M = alkali metal) as racemates. The starting material **1** can be regenerated with P_4_ and can be reused in multiple reaction cycles without isolation of the intermediates, and only the phosphine is distilled off.

## Abstract (web summary)

Asymmetrically substituted phosphine ligands play a decisive role in catalysis, but their synthesis is rather challenging and often involves hazardous chemicals such as PCl_3_ or PH_3_. Here, the authors report the one-pot preparation of asymmetric phosphines from white phosphorus via a pentaphosphaferrocene based modular system, in which the transition metal complex can be reused.

## Introduction

**Fig. 1:**
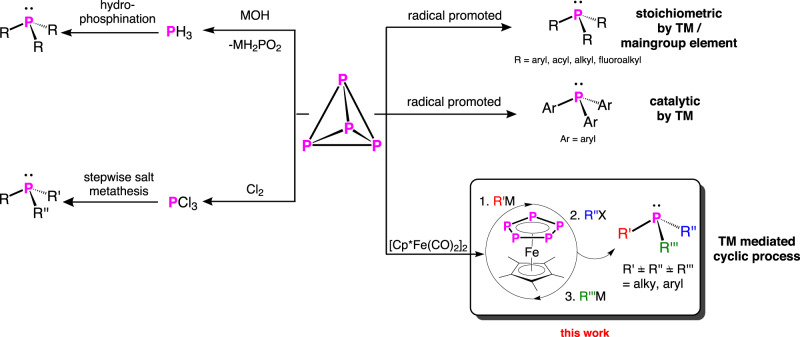
Different approaches for the synthesis of ternary phosphines. Left: conventional way, hydrophosphination and salt metathesis. Right: Radical promoted, stochiometric and catalytic, as well as the ‘semi-catalytic’ transition metal (TM) mediated synthesis of ternary phosphines.

The interest in organophosphorus compounds in life science, material science, and especially in ligand design for catalysis is an omnipresent topic^1–5^. Besides the use of phosphines as ligands^4–6^, the resulting complexes are widely used as catalysts in all areas of organic and organometallic chemistry^7^. One of the most prominent examples of a catalyst containing phosphines represents the Wilkinson’s catalyst [RhCl(PPh_3_)]^8^, which catalyses, e.g. the hydrogenation of olefins. Over the last decades, a plethora of organophosphorus compounds was synthesised and investigated^9–13^. Although organophosphorus chemistry is a well-established area, the synthesis of asymmetrically substituted organophosphorus compounds is a crucial and challenging topic^2,6,14,15^. Its importance in catalysis was awarded, e.g. with the Nobel prize^16^. The classical way to synthesise phosphines is via salt metathesis or hydrophosphination starting from PCl_3_ or PH_3_, respectively (Fig. 1)^12,13,17^. In both cases, the targeted variation of the organic substituents is synthetically very challenging. Moreover, both PCl_3_ and PH_3_ are synthesised from P_4_ either by reaction with chlorine gas or by hydrolysis in basic or acidic aqueous media^18^. Note that, aryl phosphines cannot be synthesized via hydrophosphination^19^. Interestingly, first attempts to synthesise organophosphorus compounds directly from phosphates have already been made by reduction with HSiCl_3_ followed by reaction with RCl^20^, although this development is still in its infancy^21^. Since both Cl_2_ and PH_3_ are toxic gases and because the synthesis of phosphines from PCl_3_ is associated with large (stoichiometric) amounts of side products, a more sustainable and atom-efficient process for a direct conversion of P_4_ into organophosphorus reagents is desirable. Besides these classical synthetic strategies, a few protocols have been developed for the direct synthesis of phosphines from P_4_. The first one of these protocols involves the generation of aryl radicals from aryl halides induced by an unsaturated Ti(III) complex^22^. The fundamental synthetic approach of all these protocols involves either an electrochemical approach^23^ or, more often, the generation of organo radicals from the corresponding halides induced by transition metal complexes^22,24–26^ as well as, quite recently, organotin-compounds^27^. These radical-promoted routes can be divided into stochiometric^22,24,27^ and/or (photo)catalytic approaches^25,26^.

The major drawback to all of these processes is that they do not lead to the formation of asymmetrically substituted phosphines, which are crucial for catalysis. In this report, we present a conceptually innovative strategy for a controlled and directed synthesis of symmetrically and asymmetrically substituted phosphines starting from P_4_ and carbon-centred nucleophiles and electrophiles by using [Cp*Fe(η^5^-P_5_)] (**1**) (Cp* = η^5^-C_5_(CH_3_)_5_) as a P-atom carrier.

## Results

### Synthesis and structural characterization of the mono-substituted complexes 2c-e

**Fig. 2:**
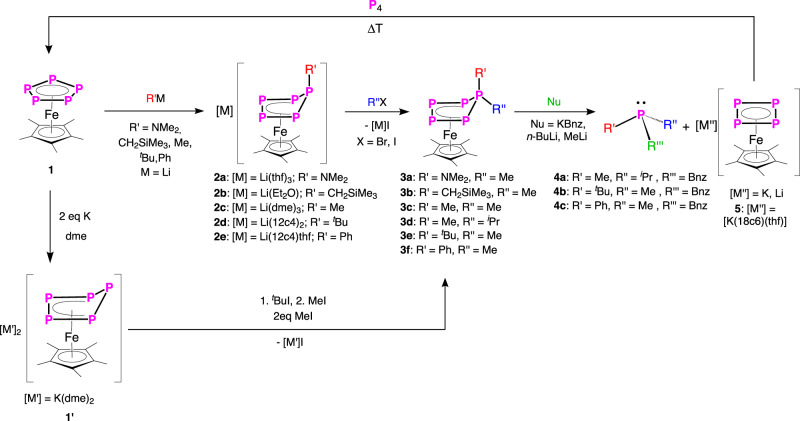
Synthesis of asymmetric phosphines 4, via successive nucleophilic, electrophilic, nucleophilic attack. Synthesis of the anionic precursor complexes **2** by nucleophilic attack; electrophilic quenching of **2** (synthesis of **3**); asymmetric phosphine abstraction by nucleophiles (synthesis of **4**, **5**); regeneration of **1** by thermolysis with P_4_; alternative synthesis of **3** by electrophilic quenching of **1′**.

In our previous works, we were able to show that [Cp*Fe(η^5^-P_5_)] (**1**) readily reacts with main-group nucleophiles such as Me_3_SiCH_2_^−^ or Me_2_N^−^ via the formation of a P–C/P–N bond^28^, leading to the complexes [Cp*Fe(η^4^-P_5_R′)]^−^ (R′ = CH_2_SiMe_3_ (**2a**), NMe_2_ (**2b**)). However, we found now that the nucleophile used can be freely varied, and basically any alkali metal organyl can be used (Fig. 2). The reaction of [Cp*Fe(η^5^-P_5_)] (**1**) with MeLi, ^*t*^BuLi and PhLi, respectively, at −80 °C leads to an immediate colour change from green to brown. After workup, the complexes [Li(dme)_3_][Cp*Fe(η^4^-P_5_Me)] (**2c**), [Li(12c4)_2_][Cp*Fe(η^4^-P_5_^*t*^Bu)] (**2d**), [Li(12c4)(thf)][Cp*Fe(η^4^-P_5_Ph)] (**2e**) can be isolated in crystalline yields of 84%, 86% and 75%, respectively.

**Fig. 3:**
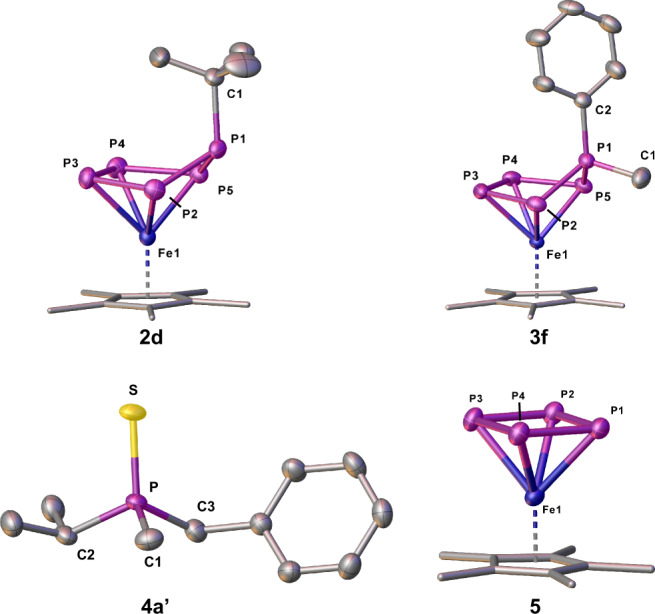
Molecular structures of the anions in 2d, 5 and of the neutral complexes 3f, 4a′ with thermal ellipsoids at 50% probability level. Cations and hydrogen atoms are omitted for clarity. The Cp* ligands are drawn in the wire frame model.

The single-crystal X-ray structure analysis of **2** shows anionic complexes with a folded P_5_R′-ligand in an envelope conformation (Fig. 3, Supplementary Figs. 40–43). The four coordinating phosphorus atoms of the P_5_R′ ligand build a nearly square planar P_4_ unit which coordinates symmetrically to the Cp*Fe fragment. The P1 atom to which the organic group is attached (P1–C1 bond length of 1.849(2) Å (**2c**), 1.898(3) Å (**2d**) and 1.841(3) Å (**2e**)) deviates from this plane. All P2–P5 bond lengths (1.993(13)–2.1575(10) Å)) are in the range between a single and a double bond^29,30^.

### Reactivity of 2 towards electrophiles

**Fig. 4:**
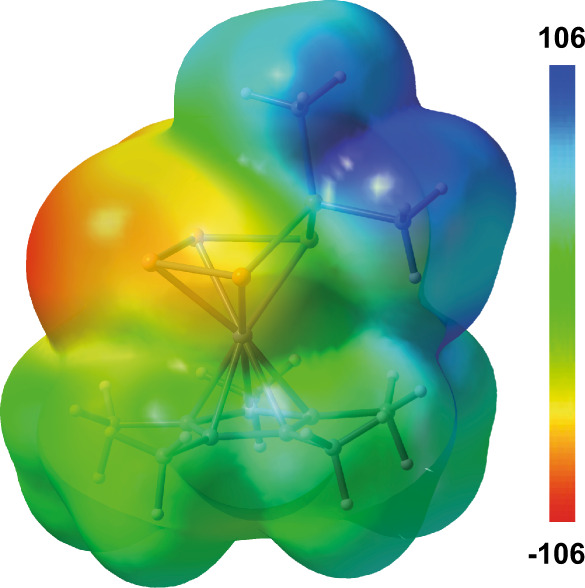
Electrostatic potential mapped on electron density (isovalue = 0.001) for 3c. Colour code (blue = positive, red = negative) in kJ·mol^−1^.

The presence of the negative charge renders the compounds **2** nucleophilic, which is why they can be quenched with electrophiles such as alkyl or aryl halides. Thus, the compounds **2** were treated with alkyl halides R″X (R″ = Me, ^*i*^Pr; X = Br, I) as common carbon-centred electrophiles. Indeed, the neutral organo-substituted polyphosphorus complexes [Cp*Fe(η^4^-P_5_R′R″)] (**3**; Fig. 2) were obtained in almost quantitative yields according to ^31^P NMR spectroscopy. The isolated compounds **2** and **3** were characterized by NMR spectroscopy (Supplementary Figs. 1–9; 17–26), mass spectrometry, elemental analysis, and single-crystal XRD (Fig. 3, Supplementary Figs. 40–50). The latter shows that, in **3**, the folded η^4^-P_5_ unit remains intact and the introduced electrophile is attached to the phosphorus atom, which bears the first organo-substituent (former nucleophiles; R′, Fig. 3), leading to a 1,1-substitution pattern with a P1–C2 bond length (1.785(5)–1.922(11) Å) corresponding to single bonds^29^. The functionalized phosphorus atoms P1 carry two organic substituents and possess a phosphonium ion-like character (Fig. 4). Of particular interest are the compounds **3a,b** and **3f** since they represent precursors for quite rare asymmetrically substituted organo-phosphine derivatives^2,31^. Due to the large diversity of alkali metal organyls and electrophilic organo halides, the shown procedure gives access to the synthesis of a plethora of complexes of type **3**. An alternative way for the synthesis of **3** is the reaction of the doubly reduced derivative of **1**, [K(dme)_2_]_2_[Cp*Fe(η^4^-P_5_)] (**1**′)^32^, with alkyl halides. This was demonstrated by the synthesis of **3c** and **3e**, which proceeds according to ^31^P NMR in a quantitative manner (Supplementary Figs. 69, S70) and shows that possible limitations of organolithium reagents needed in the former procedure can be avoided. The reaction of **1**′ with two equivalents of MeI leads smoothly to **3c**, while the addition of one equivalent of ^*t*^BuI followed by the addition of one equivalent of MeI leads to the formation of **3e** (Fig. 2). In the ^31^P NMR spectra of **2** (Supplementary Figs. 17–20) and **3** (Supplementary Figs. 21–26), AMM′XX′ spin systems were detected from which the coupling constants were obtained by simulations (Supplementary Tables 1–10). The related signals of the phosphorus atoms P1 of **3** are downfield shifted compared to **2**. This is in agreement with the phosphonium-like nature of the phosphorus atom P1 in **3**.

### Synthesis of (a)symmetric phosphines—reactivity of 3 towards nucleophiles

The electrostatic potential surface of compound **3c** (B3LYP/def2-TZVPP level of theory; Fig. 4) shows a rather localized positive potential on the phosphorus atom bearing the organo-substituents (P1). Therefore, the attack of a nucleophile is expected to occur at the position P1. Indeed, the reaction of **3d** with benzyl potassium lead to the formation of the asymmetrically substituted phosphine PMe^*i*^PrBnz (**4a**) in 82% yield (Supplementary Fig. 61) and the anion [Cp*Fe(η^4^-P_4_)]^−^ (Supplementary Fig. 55). This strategy can be extended for the synthesis of a series of asymmetric phosphines, with PR′R″R‴ (**4a-c** (**a**: R′ = Me, R″ = ^*i*^Pr, R‴ = Bnz; **b**: R′ = ^*t*^Bu, R″ = Me, R‴ = Bnz; **c**: R′ = Ph, R″ = Me, R‴ = Bnz)) being isolated as air-sensitive viscous liquids in 82% (**4a**), 55% (**4b**), 80% (**4c**) yields, respectively. The identity of the phosphine was proven by NMR spectroscopy and, after oxidation with sulfur to the corresponding phosphine sulfides (Supplementary Figs. 10–16; 27–32; 34–39), also by single-crystal X-ray diffraction analysis (Fig. 3, Supplementary Figs. 51–54). Furthermore, by changing the nucleophile to MeLi and ^*n*^BuLi, the corresponding phosphines PMe_3_ and PMe_2_^*n*^Bu can be obtained in the reaction with **3c** (Supplementary Figs. 56, 57). The latter is less selective, PMe_3_, however, can be synthesized via this route in 84% yield (according to NMR).

**Fig. 5:**
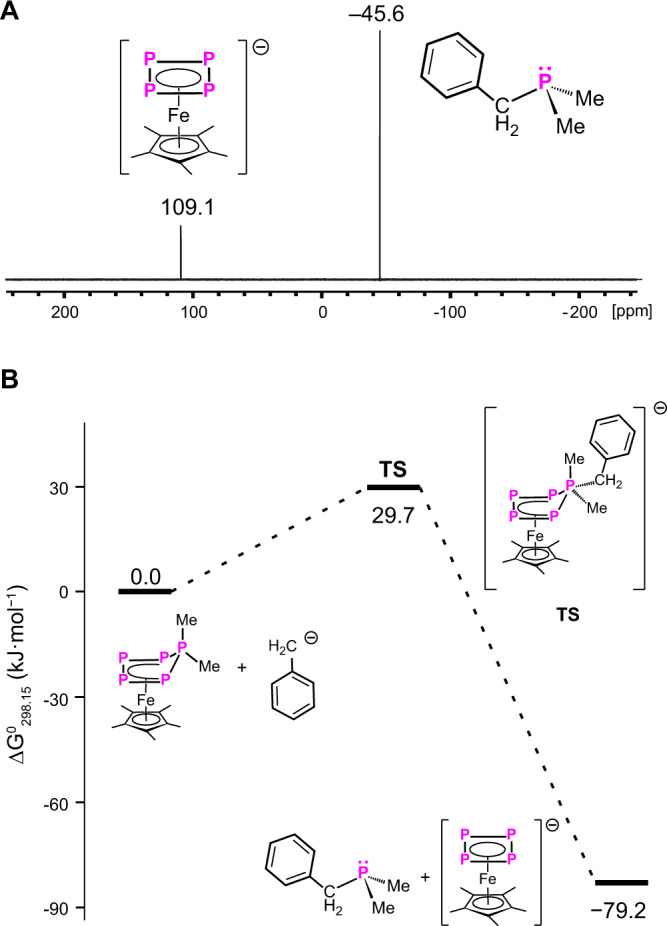
Identification of the reaction pathway of phosphine abstraction. **A** Experimental ^31^P{^1^H} NMR (242.90 MHz, THF-d_8_) spectrum of the crude reaction solution (low-temperature reaction) of **3c** with KBnz at 180 K. **B** Gibbs free energy profile of the reaction of **3c** with KBnz, calculated at the B3LYP-D3(BJ)/def2-TZVPP (PCM = THF) level of theory.

To identify potential intermediates in the reaction of **3** with nucleophiles, the reaction of **3c** with MeLi at −80 °C in THF-d_8_ was monitored by ^31^P{^1^H} NMR spectroscopy at this temperature. The ^31^P NMR spectrum clearly shows the full conversion of **3** and quantitative formation of **4** and **5** (Fig. 5A, Supplementary Fig. 68). These very fast reactions agree with the results of the DFT calculations which show a very low activation barrier for this process (Figs. 5B, S71).

The other product of the reaction of **3** with nucleophiles, i.e. [M][Cp*Fe(η^4^-P_4_)] (M = K(dme)_3_; **5**) is a rare representative of the anionic complexes with a *cyclo*-P_4_ ligand as an end-deck^33–35^. The addition of 18-crown-6 considerably increases its stability and **5** can be isolated in 81% crystalline yield, which is a major improvement over the previously reported complex [K(18c6)][Cp^Ar^Fe(η^4^-P_4_)]^−^ (Cp^Ar^ = C_5_(C_6_H_4_-4-Et)_5_) obtained in 4% yield^33^. In the solid state, the anion [Cp*Fe(η^4^-P_4_)]^−^ (Fig. 3, Supplementary Fig. 55) features a square planar *cyclo*-P_4_ ligand with bond lengths between 2.1666(13) and 2.175(13) Å, which are in the range between a single^29^ and a double bond^30^. In the ^31^P{^1^H} NMR spectrum of **5**, a singlet is observed at 118.9 ppm (Supplementary Fig. 39). The lithium derivative of **5** can be detected in all reactions of **3b-e** with organolithium reagents but decomposes partly over time in solution.

### Formation of phosphines and regeneration of the carrier platform [Cp*Fe(η^5^-P_5_)] (1) in a ‘semi-catalytic’ cyclic process

**Fig. 6:**
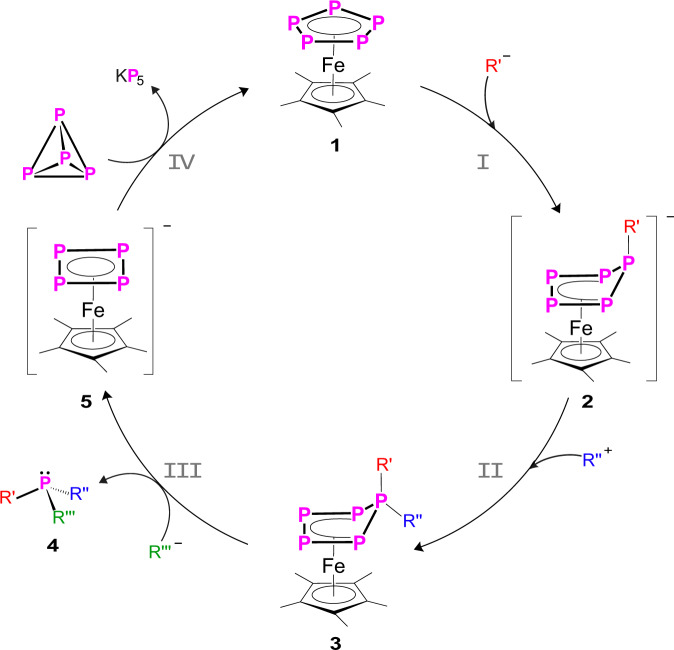
‘Semi-catalytic’ cycle for the synthesis of asymmetric phosphines. One-pot reaction of [Cp*Fe(η^5^-P_5_)] (**1**) with MeLi (I, R′ = Me), quenching with MeI (II, R″ = Me), reaction with KBnz (III, R‴ = Bnz) and subsequent thermolysis with white phosphorus (IV).

The phosphines generated in the reaction of **3** with nucleophiles can be easily isolated either by extraction with *n*-hexane (**5** is not soluble in *n*-hexane) or, in the case of volatile phosphines, by condensation. That is why we were interested in designing a closed-cycle-process for the generation of phosphines in which **1** represents the platform for the phosphorus atom transfer. To close the circle, **5** was reacted with one equivalent of P_4_ with **1** being formed in almost quantitative yield, among KP_5_, which partly decomposes to polyphosphides^36^ (Supplementary Fig. 65). After workup, **1** can be isolated in 81% crystalline yield (Fig. 6). If required, KP_5_ can be transformed into the corresponding pentaphosphaferrocene by reacting with [Cp*FeBr]_2_^37^ to increase the atom efficiency of this process. Moreover, it is possible to perform a larger scale synthesis of the phosphines in a one-pot reaction to easily reuse **1** and to avoid the workup of the reaction solution. In a one-pot reaction, compound **1** was dissolved in 2,5,8,11,14-pentaoxapentadecane (tetraglyme) and stoichiometric amounts of MeLi and MeI were successively added at r.t. with 5 min stirring in between (Fig. 6, steps I and II). Subsequently, the solution was cooled to −30 °C and one equivalent of KBnz in tetraglyme was added (Fig. 6, step III). The cooling bath was removed, the formed phosphine PMe_2_Bnz was distilled off under reduced pressure (1 × 10^−3^ mbar, 55 °C) and was obtained in 87% yield (Fig. 6, step III, Supplementary Fig. 67). To the remaining solution, one equivalent of white phosphorus was added and heated under reflux for 1 h at 275 °C (IV). The ^31^P{^1^H} NMR spectrum (Supplementary Fig. 63) shows the almost quantitative regeneration of **1**. The same protocol was repeated two more times on the same reaction solution (see Supplementary Fig. 64). The phosphine PMe_2_Bnz was isolated in 82–67% yield (overall isolated yield 79%, see Supplementary Fig. 67, Supplementary Table 40). After these three cycles, **1** can be isolated from the reaction solution in 69% yield (Supplementary Fig. 66), indicating that this process could be carried on for many more cycles.

In summary, the polyphosphorus compound [Cp*Fe(η^5^-P_5_)] (**1**) can be used as a recyclable platform for the targeted synthesis of symmetric or asymmetric phosphines, via a sequence of nucleophilic-electrophilic-nucleophilic reactions, directly from white phosphorus, avoiding the use of intermediate products such as PCl_3_ or PH_3_. With this modular system, asymmetric phosphines are obtained in high yields in significant preparative scales and **1** can be regenerated and reused in a ‘semi-catalytic’ cyclic process, which can be run for several cycles in a one-pot reaction. The presented results pave the way for a selective and easy synthetic route to asymmetric and (in future work potential) chiral phosphines based on white phosphorus. This conceptual innovative approach avoids radicals and is not limited to aryl or alkyl substituents, but phosphines with a variety of different substitution patterns are now accessible in high yields by a very simple approach.

## Methods

### General methods

All manipulations were carried out under an inert atmosphere of dried argon using standard Schlenk and glove box techniques. 1,2-dimethoxyethane (DME), 2,5,8,11-tetraoxadodecane (triglyme) and 2,5,8,11,14-pentaoxapentadecane (tetraglyme) were dried and deoxygenated by distillation under argon atmosphere from sodium (DME) or calcium hydride. All other solvents were dried using a MB SPS-800 device of the company MBRAUN and stored over molecular sieve. NMR spectra were recorded on a Bruker Avance III 400/600 MHz NMR spectrometer. Chemical shifts were measured at ambient temperature and are given in ppm; they are referenced to TMS for ^1^H and 85% H_3_PO_4_ for ^31^P as external standard. Signal multiplicities are described using common abbreviations: s (singlet), d (doublet), t (triplet), q (quartet), quint (quintet), m (multiplet) and br (broad). LIFDI-/FD-/EI-MS spectra (LIFDI = liquid injection field desorption ionization, FD = field desorption, EI = electron ionization) were measured on a JEOL AccuTOF GCX. ESI-MS spectra (ESI = electrospray ionization) were measured on an Agilent Q-TOF 6540 UHD. Elemental analysis (CHN) was determined using a Vario micro cube instrument. A glass stirring bar was used in reactions with potassium benzyl. DFT calculations were carried out using the Gaussian 16 program^38^. The geometries were optimised using the B3LYP^39–42^ functional together with the def2-TZVPP basis set^43^. Solvation effects were incorporated via the polarizable continuum model^44^ using the dielectric constant of THF. Dispersion effects were incorporated via Grimme’s correction with Becke-Johnson damping^45^.

### Unless otherwise stated, all other chemicals were obtained from commercial sources

Crystals suitable for single-crystal X-ray diffraction analysis were obtained as described in the corresponding synthetic protocols below. The diffraction data were collected either on a Gemini Ultra diffractometer equipped with an Atlas^S2^ CCD detector and with a fine-focus sealed Cu-K_α_ X-ray tube, on a XtaLAB Synergy R, DW system diffractometer equipped with a HyPix-Arc 150 detector and a rotating-anode Cu-K_α_ X-ray tube or a GV50 diffractometer equipped with a Titan^S2^ CCD detector and a micro-focus Cu-K_α_ X-ray tube. Data collection and reduction were performed with CrysAlisPro software package. The structures were solved with Olex2^46^, using ShelXT^47^ and a least‐square refinement on *F*2 was carried out with ShelXL^48^. All non‐hydrogen atoms were refined anisotropically. Hydrogen atoms at the carbon atoms were located in idealized positions and refined with isotropic displacement parameters according to the riding model.

The images of the molecular structures were made using Olex2^46^.

### Synthetic protocols

#### Synthesis of [Cp*Fe(η^5^-P_5_)] (**1**)^49^

The complex [Cp*Fe(CO)_2_]_2_ (10 g, 20.0 mmol) and white phosphorus (7 g, 56.5 mmol) are dissolved in 1,3-diisopropylbenzene (500 ml) and heated to reflux for 7 h. The solvent was removed in vacuo. The residue was dissolved in dichloromethane, silica was added and the solvent was removed under reduced pressure. The preabsorbed reaction mixture was purified via column chromatography (SiO_2_, hexane, 10 × 3 cm). Using *n*-hexane, compound **1** (12.3 g, 35.6 mmol, 89%) can be eluted as a dark green fraction. Complex **1** can be obtained after 1 day from a concentrated solution stored at −30 °C as green needles.

#### Synthesis of [K(dme)_2_]_2_[Cp*Fe(η^4^-P_5_)] (**1′**)^32^

A solution of **1** (0.4 g, 1.16 mmol, 1 eq) in DME (30 ml) was added to potassium (0.136 g, 3.47 mmol, 3 eq). The solution was stirred at ambient temperature for 4 h. The solution was filtered; the product was extracted from an insoluble residue with DME (70 ml). The volume of the combined solution was reduced to ca. 30 ml and Et_2_O (30 ml) was added. The suspension was stirred overnight and the product was collected on a frit, washed with Et_2_O (30 ml) and dried in vacuum to give **1′** (0.74 g, 0.95 mmol) as dark brown-green powder.

#### Synthesis of [Li(thf)_3_][Cp*Fe(η^4^-P_5_NMe_2_)] (**2a**)^28^

To a solution of **1** (176 mg, 0.51 mmol) in 5 mL THF a solution of LiNMe_2_ (26 mg, 0.51 mmol) was added at −35 °C. The colour of the solution turned immediately from green to brown. The reaction mixture was stirred over 20 h, the solvent was concentrated to 3 mL and finally layered with *n*-hexane. After 4 days on room temperature light brown square-shaped crystals of **2a** (150 mg, 0.25 mmol, 50%) were formed.

#### Synthesis of [Li(dme)_3_][Cp*Fe(η^4^-P_5_Me)] (**2b**)^49^

To a solution of ((trimethylsilyl)methyl)lithium (68 mg, 0.72 mmol) in Et_2_O (5 mL) at −35 °C was added a solution of **1** (250 mg, 0.72 mmol) in Et_2_O (15 mL). Immediate colour change was observed. The solution was slowly warmed up to ambient temperature and volume of the reaction mixture was reduced to ca. 15 mL and layered with *n*-hexane (20 mL). Black crystals of **2b** (342 mg, 0.66 mmol, 92%) were formed after 2 days at 0 °C.

#### Synthesis of [Li(dme)_3_][Cp*Fe(η^4^-P_5_Me)] (**2c**)

To a solution of 138.0 mg (0.4 mmol, 1 eq) of **1** in DME at −60 °C, a 1.6 molar solution of MeLi (0.4 mmol, 0.27 mL, 1 eq) in Et_2_O (diethylether) was added and an immediate colour change to brown could be observed. The solution was stirred for 4 h, the solvent was reduced to 5 mL and layered with *n*-hexane (25 mL). [Li(dme)_3_][Cp*Fe(η^4^-P_5_Me)] (**2c**) was isolated as black truncated prisms. Yield: 216.0 mg (0.34 mmol, 83%). ^1^H NMR (thf-d_8_, 293 K): *δ* [ppm] = 1.49 (s, 15H, C_5_(CH_3_)_5_), 0.39 (m, 3H, -(CH_3_)). ^31^P{^1^H} NMR (thf-d_8_, 293 K): *δ* [ppm] = 71.3 (m, 1P, P_A_), 13.7 (m, 2P, P_M,M′_), −71.5 (m, 2P, P_x,x′_). ^31^P NMR (thf-d_8_, 293 K): *δ* [ppm] = 71.3 (m, 1P, P_A_), 13.7 (m, 2P, P_M,M′_), −71.5 (m, 2P, P_x,x′_); For coupling constants, see Supplementary Tables 1–10. ESI-MS (anion, DME): *m/z* = 360.95 (6%, [M]^−^), 376.94 (8%, [MO]^−^), 392.93 (100%, [MO_2_]^−^); analysis (calcd., found for C_23_H_48_FeLiO_6_P_5_): C (43.28, 42.84), H (7.58, 7.42).

#### Synthesis of [Li(12c4)_2_][Cp*Fe(η^4^-P_5_^*t*^Bu)] (**2d**)

Compound **1** (0.2 mmol, 69.2 mg, 1 eq) was dissolved in 10 mL THF (tetrahydrofuran) and cooled to –80 °C. A 1.7 molar solution of ^*t*^BuLi (0.2 mmol, 0.12 mL, 1 eq) in *n*-pentane was added dropwise. An immediate change in colour from green to brown occurred. The mixture was allowed to stir overnight and reach room temperature. 12-crown-4 (12c4) (0.4 mmol, 0.07 mL, 2 eq) was added to the brown solution and stirred for 30 min at room temperature. The solvent was removed under reduced pressure. The brown residue was washed with 2 × 10 mL *n*-hexane, dissolved in 2 mL THF and layered with 5 mL toluene. The mixture was stored at 8 °C. Compound **2c** was isolated after 1 day as dark green plates. Yield: 110.2 mg (0.17 mmol, 86%). ^1^H NMR (THF-d_8_, 293 K): *δ* [ppm] = 3.78 (s, 33H, 12c4), 1.45 (s, 15H, C_5_(CH_3_)_5_), 0.03 (d, 9H, ^3^*J*_P-H_ = 10.8 Hz, -C(CH_3_)_3_). ^31^P{^1^H} NMR (THF-d_8_, 293 K): *δ* [ppm] = 105.6 (m, 1P, P_A_), 19.6 (m, 2P, P_M,M′_), − 70.2 (m, 2P, P_x,x′_). ^31^P NMR (THF-d_8_, 293 K): *δ* [ppm] = 105.6 (m, 1P, P_A_), 19.6 (m, 2P, P_M,M′_), −70.2 (m, 2P, P_x,x′_); (for coupling constants, see Supplementary Tables 2, 3). ESI-MS (anion, DME): *m/z* = 403.00 (100%, [M]^−^); analysis (calcd., found for C_30_H_56_FeLiO_8_P_5_): C (47.26, 47.61), H (7.40, 7.49).

#### Synthesis of [Li(12c4)(thf)][Cp*Fe(η^4^-P_5_Ph)] (**2e**)

Compound **1** (0.2 mmol, 69.2 mg, 1 eq) was dissolved in THF, cooled to −80 °C and a 1.9 molar solution of PhLi in dibutyl ether (0.2 mmol, 0.11 mL, 1 eq) was added. A colour change from green to brown occurred. Two equivalents of 12-crown-4 (0.4 mmol, 0.12 mL) were added and the solution was stirred overnight and allowed to reach room temperature. The solvent was removed under reduced pressure. The resulting brownish-green residue was washed with 10 mL of *n*-hexane and again dissolved with 3 mL of THF layered with 10 mL of *n*-hexane and stored at room temperature. Dark green blocks of **2e** were isolated after 1 week. Yield: 102.0 mg (0.15 mmol, 75%). ^1^H NMR (THF-d_8_, 293 K): *δ* [ppm] = 6.56 (m, 5H, -C_6_H_5_), 3.72 (s, 17H, 12c4), 3.60 (m, 3H, THF-H_8_), 1.77 (m, 3H, THF-H_8_), 1.51 (s, 15H, C_5_(CH_3_)_5_). ^31^P{^1^H} NMR (C_6_D_6_, 293 K): *δ* [ppm] = 79.3 (m, 1P, P_A_), 21.1 (m, 2P, P_M,M′_), −61.84 (m, 2P, P_x,x′_). ^31^P NMR (C_6_D_6_, 293 K): *δ* [ppm] = 79.3 (m, 1P, P_A_), 21.1 (m, 2P, P_M,M′_), −61.84 (m, 2P, P_x,x′_); (For coupling constants, see Supplementary Table 4). ESI-MS (anion, DME): no signal can be assigned; analysis (calcd., found for C_28_H_44_FeP_5_LiO_5_): C (49.58, 49.80), H (6.54, 6.53).

#### Synthesis of [Cp*Fe{η^4^-P_5_(NMe_2_)Me}] (**3a**)

Compound **2a** (0.3 mmol, 168.0 mg, 1 eq) was dissolved in 50 mL THF. A 1.11 molar solution of MeI in diethylether (0.3 mmol, 0.27 mL, 1 eq) was added. The mixture was allowed to stir overnight. The solvent was removed under reduced pressure and a brown solution was extracted with 20 mL *n*-hexane and filtered over diatomaceous earth. The solvent was reduced to 5 mL and stored at −30 °C. **3a** was formed as dark prisms after 1 day. Yield: 69.1 mg (0.17 mmol, 57%). ^1^H NMR (C_6_D_6_, 293 K): *δ* [ppm] = 1.86 (dt, 3H, ^2^*J*_P-H_ = 11.7 Hz, ^3^*J*_P-H_ = 5.8 Hz, -(CH_3_), 1.53 (d, ^3^*J*_P-H_ = 10.4 Hz, 6H, N(CH_3_)_2_). ^31^P{^1^H} NMR (C_6_D_6_, 293 K): *δ* [ppm] = 130.8 (m, 1P, P_A_), −65.1 (m, 2P, P_x,x′_), 37.7 (m, 2P, P_M,M′_). ^31^P NMR (C_6_D_6_, 293 K): *δ* [ppm] = 130.8 (m, 1P, P_A_), −65.1 (m, 2P, P_x,x′_), 37.7 (m, 2P, P_M,M′_) (for coupling constants, see Supplementary Table 4). LIFDI-MS (toluene): 405.02 (100%, [M]^+^); analysis (calcd., found for C_13_H_24_FeP_5_N): C (38.55, 38.14), H (5.97, 6.03).

#### Synthesis of [Cp*Fe{η^4^-P_5_(CH_2_SiMe_3_)Me}] (**3b**)

Compound **2b** (1.33 mmol, 686.0 mg, 1 eq) was dissolved in 100 mL THF. 1.2 mL of a 1.110 molar solution of MeI in Et_2_O (1.33 mmol, 1 eq) was added under strong stirring at room temperature and the solution was stirred overnight. The formation of a white precipitate was observed on the glass wall. The solvent was removed, **3b** extracted with 40 mL of *n*-hexane and filtered over diatomaceous earth. The solvent was reduced to 10 mL. Complex **3b** was isolated as black plates. Crystals for X-ray structure analysis were obtained from a concentrated *n*-hexane solution at −30 °C. Yield: 426 mg (0.94 mmol, 71%). ^1^H NMR (C_6_D_6_, 293 K): *δ* [ppm] = 1.80 (dt, 3H, ^2^*J*_P-H_ = 11.9 Hz, ^3^*J*_P-H_ = 5.8 Hz, -CH_3_), 1.67 (s, 15H, C_5_(CH_3_)_5_), 0.25 (d, 2H, ^2^*J*_P-H_ = 14.9 Hz, CH_2_Si(CH_3_)_3_), −0.18 (s, 9H, CH_2_Si(CH_3_)_3_). ^31^P{^1^H} NMR (C_6_D_6_, 293 K): *δ* [ppm] = 129.4 (m, 1P, P_A_), 37.0 (m, 2P, P_M,M′_), −96.7 (m, 2P, P_x,x′_). ^31^P NMR (C_6_D_6_, 293 K): *δ* [ppm] = 129.4 (m, 1P, P_A_), 37.0 (m, 2P, P_M,M′_), −96.7 (m, 2P, P_x,x′_); (for coupling constants, see Supplementary Table 5). LIFDI-MS (toluene): 448.03 (100%, [M]^+^); analysis (calcd., found for C_15_H_29_FeP_5_Si): C (40.20, 39.64), H (6.52, 6.48).

#### Synthesis of [Cp*Fe(η^4^-P_5_Me_2_)] (**3c**)

Method A: Compound **2c** (3.53 mmol, 2.26 g, 1 eq) was dissolved in DME and 3.18 mL of a 1.11 molar solution of MeI (3.53 mmol, 1 eq) in Et_2_O was added at room temperature and stirred overnight. The solvent was removed in vacuo, a brown residue was extracted with *n*-hexane and filtered over silica gel. The solvent was removed under reduced pressure, dissolved in 5 mL of CH_2_Cl_2_, layered with 20 mL of acetonitrile and stored at −30 °C. Dark green blocks of **3c** were isolated after 1 week. Yield: 938 mg (2.49 mmol, 71%).

Method B: [K(dme)_2_]_2_[Cp*Fe(η^4^-P_5_)] (**1**′) (0.2 mmol, 165.9 mg, 1 eq) was dissolved in 10 mL THF and 0.36 mL of a 1.11 molar solution of MeI (0.4 mmol, 2 eq) in Et_2_O was added at room temperature. The reaction mixture was stirred for five min and the solvent was partly reduced in vacuo. The performed ^31^P{^1^H} NMR experiment (Supplementary Fig. 69) of the reaction in THF/C_6_D_6_-capillary matches the spectroscopic data of method A and shows a quantitative formation of **3c**. ^1^H NMR (C_6_D_6_, 293 K): *δ* [ppm] = 1.61 (s, 15H, C_5_(CH_3_)_5_), 1.51 (dt, 3H, ^2^*J*_P-H_ = 11.1 Hz, ^3^*J*_P-H_ = 5.5 Hz, -CH_3_(exo)), 0.06 (d, 3H, ^2^*J*_P-H_ = 11.5 Hz, -CH_3_(endo)). ^31^P{^1^H} NMR (C_6_D_6_, 293 K): *δ* [ppm] = 117.2 (m, 1P, P_A_), 35.4 (m, 2P, P_M,M′_), −122.7 (m, 2P, P_x,x′_). ^31^P NMR (C_6_D_6_, 293 K): *δ* [ppm] = 117.2 (m, 1P, P_A_), 35.4 (m, 2P, P_M,M′_), −122.7 (m, 2P, P_x,x′_); (for coupling constants, see Supplementary Table 6). LIFDI-MS (toluene): 376.97 (100%, [M]^+^), 360.95 (40%, [M]^+^-CH_3_), 345.92 (20%, [M]^+^-(CH_3_)_2_), 314.95 (10% [M]^+^-P(CH_3_)_2_); analysis (calcd., found for C_12_H_21_FeP_5_): C (38.33, 38.37), H (5.63, 5.63).

#### Synthesis of [Cp*Fe(η^4^-P_5_Me^*i*^Pr)] (**3d**)

To a solution of **2c** (0.15 mmol, 90 mg, 1 eq) in 10 mL DME. 0.03 mL of isopropyl iodide (0.15 mmol, 1 eq) was added. The brownish solution was stirred overnight, all volatiles were removed in vacuum and the brown residue was dissolved in *n*-hexane and filtered through a frit. The resulting solution was concentrated to 3 mL and stored at −30 °C. Dark red blocks of [Cp*Fe(η^4^-P_5_^*i*^PrMe)] (**3d**) could be isolated after 2 days. Yield: 43 mg, (0.106 mmol, 72% yield). ^1^H NMR (C_6_D_6_, 293 K): δ [ppm] = 2.40 (m, 1H, -CH(CH_3_)_2_), 1.59 (s, 15H, C_5_(CH_3_)_5_), 1.01 (dd, 6H, -CH(CH_3_)_2_, ^3^*J*_H-H_ = 7.1 Hz, ^3^*J*_P-H_ = 15.0 Hz), 0.05 (d, 3H, -CH_3_, *J*_P-H_ = 10.5 Hz). ^31^P{^1^H} NMR (C_6_D_6_, 293 K): δ [ppm] = 151.8 (m, 1P, P_A_), 33.3 (m, 2P, P_M,M′_), −135.8 (m, 2P, P_x,x′_). ^31^P NMR (C_6_D_6_, 293 K): δ [ppm] = 151.8 (m, 1P, P_A_), 33.3 (m, 2P, P_M,M′_), −135.8 (m, 2P, P_x,x′_); (for coupling constants see Supplementary Table 7). FD-MS (toluene): 404.00 (100%, [M]^+^); analysis (calcd., found for C_14_H_25_FeP_5_): C (41.61, 41.60), H (6.24, 6.11).

#### Synthesis of [Cp*Fe(η^4^-P_5_^*t*^BuMe)] (**3e**)

Method A: A solution of **2d** (0.2 mmol, 152.5 mg, 1 eq) in 10 mL THF was cooled to −80 °C and a 1.005 molar solution of MeI in DME (0.2 mmol, 0.20 mL, 1 eq) was added. A colour change from green to brown occurred and a colourless solid was formed. The mixture was allowed to stir overnight and reach room temperature. The solvent was removed under reduced pressure and a brown solution was extracted with 10 mL toluene and filtered over diatomaceous earth. The solvent was removed in vacuo. The brown residue was dissolved in 2 mL THF, layered with 4 mL *n*-hexane and stored at –30 °C. [Cp*Fe(η^4^-P_5_^*t*^BuMe)] **3e** was formed as dark green blocks after 1 day. Yield: 71.1 mg (0.17 mmol, 85%).

Method B: [K(dme)_2_]_2_[Cp*Fe(η^4^-P_5_)] (**1**′) (0.4 mmol, 313.8 mg, 1 eq) was dissolved in 10 mL THF, cooled to −80 °C and 0.05 mL ^*t*^BuI (0.4 mmol, 1 eq) was added. The reaction mixture was stirred for 10 min and 0.18 mL of a 1.11 molar solution of MeI (0.2 mmol, 1 eq) in Et_2_O was added. The resulting brown solution was stirred overnight and the solvent was partly reduced in vacuo. The performed ^31^P{^1^H} NMR experiment (Supplementary Fig. 70) of the reaction in THF/C_6_D_6_-capillary matches the spectroscopic data of method A and shows a quantitative formation of **3e**. ^1^H NMR (C_6_D_6_, 293 K): *δ* [ppm] = 1.74 (m, 3H, -CH_3_), 1.62 (s, 15H, C_5_(CH_3_)_5_), 0.26 (d, 9H, ^3^*J*_P-H_ = 14.5 Hz, -C(CH_3_)_3_). ^31^P{^1^H} NMR (C_6_D_6_, 293 K): *δ* [ppm] = 151.9 (m, 1P, P_A_), 47.0 (m, 2P, P_M,M′_), −117.2 (m, 2P, P_x,x′_). ^31^P NMR (C_6_D_6_, 293 K): *δ* [ppm] = 151.9 (m, 1P, P_A_), 47.0 (m, 2P, P_M,M′_), −117.2 (m, 2P, P_x,x′_): (for coupling constants, see Supplementary Table 8). LIFDI-MS (toluene): 418.02 (100%, [M]^+^); analysis (calcd., found for C_15_H_27_FeP_5_): C (43.09, 43.63), H (6.51, 6.36).

#### Synthesis of [Cp*Fe(η^4^-P_5_PhMe)] (**3f**)

Compound **1** (0.4 mmol, 138.4 mg, 1 eq) was dissolved in 20 mL of DME, cooled to −50 °C and 0.22 mL of a 1.9 molar solution of PhLi (0.4 mmol, 1 eq) in dibutyl ether was added. The solution was stirred for 2 h and allowed to reach room temperature. To the brown solution, a 1.11 molar solution of MeI (0.4 mmol, 0.36 mL, 1 eq) in Et_2_O was added. The solution was stirred for 18 h, the solvent removed under reduced pressure, the remaining brown residue extracted with 3 × 5 mL of *n*-hexane and filtered over diatomaceous earth. The volume was reduced in vacuo and the solution stored at −30 °C. Compound **3f** was isolated after 3 days as dark green plates. Yield: 132.0 mg (0.30 mmol, 75%). ^1^H NMR (C_6_D_6_, 293 K): *δ* [ppm] = 6.82 (m, 2H, -C_6_H_5_), =6.71 (m, 3H, -C_6_H_5_), 1.95 (dt, 3H, ^2^*J*_P-H_ = 10.8 Hz, ^3^*J*_P-H_ = 5.4 Hz, -CH_3_), 1.64 (s, 15H, C_5_(CH_3_)_5_). ^31^P{^1^H} NMR (C_6_D_6_, 293 K): *δ* [ppm] = 117.7 (m, 1P, P_A_), 40.5 (m, 2P, P_M,M′_), −109.5 (m, 2P, P_x,x′_). ^31^P NMR (C_6_D_6_, 293 K): *δ* [ppm] = 117.7 (m, 1P, P_A_), 40.5 (m, 2P, P_M,M′_), −109.5 (m, 2P, P_x,x′_); (for coupling constants, see Supplementary Table 9). LIFDI-MS (toluene): 437.96 (100%, [M]^+^); analysis (calcd., found for C_17_H_23_FeP_5_): C (46.61, 47.09), H (5.29, 5.26).

#### Synthesis of PR′R″R‴ (**4a-c**)

Compound **3d** (0.1 mmol, 40.4 mg, 1 eq), **3e** (0.2 mmol, 83.6 mg, 1 eq) or **3f** (0.1 mmol, 43.8 mg, 1 eq) were dissolved in DME and cooled to −50 °C. To the brownish-green solution, a −50 °C cold solution of KBnz^50^ (0.1 mmol, 13.0 mg, 1equ. for **3d, 3f**, 0.2 mmol, 26.0 mg, 1equ. for **3e**) in DME was added. The colour changed to dark red and rapidly back to brownish-green. The solution was stirred overnight and allowed to reach room temperature. The solvent was slowly removed under reduced pressure (note: in order to avoid the loss of the desired phosphine by removal of the solvent, lower boiling solvents like THF can be used for the reaction). The oily residue was extracted with *n*-pentane (3 × 5 mL) and decanted off from the remaining solid (compound **5**, see below). The solvent was removed in vacuo and compound **4a-c** (**a**: R′ = Me, R″ = ^*i*^Pr, R‴ = Bnz; **b**: R′ = ^*t*^Bu, R″ = Me, R‴ = Bnz; **c**: R′ = Ph, R″ = Me, R‴ = Bnz) can be isolated as a viscous liquid. Yield: **4a**: 14.8 mg (0.082 mmol, 82%), **4b**: 21.5 mg (0.11 mmol, 55%), **4c**: 17.1 mg (0.08 mmol, 80%).

^1^H NMR: **4a** (C_6_D_6_, 293 K): *δ* [ppm] = 7.07 (m, 5H, -CH_2_-C_6_H_5_), 2.75 (dd, 1H, ^3^*J*_P-H_ = 13.14 Hz, ^3^*J*_H-H_ = 3.51 Hz, -CH_2_-Ph), 2.41 (dd, 1H, ^3^*J*_P-H_ = 13.18 Hz, ^3^*J*_H-H_ = 1.69 Hz, -CH_2_-Ph), 1.28 (m, 1H, -(CH)-CH_3_), 0.99 (dd, 3H, ^3^*J*_P-H_ = 14.1 Hz, ^2^*J*_H-H_ = 7.0 Hz, -(CH)-(CH_3_)_2_, 0.91 (dd, 3H, ^3^*J*_P-H_ = 12.2 Hz, ^2^*J*_H-H_ = 6.9 Hz, -(CH)-(CH_3_)_2_), 0.73 (d, 9H, ^2^*J*_P-H_ = 3.5 Hz, -CH_3_); **4b** (C_6_D_6_, 293 K): *δ* [ppm] = 7.13 (m, 8H, -CH_2_-C_6_H_5_), 2.76 (dd, 1H, ^3^*J*_P-H_ = 12.2 Hz, ^3^*J*_H-H_ = 3.9 Hz, -CH_2_-Ph), 2.33 (d, 1H, ^3^*J*_P-H_ = 13.0 Hz, ^3^*J*_H-H_ = 4.1 Hz, -CH_2_-Ph), 0.94 (d, 9H, ^3^*J*_P-H_ = 11.2 Hz, -C(CH_3_)_3_), 0.71 (d, 3H, ^2^*J*_P-H_ = 3.5 Hz, -CH_3_); **4c** (C_6_D_6_, 293 K): *δ* [ppm] = 7.21 (m, 5H, -CH_2_-C_6_H_5_), 7.21 (m, 5H, -CH_2_-C_6_H_5_), 2.95 (dd, 2H, ^2^*J*_P-H_ = 13.2 Hz, ^3^*J*_H-H_ = 2.9 Hz, -CH_2_-Ph), 2.79 (d, 1H, ^3^*J*_P-H_ = 13.0 Hz, -CH_2_-Ph), 1.10 (d, 3H, ^2^*J*_P-H_ = 4.0 Hz, -CH_3_).

^31^P{^1^H} NMR: **4a** (C_6_D_6_, 293 K): *δ* [ppm] = −18.5 (s); **4b** (C_6_D_6_, 293 K): *δ* [ppm] = −7.00 (s); **4c** (C_6_D_6_, 293 K): *δ* [ppm] = −29.6 (s).

^31^P NMR: **4a** (C_6_D_6_, 293 K): *δ* [ppm] = −18.5 (s); **4b** (C_6_D_6_, 293 K): *δ* [ppm] = −7.00 (s, br); **4c** (C_6_D_6_, 293 K): *δ* [ppm] = −29.6 (s, br).

^13^C{^1^H} NMR: **4a** (C_6_D_6_, 293 K): *δ* [ppm] = 138.5 (d, ^2^*J*_P-C_ = 4.1 Hz, Ph), 129.1 (d, ^3^*J*_P-C_ = 5.4 Hz, Ph), 128.2 (s, Ph), 125.5 (d, ^4^*J*_P-C_ = 2.1 Hz, Ph), 34.8 (d, ^1^*J*_P-C_ = 18.64 Hz, -(CH_2_)-Ph), 26.6 (d, ^2^*J*_P-C_ = 11.71 Hz, -(CH)-(CH_3_)_2_), 19.2 (d, ^1^*J*_P-C_ = 17.1 Hz, -(CH)-(CH_3_)_2_), 18.3 (d, ^2^*J*_P-C_ = 12.8 Hz, -(CH)-(CH_3_)_2_), 8.1 (d, ^1^*J*_P-C_ = 20.2 Hz, -CH_3_); **4b** (C_6_D_6_, 293 K): *δ* [ppm] = 139.1 (d, ^2^*J*_P-C_ = 6.9 Hz, Ph), 129.3 (d, ^3^*J*_P-C_ = 6.3 Hz, Ph), 128.3 (d, ^5^*J*_P-C_ = 0.7 Hz, Ph), 125.5 (d, ^4^*J*_P-C_ = 2.3 Hz, Ph), 33.0 (d, ^1^*J*_P-C_ = 20.8 Hz, -(CH_2_)-Ph), 27.0 (d, ^1^*J*_P-C_ = 13.6 Hz, -C(CH_3_)_3_), 26.5 (d, ^2^*J*_P-C_ = 13.6 Hz, -C(CH_3_)_3_), 6.2 (d, ^1^*J*_P-C_ = 23.0 Hz, -CH_3_); **4c** (C_6_D_6_, 293 K): *δ* [ppm] = 140.1 (d, ^1^*J*_P-C_ = 17.9 Hz, Ph), 137.7 (d, ^2^*J*_P-C_ = 4.5 Hz, Ph), 131.6 (d, ^1^*J*_P-C_ = 18.6 Hz, Ph), 129.1 (d, ^3^*J*_P-C_ = 5.4 Hz, Ph), 128.2 (d, ^2^*J*_P-C_ = 11.2 Hz, Ph), 128.1 (s, Ph), 128.1 (s, Ph), 125.7 (d, ^3^*J*_P-C_ = 5.4 Hz, Ph), 38.3 (d, ^1^*J*_P-C_ = 17.1 Hz, -(CH_2_)-Ph), 10.2 (d, ^1^*J*_P-C_ = 17.1 Hz, -CH_3_).

#### Synthesis of SPR′R″R‴ (**4a′-c′**)

Compound **4a-c** (0.1 mmol, 1 eq) were synthesized according to the previous procedure, dissolved in 10 mL of n-pentane, respectively, added to a solution of sulfur (0.1 mmol, 3.2 mg, 1 eq) in 10 mL n-pentane and stirred for 4 h at room temperature. The volume was reduced under reduced pressure and stored at −30 °C. Colourless crystals of **4a′-c′** (**a**: R′ = Me, R″ = iPr, R‴ = Bnz; **b**: R′ = tBu, R″ = Me, R‴ = Bnz; **c**: R′ = Ph, R″ = Me, R‴ = Bnz) were formed after 2 days. Yield: **4a′**: 16.0 mg (0.089 mmol, 89%), **4b′**: 17.5 mg (0.08 mmol, 80%), **4c′**: 17.1 mg (0.08 mmol, 80%).

^1^H NMR: **4a′** (C_6_D_6_, 293 K): *δ* [ppm] = 7.05 (m, 5H, -CH_2_-C_6_H_5_), 2.89 (t, 1H, ^3^*J*_P-H_ = 14.7 Hz, -CH_2_-Ph), 2.74 (dd, 1H, ^3^*J*_P-H_ = 13.8 Hz, ^3^*J*_H-H_ = 1.8 Hz, -CH_2_-Ph), 1.39 (m, 1H, -(CH)-CH_3_), 0.99 (dd, 3H, ^3^*J*_P-H_ = 17.8 Hz, ^2^*J*_H-H_ = 7.0 Hz, -(CH)-(CH_3_)_2_), 0.97 (d, 9H, ^2^*J*_P-H_ = 11.9 Hz, -CH_3_), 0.84 (dd, 3H, ^3^*J*_P-H_ = 17.5 Hz, ^2^*J*_H-H_ = 6.9 Hz, -(CH)-(CH_3_)_2_); **4b′** (C_6_D_6_, 293 K): *δ* [ppm] = 7.27 (m, 2H, -CH_2_-C_6_H_5_), 7.12 (m, 3H, -CH_2_-C_6_H_5_), 2.79 (m, 2H, -CH_2_-Ph), 0.97 (d, 3H, ^2^*J*_P-H_ = 11.8 Hz, -CH_3_), 0.95 (d, 9H, ^3^*J*_P-H_ = 15.5 Hz, -C(CH_3_)_3_); **4c′** (C_6_D_6_, 293 K): *δ* [ppm] = 7.60 (m, 2H, -Ph), 7.00 (m, 6H, -Ph), 6.91 (m, 2H, -Ph), 3.01 (d, 2H, ^2^*J*_P-H_ = 14.1 Hz, -CH_2_-Ph), 1.39 (d, 3H, ^2^*J*_P-H_ = 12.7 Hz, -CH_2_-Ph).

^31^P{^1^H} NMR: **4a′** (C_6_D_6_, 293 K): *δ* [ppm] = 51.3 (s); **4b′** (C_6_D_6_, 293 K): *δ* [ppm] = 58.6 (s); **4c′** (C_6_D_6_, 293 K): *δ* [ppm] = 38.0 (s).

^31^P NMR: **4a′** (C_6_D_6_, 293 K): *δ* [ppm] = 51.3 (s, br); **4b′** (C_6_D_6_, 293 K): *δ* [ppm] = 58.6 (m); **4c′** (C_6_D_6_, 293 K): *δ* [ppm] = 38.0 (m).

^13^C{^1^H} NMR: **4a′** (C_6_D_6_, 293 K): *δ* [ppm] = 132.7 (d, ^2^*J*_P-C_ = 7.9 Hz, Ph), 129.7 (d, ^3^*J*_P-C_ = 4.9 Hz, Ph), 128.2 (d, ^4^*J*_P-C_ = 2.8 Hz, Ph), 126.8 (d, ^3^*J*_P-C_ = 3.3 Hz, Ph), 39.4 (d, ^1^*J*_P-C_ = 45.1 Hz, -(CH_2_)-Ph), 28.7 (d, ^1^*J*_P-C_ = 52.3 Hz, -(CH)-(CH_3_)_2_), 15.6 (d, ^2^*J*_P-C_ = 21.3 Hz, -(CH)-(CH_3_)_2_), 15.5 (d, ^1^*J*_P-C_ = 52.5 Hz, -CH_3_ 15.3 (d, ^2^*J*_P-C_ = 26.8 Hz, -(CH)-(CH_3_)_2_); **4b′** (C_6_D_6_, 293 K): *δ* [ppm] = 132.9 (d, ^2^*J*_P-C_ = 7.8 Hz, Ph), 130.6 (d, ^3^*J*_P-C_ = 5.7 Hz, Ph), 127.1 (d, ^5^*J*_P-C_ = 2.9 Hz, Ph), 127.1 (d, ^4^*J*_P-C_ = 3.2 Hz, Ph), 36.1 (d, ^1^*J*_P-C_ = 43.1 Hz, -(CH_2_)-Ph), 33.3 (d, ^1^*J*_P-C_ = 49.4 Hz, -C(CH_3_)_3_), 24.6 (d, ^2^*J*_P-C_ = 1.4 Hz, -C(CH_3_)_3_), 13.4 (d, ^1^*J*_P-C_ = 50.8 Hz, -CH_3_); **4c′** (C_6_D_6_, 293 K): *δ* [ppm] = 131.0 (d, ^2^*J*_P-C_ = 9.9 Hz, Ph), 130.8 (d, ^4^*J*_P-C_ = 2.8 Hz, Ph), 130.0 (d, ^3^*J*_P-C_ = 5.2 Hz, Ph), 126.8 (d, ^3^*J*_P-C_ = 3.7 Hz, Ph), 43.8 (d, ^1^*J*_P-C_ = 49.0 Hz, -(CH_2_)-Ph), 18.9 (d, ^1^*J*_P-C_ = 57.3 Hz, -C(CH_3_)_3_).

**4a′**: EI-MS (CH_2_Cl_2_): 212.08 (37%, [M]^+^), 91.05 (100% [Bnz]^+^), 170.03 (46% [M-C_3_H_6_]^+^).

**4b′**: GC-MS (CH_2_Cl_2_): 226.09 (20%, [M]^+^), 91.06 (100% [Bnz]^+^),170.03 (44% [M-^*t*^Bu + H]^+^).

**4c′**: EI-MS (CH_2_Cl_2_): 246.06 (44%, [M]^+^), 155.01 (100% [M-Bnz]^+^), 91.06 (25% [Bnz]^+^).

#### Targeted synthesis of [K(18c6)(thf)][Cp*Fe(η^4^-P_4_)] (**5**)

Compound **3c** (0.2 mmol, 75.2 mg, 1 eq), KBnz (0.2 mmol, 26.0 mg, 1 eq) and 18-crown-6 (0.2 mmol, 52.9 mg, 1 eq) were dissolved separately in THF and cooled to −80 °C. The potassium benzyl solution is added to compound **3c**, stirred for 2 min and 18c6 is added afterwards. The mixture was allowed to stir overnight and reach room temperature. The solvent of the resulting brown solution was removed in vacuo. The by-product PMe_2_Bnz was removed by washing the oily residue three times with 5 mL of *n*-pentane, resulting a greenish powder, which was dissolved in THF and layered with *n*-hexane. The solution was stored at −30 °C and compound **5** was isolated as highly air-sensitive green/turquoise plates/blocks after 1 week. Yield: 111.9 mg (0.16 mmol, 81%). ^**1**^H NMR (THF-d_8_, 293 K): *δ* [ppm] = 3.73 (s, 26 H, 18-crown-6), 2.26 (s, 15H, C_5_(CH_3_)_5_). ^31^P{^1^H} NMR (THF-d_8_, 293 K): *δ* [ppm] = 118.9 (s), ^31^P NMR (THF-d_8_, 293 K): *δ* [ppm] = 118.9 (s). ESI-MS (anion, DME): *m/z* = 314.95 (100%, [M]^−^); analysis (calcd., found for C_22_H_39_FeP_4_KO_6_ ∙ (THF)_0.75_: C (44.65, 44.28), H (6.74, 6.67).

#### Synthesis of PMe_2_Bnz and regeneration of [Cp*Fe(η^5^-P_5_)] (**1**) in a cyclic process

Compound **1** (3.00 mmol, 1.04 g, 1 eq) was dissolved in 100 mL tetraglyme, 1.88 mL of a 1.6 molar solution of MeLi (3.00 mmol, 1 eq) in Et_2_O was added and the resulting solution was allowed to stir for 5 min. To the reaction mixture, 2.80 mL of a 1.07 molar solution of MeI (3.00 mmol, 1 eq) in Et_2_O was slowly added at room temperature and stirred for 5 min. The diethylether of the stock solutions was removed under reduced pressure. The reaction mixture was cooled to −30 °C, a solution of KBnz (3.00 mmol, 390.7 mg, 1 eq) in 50 mL tetraglyme was added and the reaction was stirred for 30 min. The cooling bath was removed and the phosphine PMe_2_Bnz was distilled off under reduced pressure (1 × 10^−3^ mbar, 55 °C). White phosphorus (3.00 mmol, 371.7 mg, 1 eq) was added to the remaining solution and the reaction mixture was heated under reflux for 1 h at 275 °C. The described steps were repeated two more times. The starting material **1** was isolated after column chromatographic workup (silica gel, *n*-hexane, 20 × 2 cm) in overall 69% yield (2.07 mmol, 2.07 g) (Supplementary Fig. 66). The phosphine PMe_2_Bnz was isolated as a pyrophoric colourless liquid in an overall yield of 79% (2.37 mmol, 315.0 mg) (see Supplementary Fig. 67, Supplementary Table 40).

## Supplementary information

Supplementary Information

## Data Availability

The X-ray crystallographic coordinates for structures reported in this study have been deposited at the Cambridge Crystallographic Data Centre (CCDC), under deposition numbers CCDC-2041977 (**2c**), CCDC-2041978 (**2d**), CCDC-2083624 (**2e**), CCDC-2041979 (**3a**), CCDC-2041980 (**3b**), CCDC-2083625 (**3c**), CCDC-2083626 (**3d**), CCDC-2041981 (**3e**), CCDC-2083627 (**3f**), CCDC-2083628 (**4a′**), CCDC-2083629 (**4b′**), CCDC-2083630 (**4c′**) and CCDC-2083631 (**5**). These data can be obtained free of charge from The Cambridge Crystallographic Data Centre via www.ccdc.cam.ac.uk/data_request/cif. All other data are available in the main text or in the Supplementary Information.
