# Supplementary material for: Pentaphosphaferrocene-mediated synthesis of asymmetric organo-phosphines starting from white phosphorus
Source: Nat Commun. 2021 Oct 1;12:5774. doi: 10.1038/s41467-021-26002-7 (PMC8486752; doi:10.1038/s41467-021-26002-7)
Supplement: Supplementary file 1 — Supplementary Information [file 41467_2021_26002_MOESM1_ESM.pdf]

Supplementary Information for

Pentaphosphaferrocene mediated Synthesis of Asymmetric Organo-Phosphines Starting from White Phosphorus

Stephan Reichl[1], Eric Mädl[1], Felix Riedlberger[1], Martin Piesch[1], Gábor Balázs[1], Michael Seidl[1], Manfred Scheer[1]*

[1]Institute of Inorganic Chemistry, University of Regensburg; Universitätsstraße 31, 93053 Regensburg, Germany.
*Corresponding author. Email: Manfred.Scheer@chemie.uni-regensburg.de

This PDF file includes:

1. NMR spectroscopic characterization 2
2. Crystallographic details 27
3. Data availability 49
4. Computational details 58
5. Supplementary References 63

Supplementary Figure 1-72
Supplementary Tables 1-46

NMR spectroscopic characterization

^{1}H NMR Spectra:

[Li(dme)$_3$][Cp*Fe(η^4-P_5Me)] (**2c**)

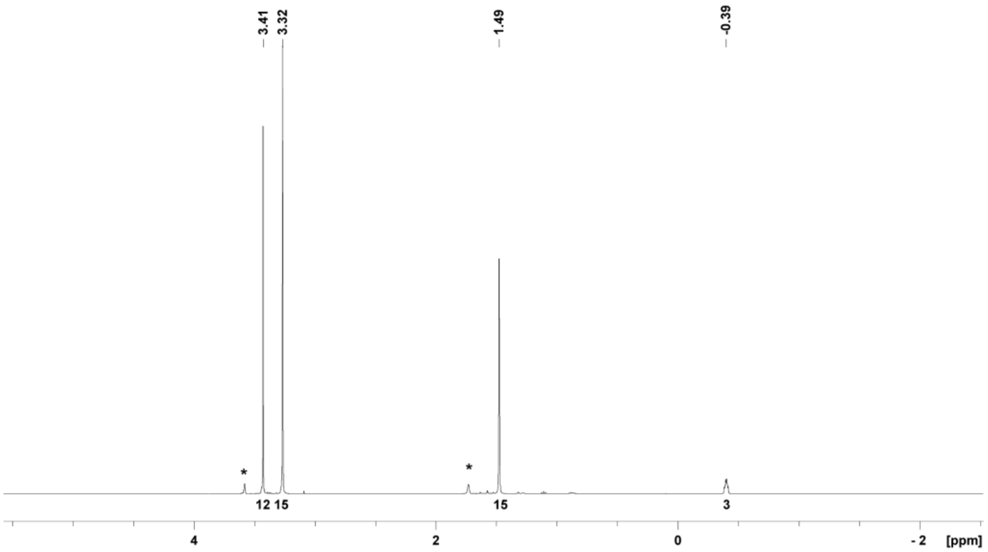

Supplementary Figure 1.
Experimental ^{1}H NMR (400.13 MHz, THF-d_8) spectrum of **2c** (* = THF).

[Li(12c4)$_2$][Cp*Fe(η^4-$P_5{}^t$Bu)] (**2d**)

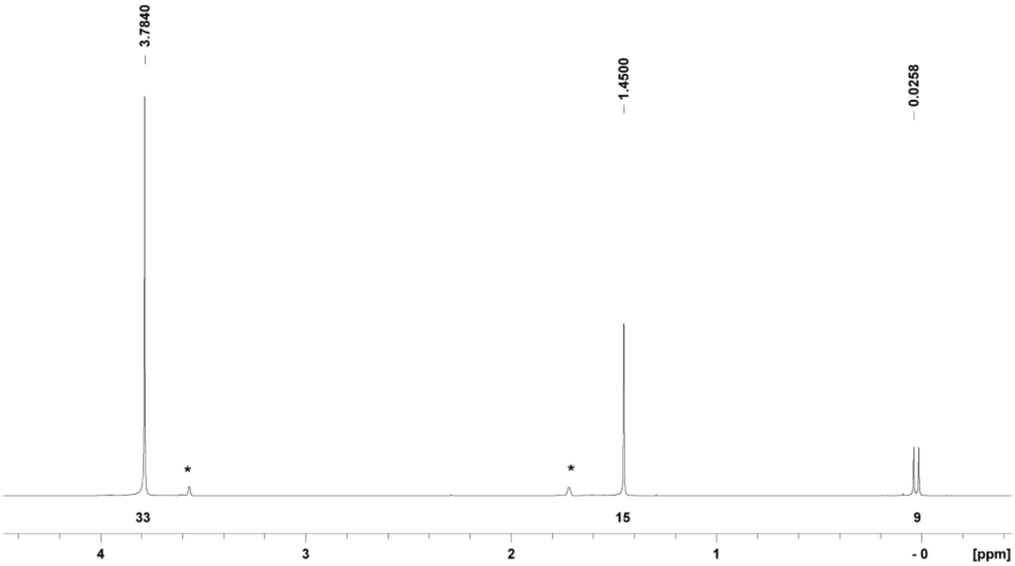

Supplementary Figure 2.
Experimental ^{1}H NMR (400.13 MHz, THF-d_8) spectrum of **2d** (* = THF).

[Li(12c4)(thf)][Cp*Fe(η^4-P_5Ph)] (**2e**)

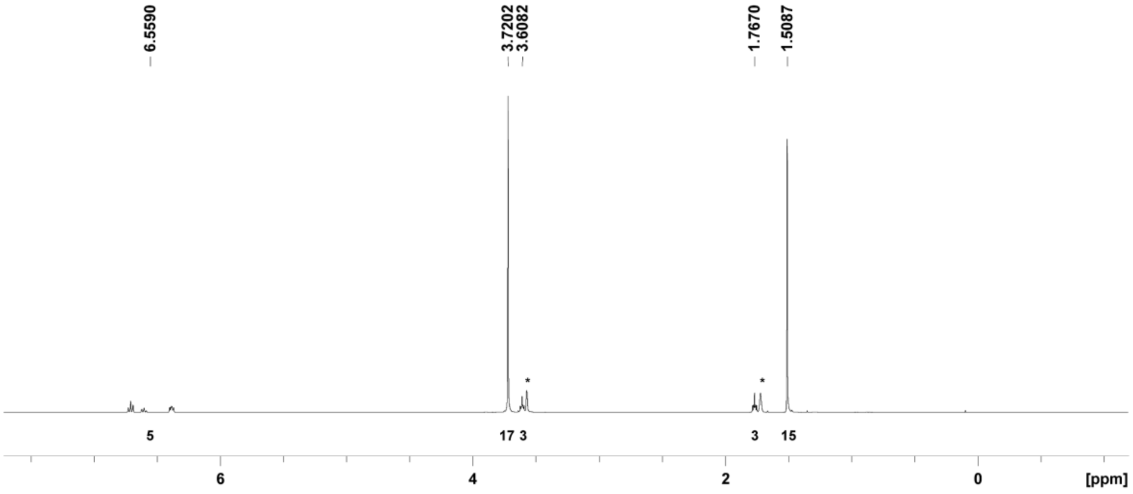

Supplementary Figure 3.
Experimental ^{1}H NMR (400.13 MHz, THF-d_8) spectrum of **2e** (* = THF).

[Cp*Fe(η^4-P_5NMe_2Me)] (**3a**)

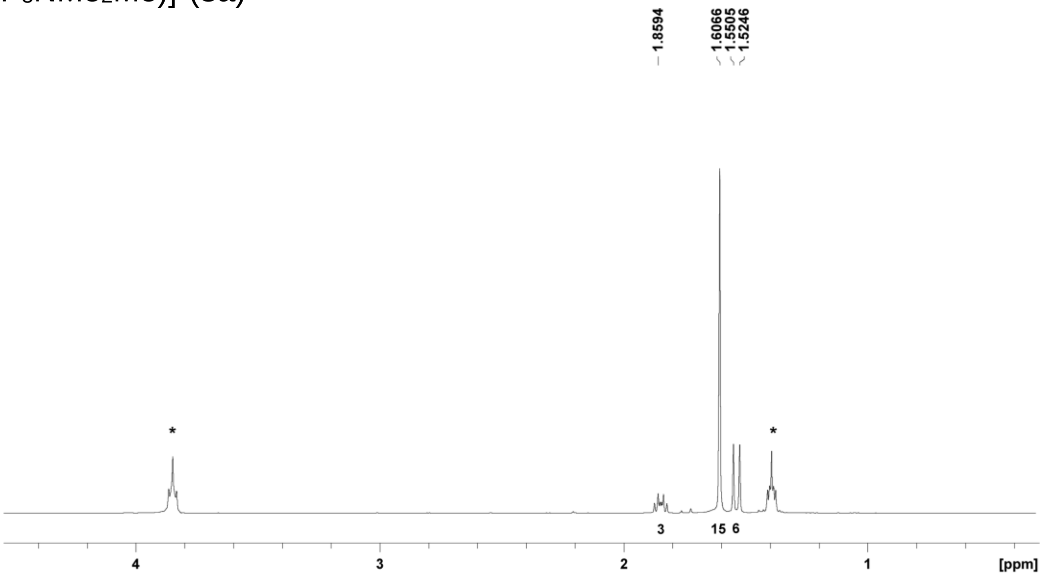

Supplementary Figure 4.
Experimental ^{1}H NMR (400.13 MHz, C_6D_6) spectrum of **3a** (* = THF).

[Cp*Fe{η4-P5(CH2SiMe3)Me}] (**3b**)

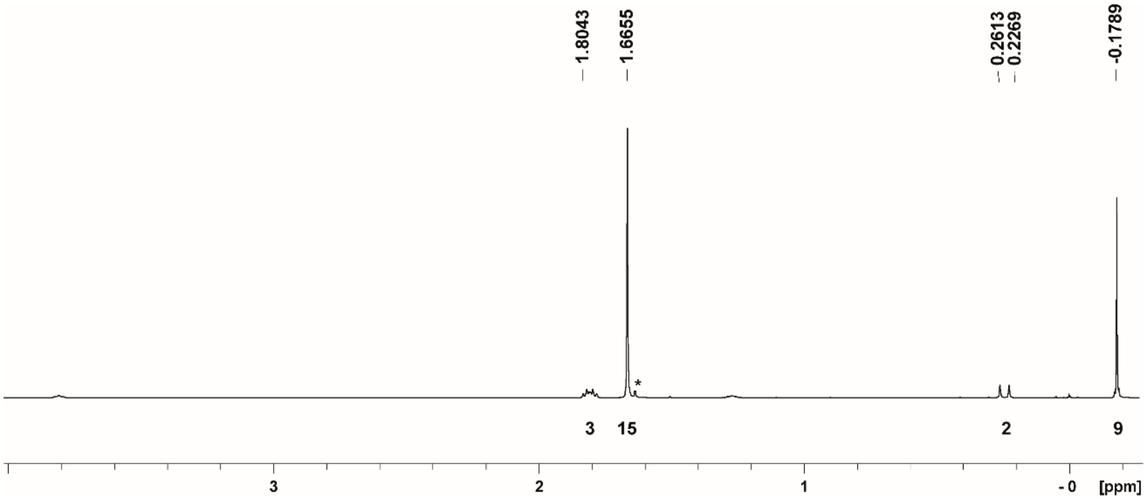

Supplementary Figure 5.
Experimental ^{1}H NMR (400.13 MHz, C_6D_6) spectrum of **3b** (* = traces of **I**).

[Cp*Fe(η4-P5Me2)] (**3c**)

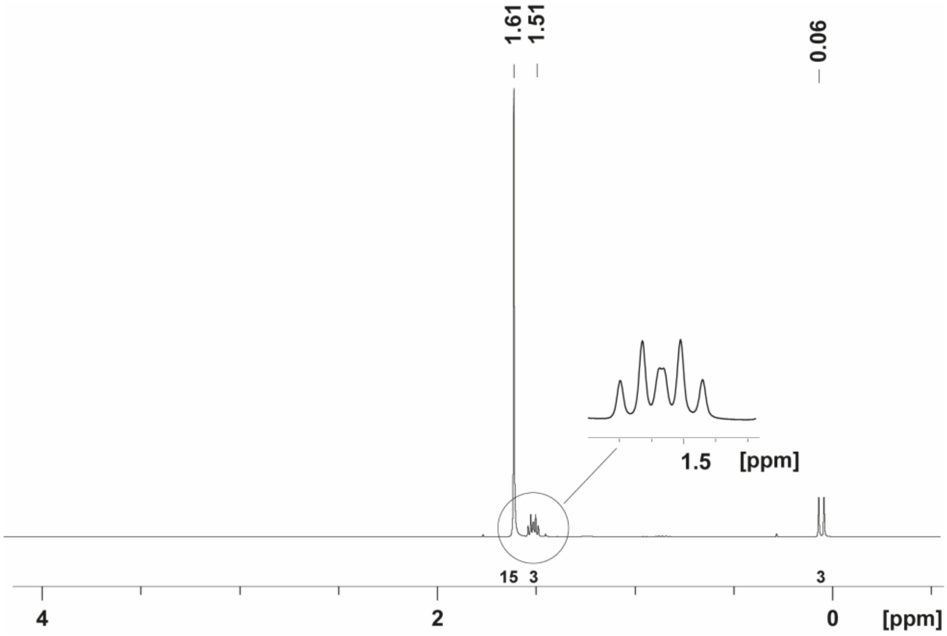

Supplementary Figure 6.
Experimental ^{1}H NMR (400.13 MHz, C_6D_6) spectrum of **3c**.

[Cp*Fe(η⁴-P₅MeⁱPr)] (**3d**)

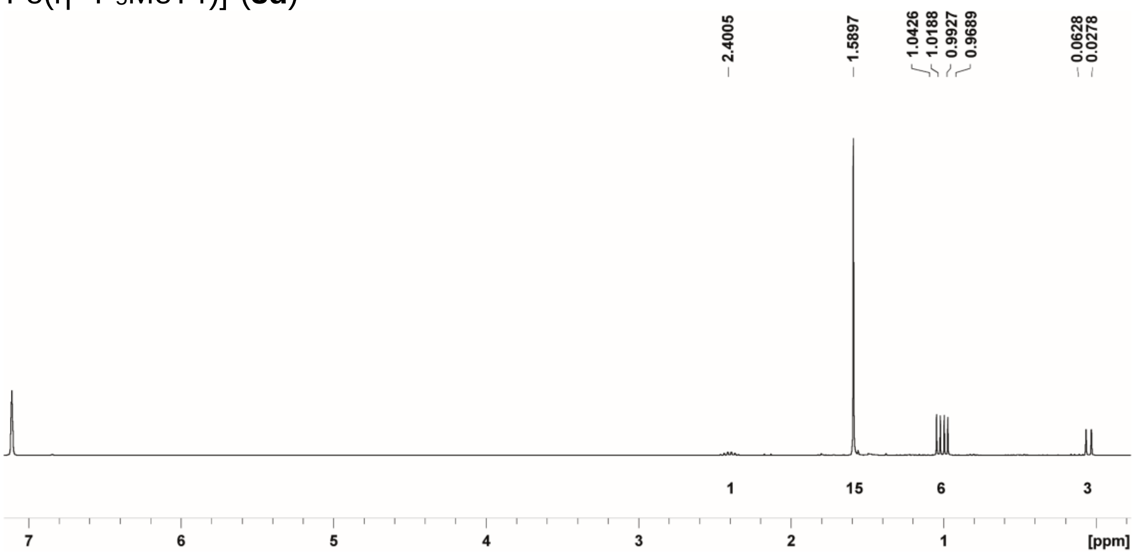

Supplementary Figure 7.
Experimental ^{1}H NMR (400.13 MHz, C_6D_6) spectrum of **3d**.

[Cp*Fe(η^4-P_5^{t}BuMe)] (**3e**)

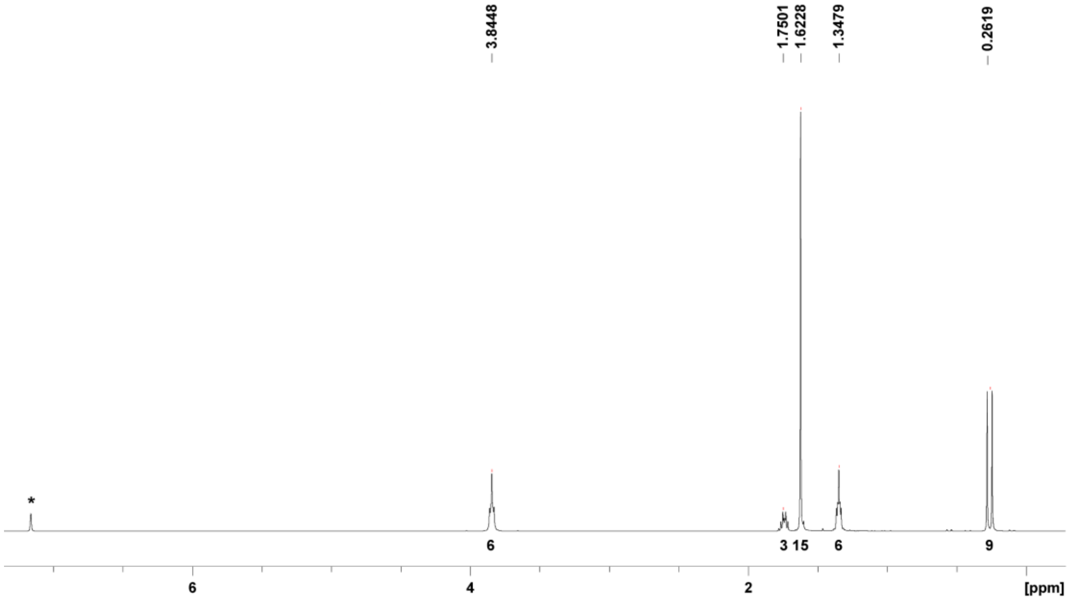

Supplementary Figure 8.
Experimental ^{1}H NMR (400.13 MHz, THF-d_8) spectrum of **3e** (* = residual solvent).

[Cp*Fe(η^4-P_5PhMe)] (**3f**)

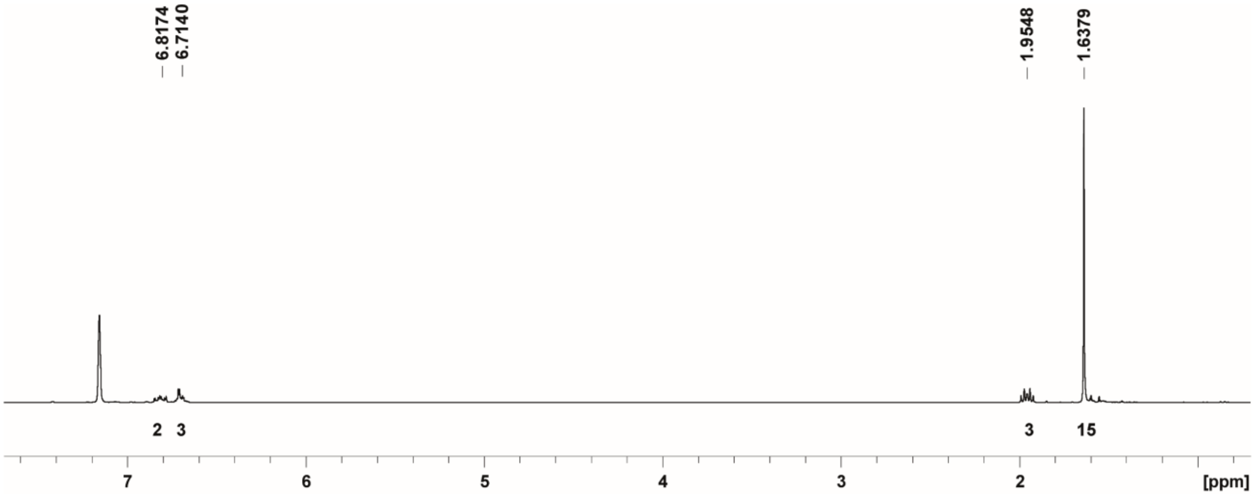

Supplementary Figure 9.
Experimental ^{1}H NMR (400.13 MHz, C_6D_6) spectrum of **3f**.

PMe^{i}PrBnz (**4a**)

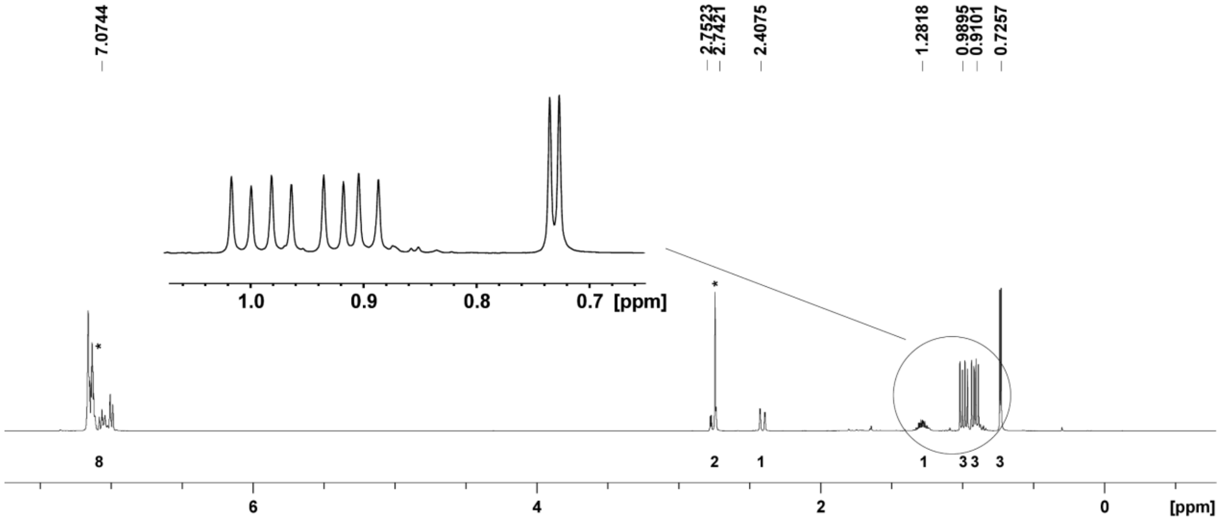

Supplementary Figure 10.
Experimental ^{1}H NMR (400.13 MHz, C_6D_6) spectrum of **4a** (* = 1,2-diphenylethane: decomposition product of KBnz).

P^{t}BuMeBnz (**4b**)

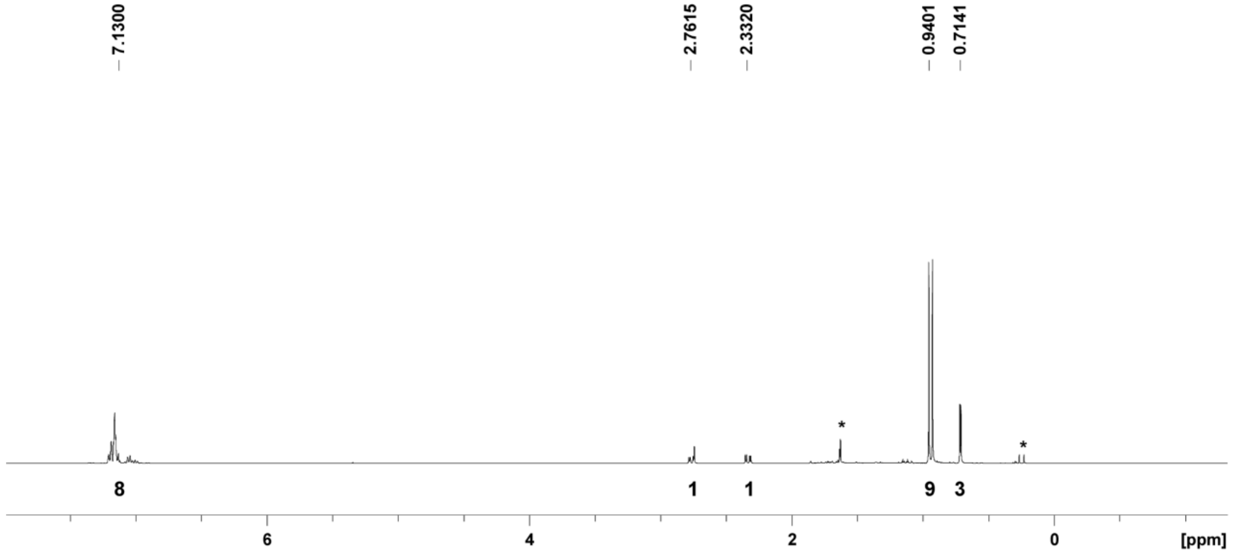

Supplementary Figure 11.
Experimental ^{1}H NMR (400.13 MHz, C_6D_6) spectrum of **4b** (* = starting material **3e**).

PPhMeBnz (**4c**)

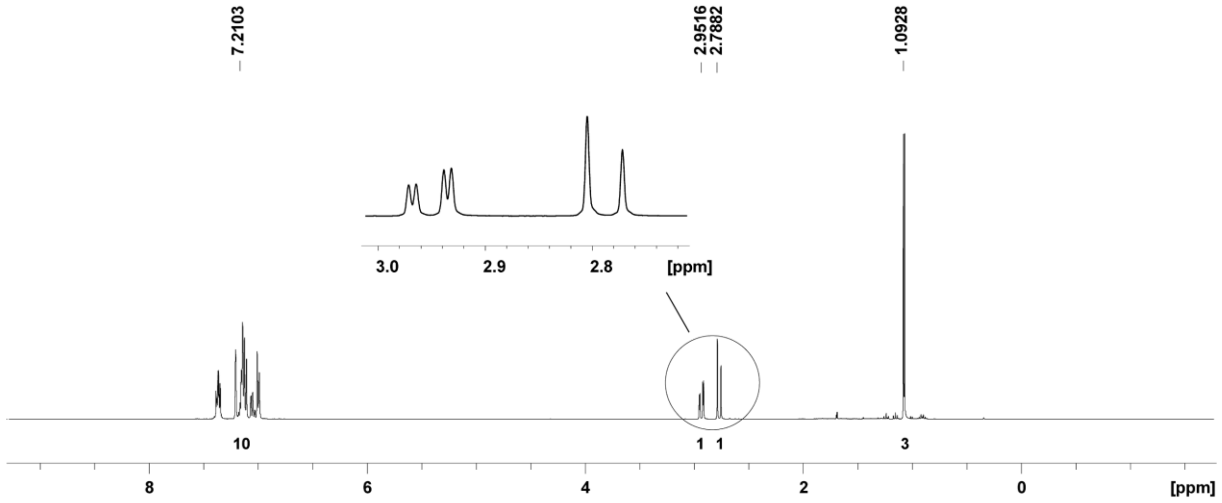

Supplementary Figure 12.
Experimental ^{1}H NMR (400.13 MHz, C_6D_6) spectrum of **4c**.

SPMe^{i}PrBnz (**4a’**)

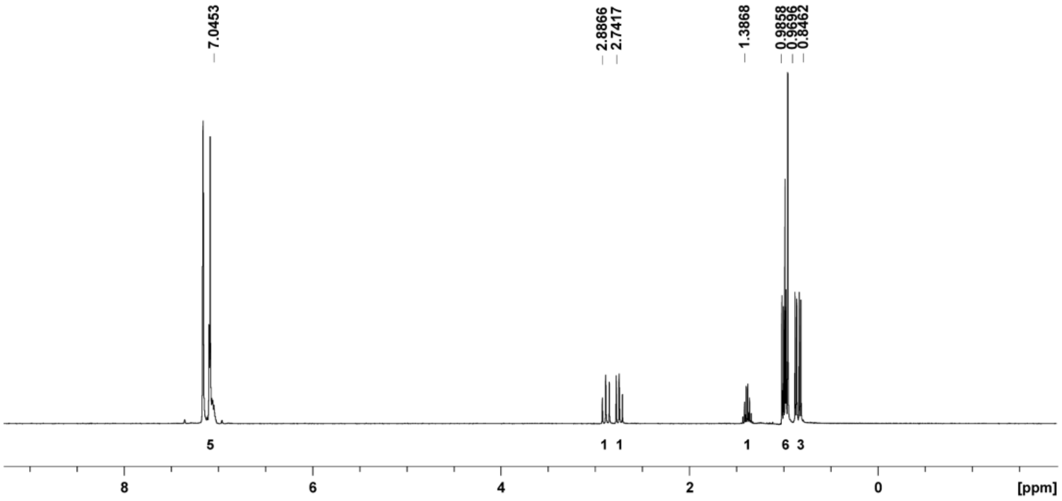

Supplementary Figure 13.
Experimental ^{1}H NMR (400.13 MHz, C_6D_6) spectrum of **4a’**.

SP^{t}BuMeBnz (**4b’**)

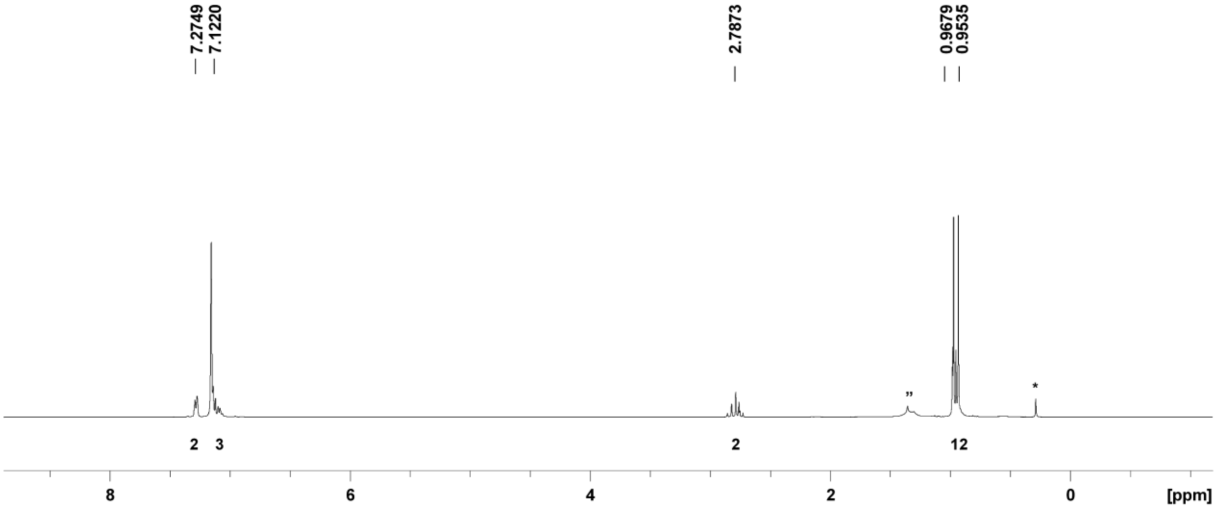

Supplementary Figure 14.
Experimental ^{1}H NMR (400.13 MHz, C_6D_6) spectrum of **4b’** (* = grease; “ = unidentified side product).

SPPhMeBnz (**4c’**)

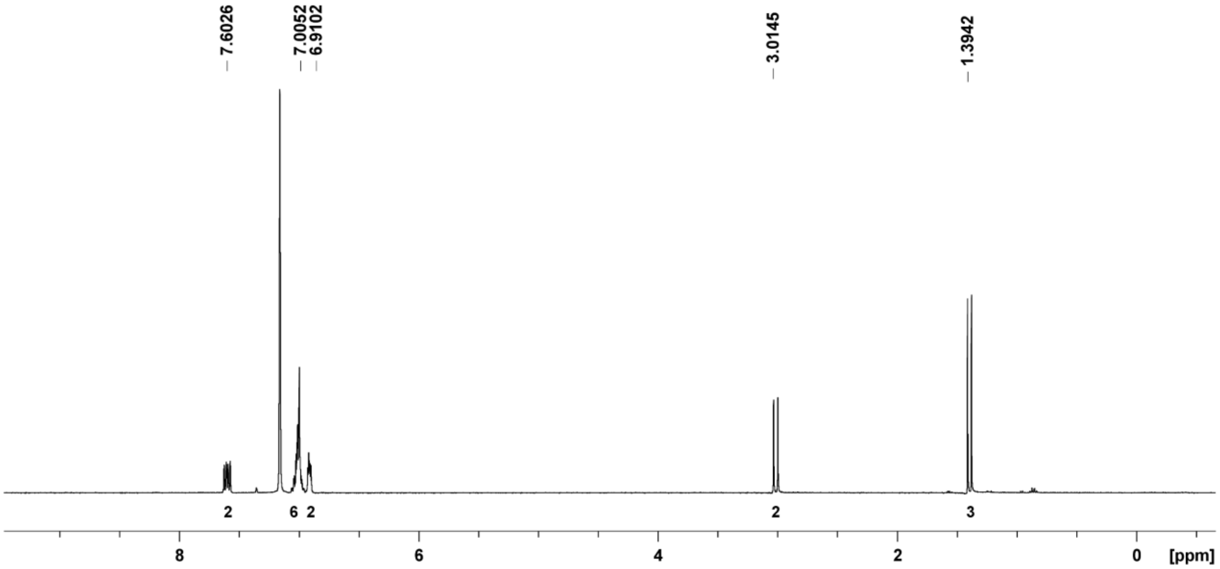

Supplementary Figure 15.
Experimental ^{1}H NMR (400.13 MHz, C_6D_6) spectrum of **4c’**.

[K(18c6)(thf)][Cp*Fe(η^4-P_4)] (**5**)

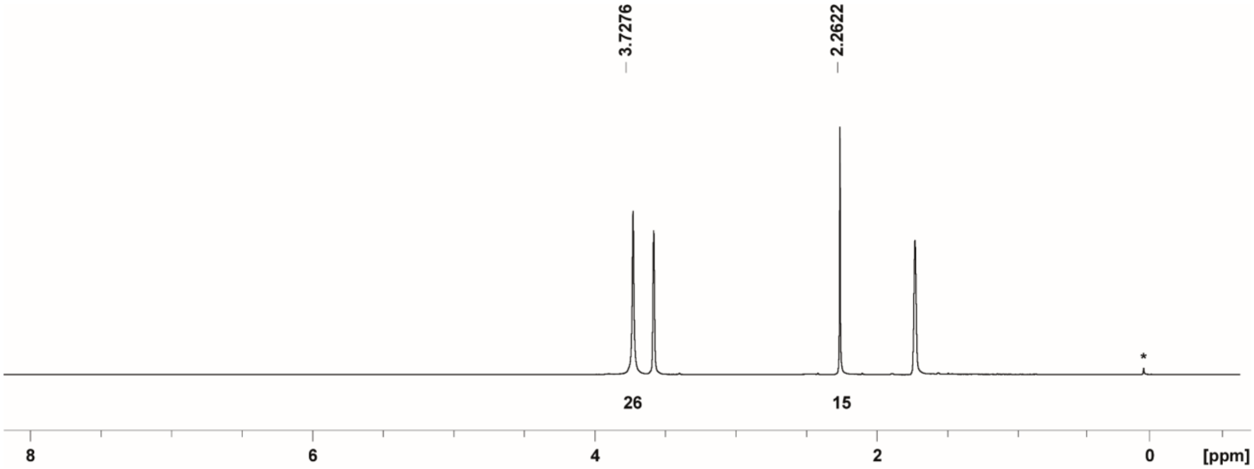

Supplementary Figure 16.
Experimental ^{1}H NMR (400.13 MHz, THF-d_8) spectrum of **5** (* = grease).

^{31}P NMR Spectra:

[Li(dme)$_3$][Cp*Fe(η^4-P$_5$Me)] (**2c**)

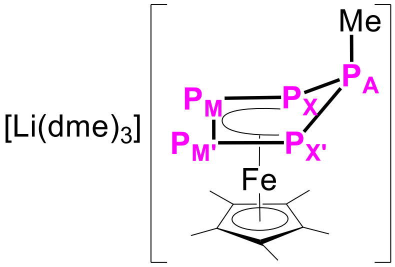

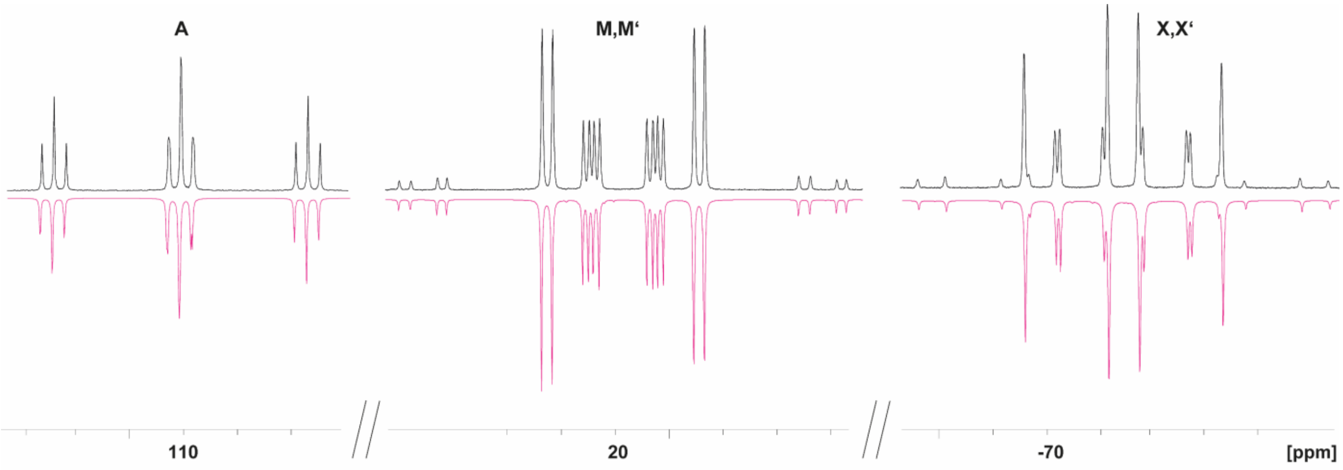

Supplementary Figure 17.
Experimental (top) and simulated (bottom) ^{31}P{^{1}H} NMR (161.98 MHz, THF-d$_8$) spectrum of **2c**.

Supplementary Table 1.
Chemical shifts and coupling constants obtained from the simulation (R-factor = 1.59 %) in Supplementary Figure 17.

J [Hz]				δ [ppm]	
$^{1}J_{P_A,P_X}$	275.51	$^{1}J_{P_M,P_X}$	394.28	X, X’	-71.5
$^{1}J_{P_A,P_{X'}}$	269.84	$^{1}J_{P_{M'},P_{X'}}$	367.94		
$^{2}J_{P_A,P_{M'}}$	-25.87	$^{2}J_{P_{M'},P_X}$	-27.00	M, M’	13.7
$^{2}J_{P_A,P_M}$	-25.97	$^{2}J_{P_M,P_{X'}}$	8.87		
$^{1}J_{P_M,P_{M'}}$	409.86	$^{2}J_{P_X,P_{X'}}$	-51.98	A	71.3

[Li(12c4)$_2$][Cp*Fe(η^4-$P_5{}^t$Bu)] (**2d**)

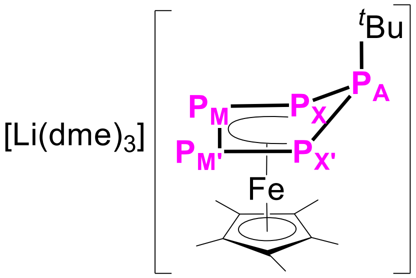

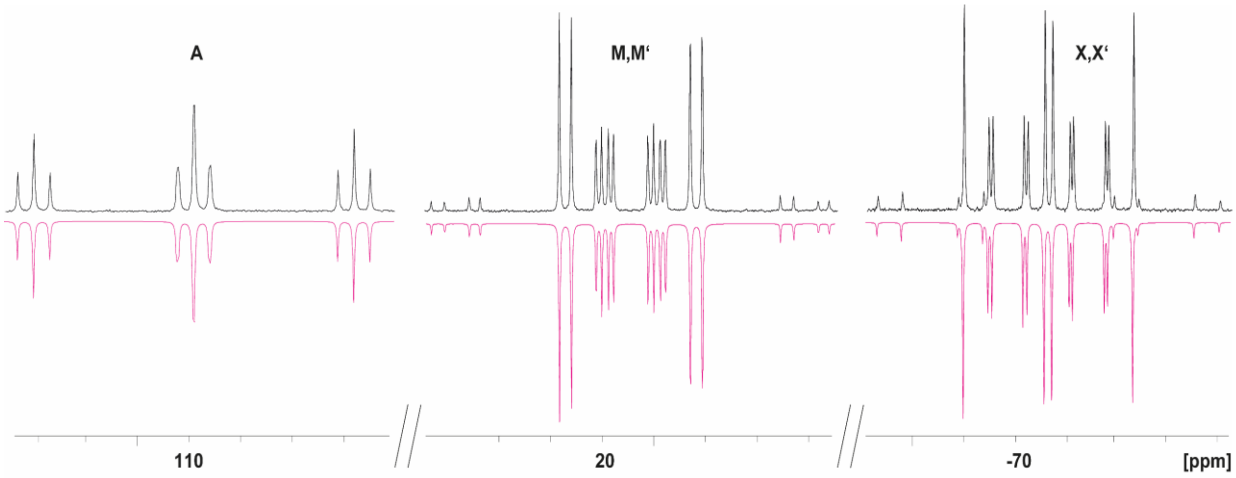

Supplementary Figure 18.
Experimental (top) and simulated (bottom) $^{31}P\{^1H\}$ NMR (161.98 MHz, THF-d$_8$) spectrum of **2d**.

Supplementary Table 2.
Chemical shifts and coupling constants obtained from the simulation (R-factor = 0.66 %) in Supplementary Figure 18.

J [Hz]				δ [ppm]	
$^1J_{P_A,P_X}$	339.07	$^1J_{P_M,P_X}$	384.46	X, X'	-70.2
$^1J_{P_A,P_{X'}}$	337.80	$^1J_{P_{M'},P_{X'}}$	365.20		
$^2J_{P_A,P_{M'}}$	-33.88	$^2J_{P_{M'},P_X}$	-13.62	M, M'	19.6
$^2J_{P_A,P_M}$	-34.26	$^2J_{P_M,P_{X'}}$	5.03		
$^1J_{P_M,P_{M'}}$	420.91	$^2J_{P_X,P_{X'}}$	-59.74	A	105.6

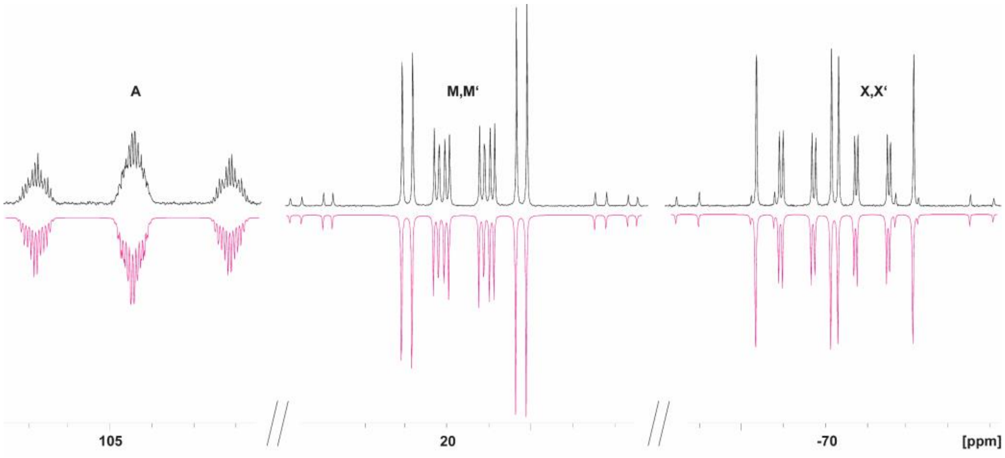

Supplementary Figure 19.

Experimental (top) and simulated (bottom) ^{31}P NMR (161.98 MHz, THF-d$_8$) spectrum of **2d**.

Supplementary Table 3.

Chemical shifts and coupling constants obtained from the simulation (R-factor = 1.52 %) in Supplementary Figure 19.

J [Hz]				*δ* [ppm]	
$^1J_{P_A,P_X}$	338.11	$^1J_{P_M,P_X}$	384.20	X, X'	-70.2
$^1J_{P_A,P_{X'}}$	338.76	$^1J_{P_{M'},P_{X'}}$	365.48		
$^2J_{P_A,P_{M'}}$	-33.45	$^2J_{P_{M'},P_X}$	-15.01	M, M'	19.6
$^2J_{P_A,P_M}$	-34.79	$^2J_{P_M,P_{X'}}$	6.50		
$^1J_{P_M,P_{M'}}$	420.73	$^2J_{P_X,P_{X'}}$	-59.41	A	105.6
$^3J_{P_A,H}$	10.76				

$[Li(12c4)(thf)][Cp^*Fe(\eta^4\text{-}P_5Ph)]$ (**2e**)

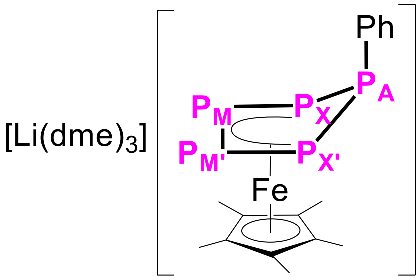

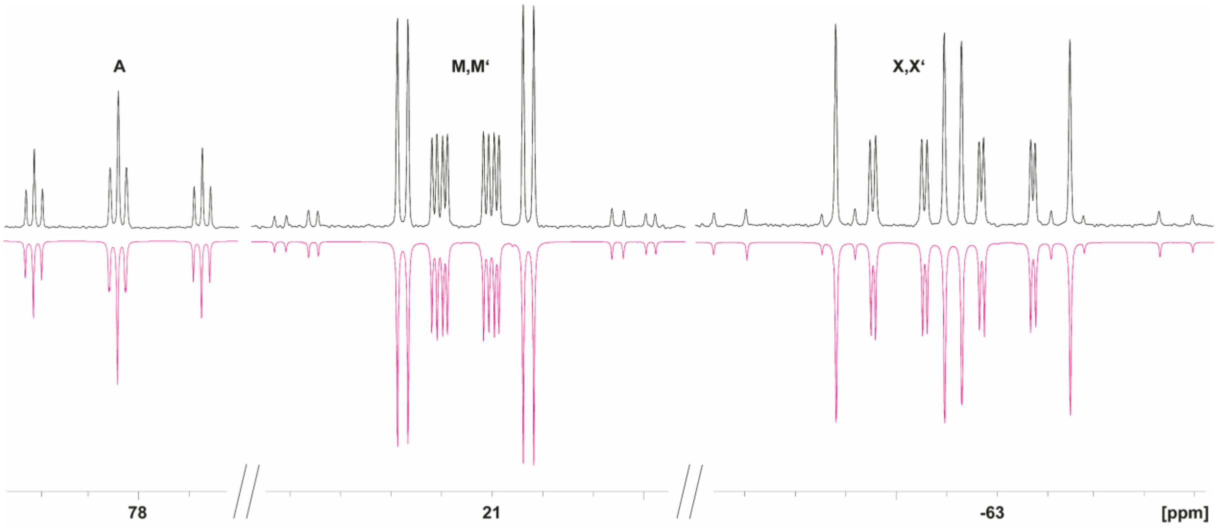

Supplementary Figure 20.
Experimental (top) and simulated (bottom) ^{31}P NMR (161.98 MHz, THF-d$_8$) spectrum of **2e**.

Supplementary Table 4.
Chemical shifts and coupling constants obtained from the simulation (R-factor = 4.89 %) in Supplementary Figure 20**.**

J [Hz]				δ [ppm]	
$^1J_{P_A,P_X}$	315.77	$^1J_{P_M,P_X}$	382.18	X, X’	-61.8
$^1J_{P_A,P_{X'}}$	318.37	$^1J_{P_{M'},P_{X'}}$	368.96		
$^2J_{P_A,P_{M'}}$	40.09	$^2J_{P_{M'},P_X}$	-4.06	M, M’	21.1
$^2J_{P_A,P_M}$	21.56	$^2J_{P_M,P_{X'}}$	-12.65		
$^1J_{P_M,P_{M'}}$	417.53	$^2J_{P_X,P_{X'}}$	55.50	A	79.3

[Cp*Fe(η^4-P_5NMe_2Me)] (**3a**)

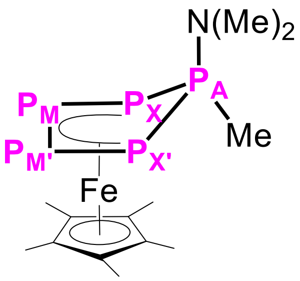

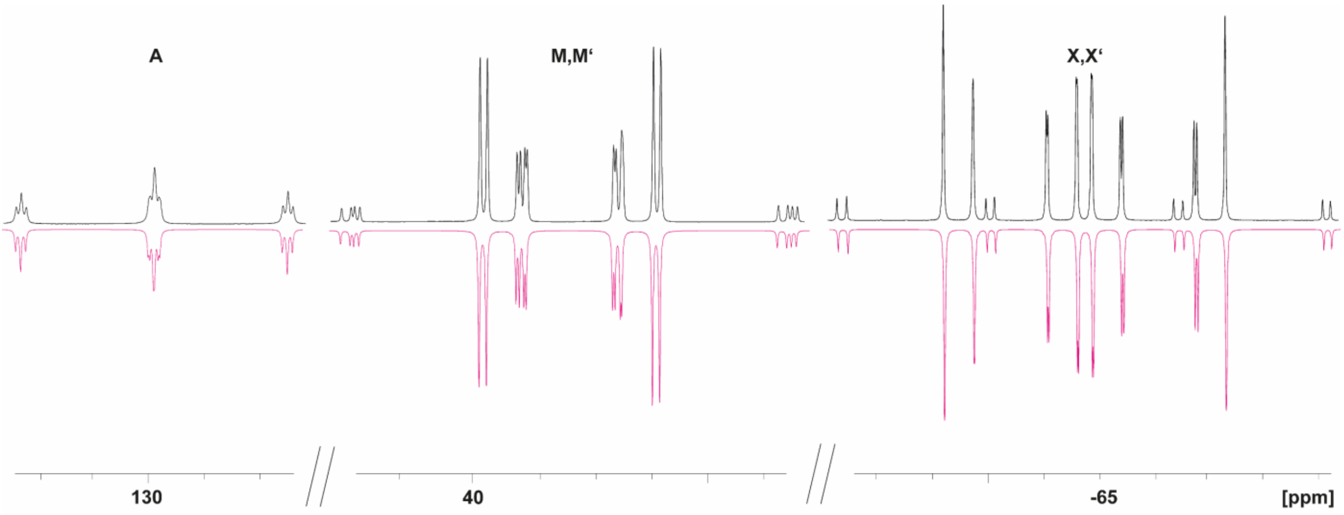

Supplementary Figure 21.

Experimental (top) and simulated (bottom) $^{31}P\{^1H\}$ NMR (161.98 MHz, C_6D_6) spectrum of **3a**.

Supplementary Table 5.

Chemical shifts and coupling constants obtained from the simulation (R-factor = 1.36 %) in Supplementary Figure 21.

J [Hz]				*δ* [ppm]	
$^1J_{P_A,P_X}$	426.72	$^1J_{P_M,P_X}$	412.85	X, X'	-64.5
$^1J_{P_A,P_{X'}}$	427.57	$^1J_{P_{M'},P_{X'}}$	415.74		
$^2J_{P_A,P_{M'}}$	18.52	$^2J_{P_{M'},P_X}$	-38.01	M, M'	37.2
$^2J_{P_A,P_M}$	13.55	$^2J_{P_M,P_{X'}}$	-31.93		
$^1J_{P_M,P_{M'}}$	377.81	$^2J_{P_X,P_{X'}}$	15.37	A	131.0

[Cp*Fe{η4-P5(CH2SiMe3)Me}] (**3b**)

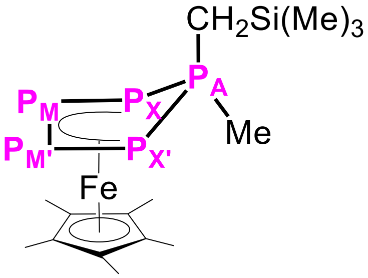

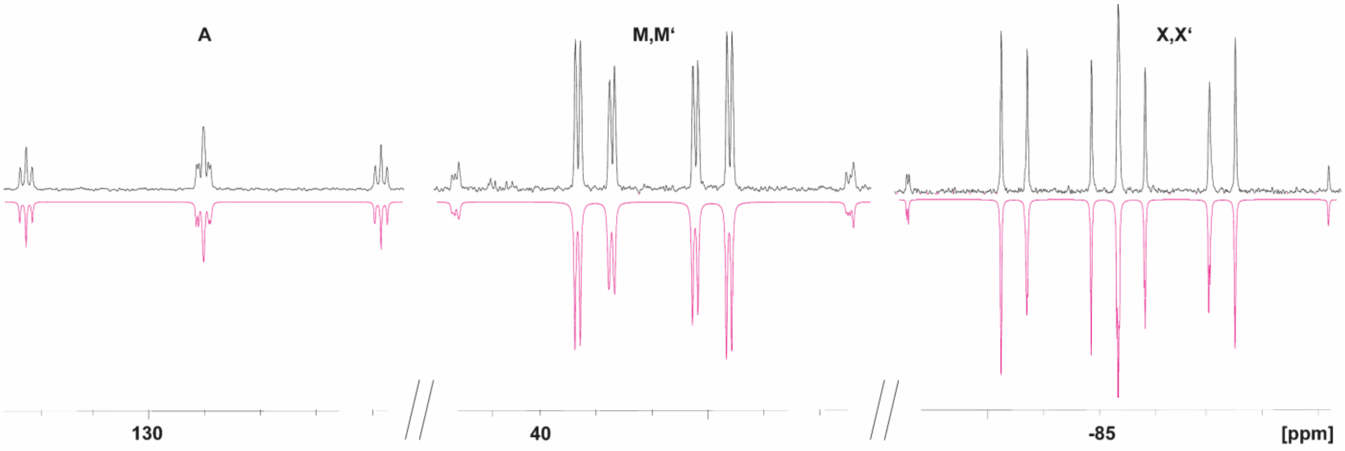

Supplementary Figure 22.
Experimental (top) and simulated (bottom) $^{31}P\{^{1}H\}$ NMR (161.98 MHz, C_6D_6) spectrum of **3b**.

Supplementary Table 6.
Chemical shifts and coupling constants obtained from the simulation (R-factor = 2.83 %) in Supplementary Figure 22.

J [Hz]				δ [ppm]	
$^{1}J_{P_A,P_X}$	372.47	$^{1}J_{P_M,P_X}$	403.22	X, X’	-96.7
$^{1}J_{P_A,P_{X'}}$	372.16	$^{1}J_{P_{M'},P_{X'}}$	404.16		
$^{2}J_{P_A,P_{M'}}$	13.00	$^{2}J_{P_{M'},P_X}$	-35.26	M, M’	37.0
$^{2}J_{P_A,P_M}$	13.00	$^{2}J_{P_M,P_{X'}}$	-37.74		
$^{1}J_{P_M,P_{M'}}$	377.21	$^{2}J_{P_X,P_{X'}}$	1.63	A	129.4

[Cp*Fe(η^4-P_5Me_2)] (**3c**)

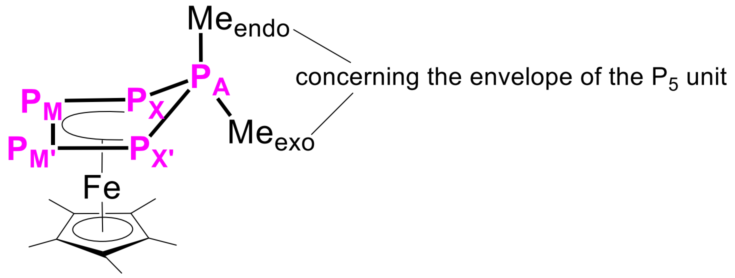

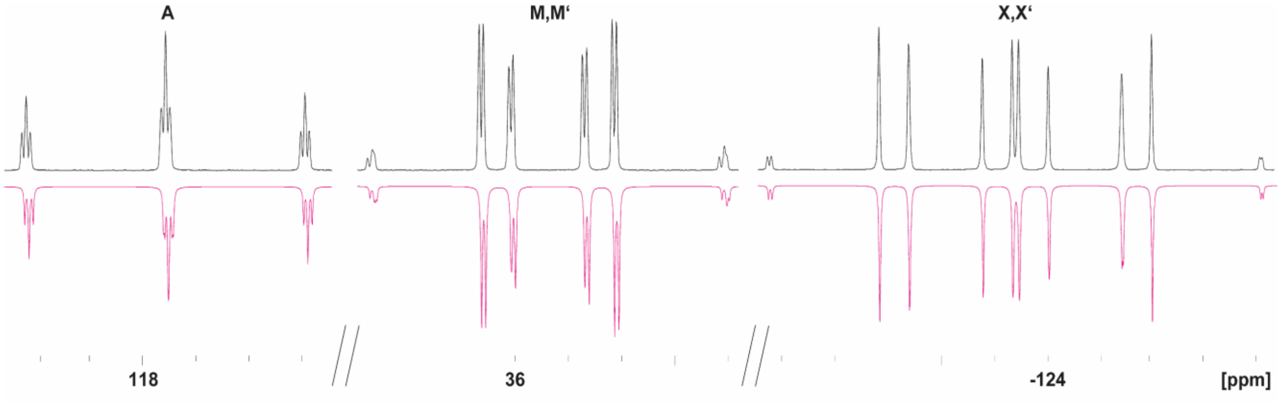

Supplementary Figure 23.

Experimental (top) and simulated (bottom) $^{31}P\{^1H\}$ NMR (161.98 MHz, C_6D_6) spectrum of **3c**.

Supplementary Table 7.

Chemical shifts and coupling constants obtained from the simulation (R-factor = 3.46 %) in Supplementary Figure 23.

J [Hz]				*δ* [ppm]	
$^1J_{P_A,P_X}$	379.70	$^1J_{P_M,P_X}$	404.49	X, X'	-122.7
$^1J_{P_A,P_{X'}}$	380.34	$^1J_{P_{M'},P_{X'}}$	396.47		
$^2J_{P_A,P_{M'}}$	11.47	$^2J_{P_{M'},P_X}$	-32.84	M, M'	35.4
$^2J_{P_A,P_M}$	11.55	$^2J_{P_M,P_{X'}}$	-43.00		
$^1J_{P_M,P_{M'}}$	379.62	$^2J_{P_X,P_{X'}}$	4.89	A	117.2

[Cp*Fe(η^4-P_5Me^iPr)] (**3d**)

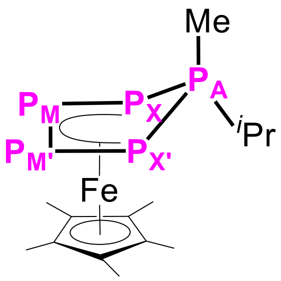

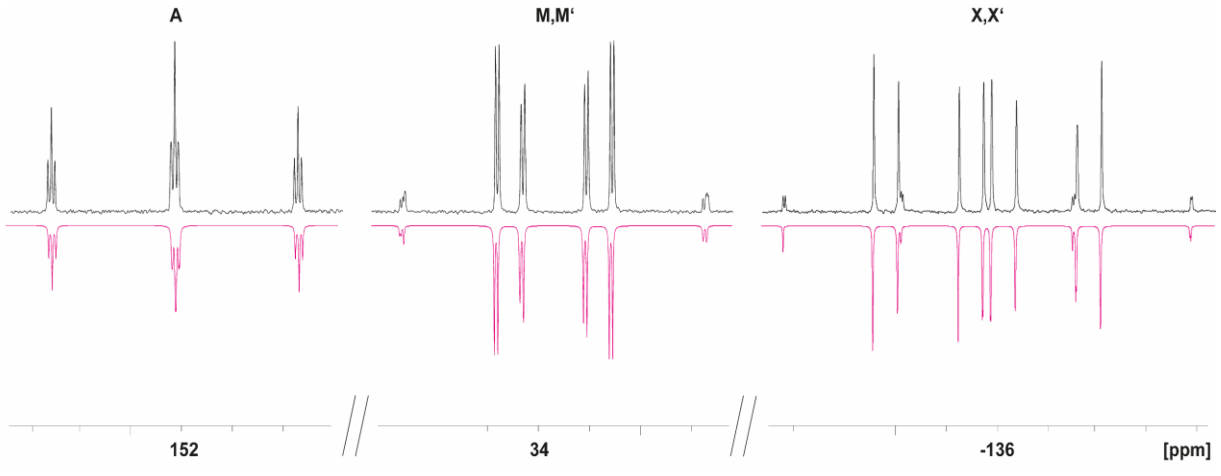

Supplementary Figure 24.
Experimental (top) and simulated (bottom) $^{31}P\{^1H\}$ NMR (161.98 MHz, C_6D_6) spectrum of **3d**.

Supplementary Table 8.
Chemical shifts and coupling constants obtained from the simulation (R-factor = 2.38 %) in Supplementary Figure 24.

J [Hz]				*δ* [ppm]	
$^1J_{P_A,P_X}$	389.45	$^1J_{P_M,P_X}$	404.39	X, X'	-135.8
$^1J_{P_A,P_{X'}}$	388.89	$^1J_{P_{M'},P_{X'}}$	395.77		
$^2J_{P_A,P_{M'}}$	-12.56	$^2J_{P_{M'},P_X}$	-33.85	M, M'	33.3
$^2J_{P_A,P_M}$	-9.93	$^2J_{P_M,P_{X'}}$	-41.00		
$^1J_{P_M,P_{M'}}$	377.57	$^2J_{P_X,P_{X'}}$	-0.60	A	151.8

[Cp*Fe(η4-P5tBuMe)] (**3e**)

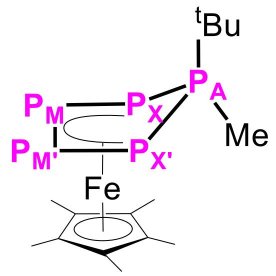

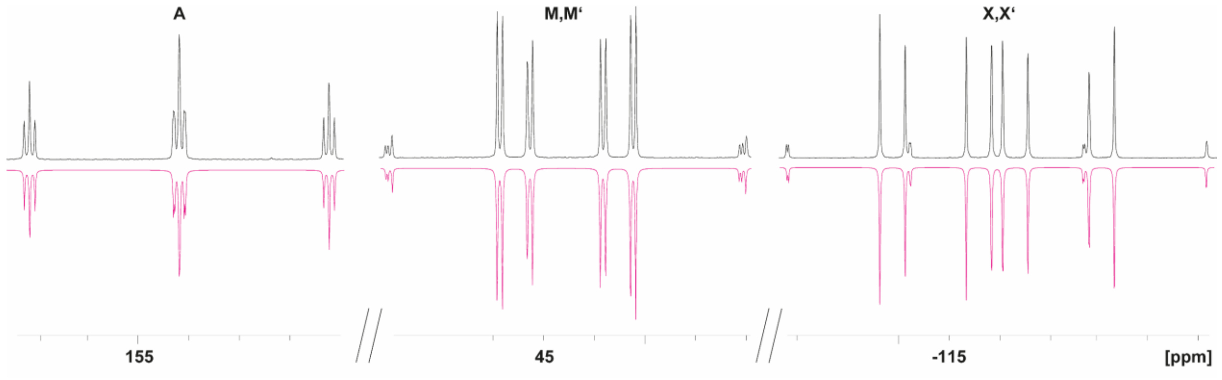

Supplementary Figure 25.
Experimental (top) and simulated (bottom) $^{31}P\{^{1}H\}$ NMR (161.98 MHz, C_6D_6) spectrum of **3e**.

Supplementary Table 9.
Chemical shifts and coupling constants obtained from the simulation (R-factor = 0.95 %) in Supplementary Figure 25.

J [Hz]				δ [ppm]	
$^{1}J_{P_A,P_X}$	405.50	$^{1}J_{P_M,P_X}$	405.50	X, X'	-117.2
$^{1}J_{P_A,P_{X'}}$	404.47	$^{1}J_{P_{M'},P_{X'}}$	404.47		
$^{2}J_{P_A,P_{M'}}$	14.32	$^{2}J_{P_{M'},P_X}$	-39.50	M, M'	47.0
$^{2}J_{P_A,P_M}$	14.32	$^{2}J_{P_M,P_{X'}}$	-39.50		
$^{1}J_{P_M,P_{M'}}$	384.75	$^{2}J_{P_X,P_{X'}}$	4.18	A	151.89

[Cp*Fe(η^4-P_5PhMe)] (**3f**)

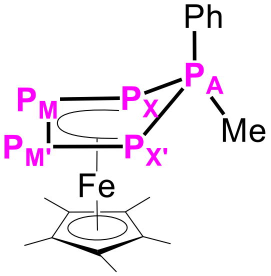

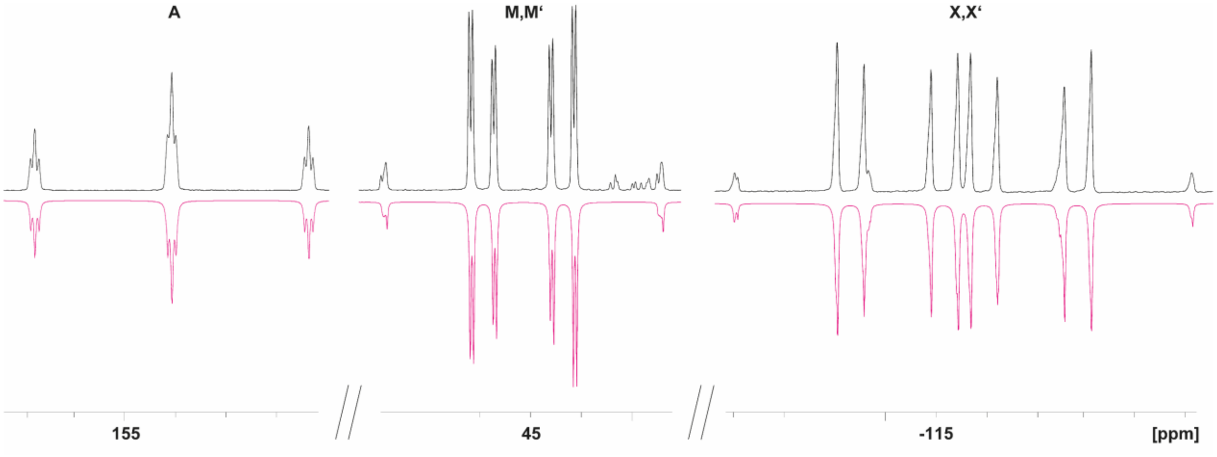

Supplementary Figure 26.

Experimental (top) and simulated (bottom) $^{31}P\{^1H\}$ NMR (161.98 MHz, C_6D_6) spectrum of **3f**.

Supplementary Table 10.

Chemical shifts and coupling constants obtained from the simulation (R-factor = 2.69 %) in Supplementary Figure 26.

J [Hz]				*δ* [ppm]	
$^1J_{P_A,P_X}$	400.22	$^1J_{P_M,P_X}$	414.76	X, X'	-109.5
$^1J_{P_A,P_{X'}}$	400.10	$^1J_{P_{M'},P_{X'}}$	388.69		
$^2J_{P_A,P_{M'}}$	13.53	$^2J_{P_{M'},P_X}$	-26.77	M, M'	40.5
$^2J_{P_A,P_M}$	10.84	$^2J_{P_M,P_{X'}}$	-52.55		
$^1J_{P_M,P_{M'}}$	383.76	$^2J_{P_X,P_{X'}}$	2.39	A	117.7

PMe^{i}PrBnz (**4a**)

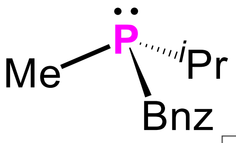

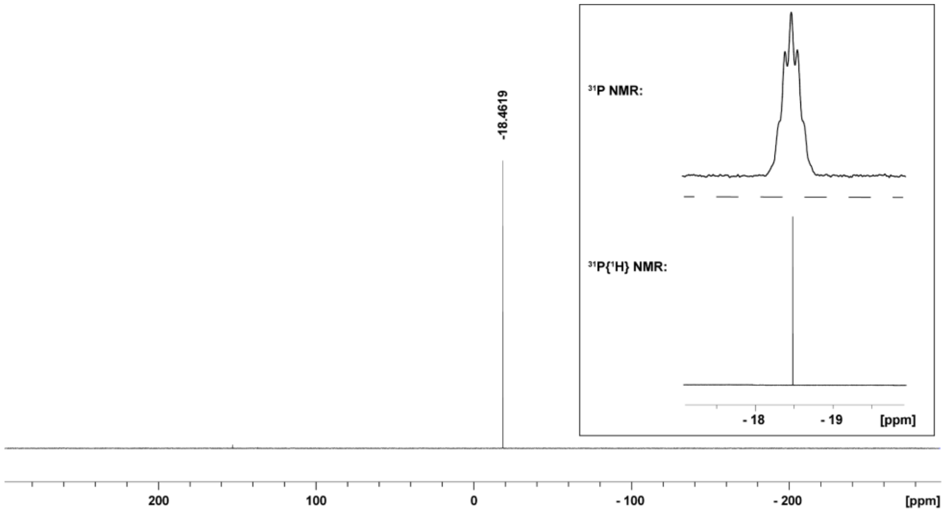

Supplementary Figure 27.
Experimental ^{31}P{^{1}H} NMR (161.98 MHz, C_6D_6) spectrum of **4a**.

P^{t}BuMeBnz (**4b**)

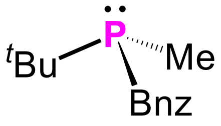

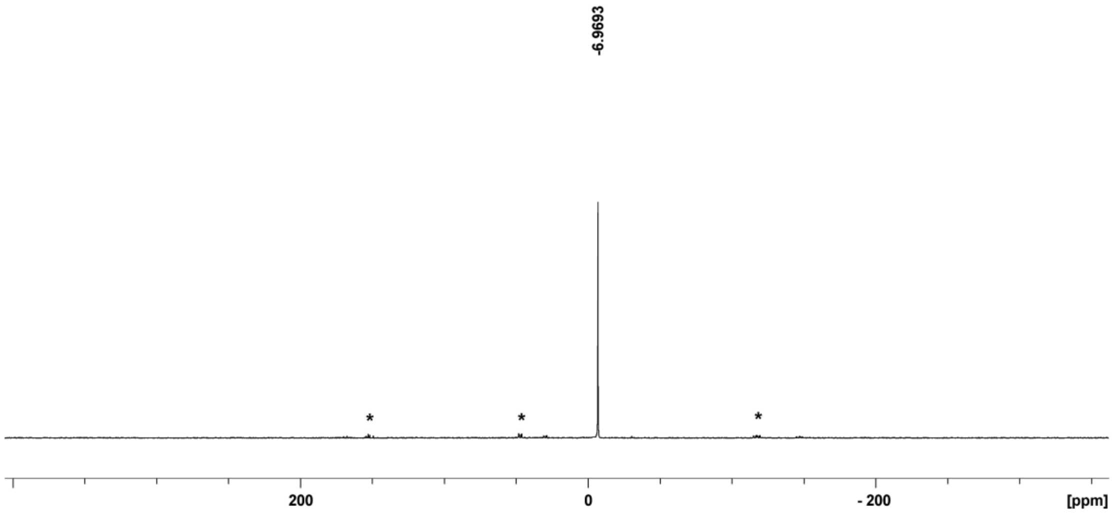

Supplementary Figure 28.
Experimental ^{31}P{^{1}H} NMR (161.98 MHz, C_6D_6) spectrum of **4b** (* = starting material **3e**).

PPhMeBnz (**4c**)

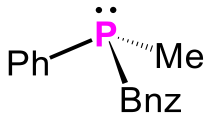

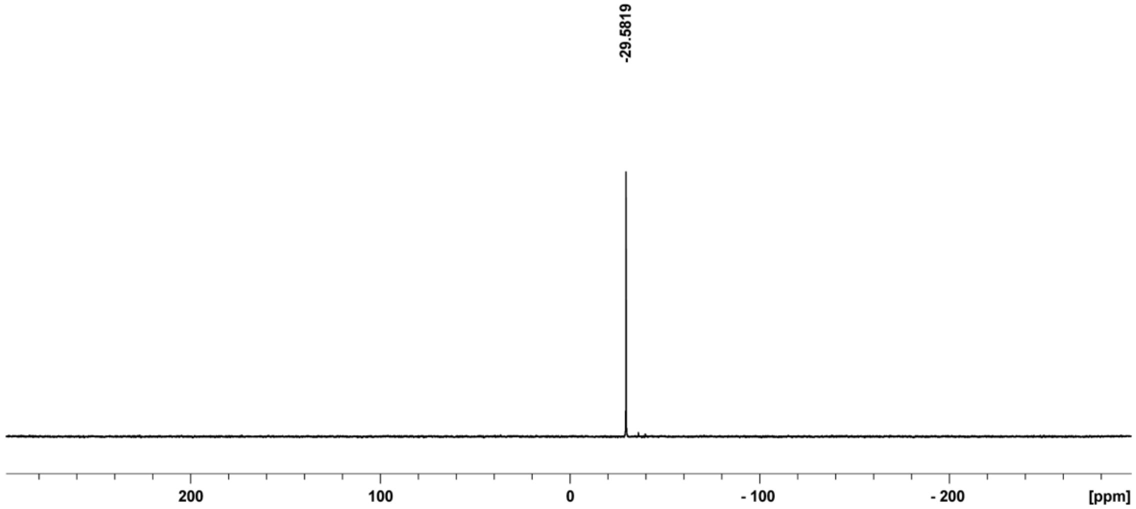

Supplementary Figure 29.
Experimental $^{31}P\{^{1}H\}$ NMR (161.98 MHz, C_6D_6) spectrum of **4c**.

SPMe^{i}PrBnz (**4a’**)

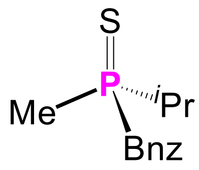

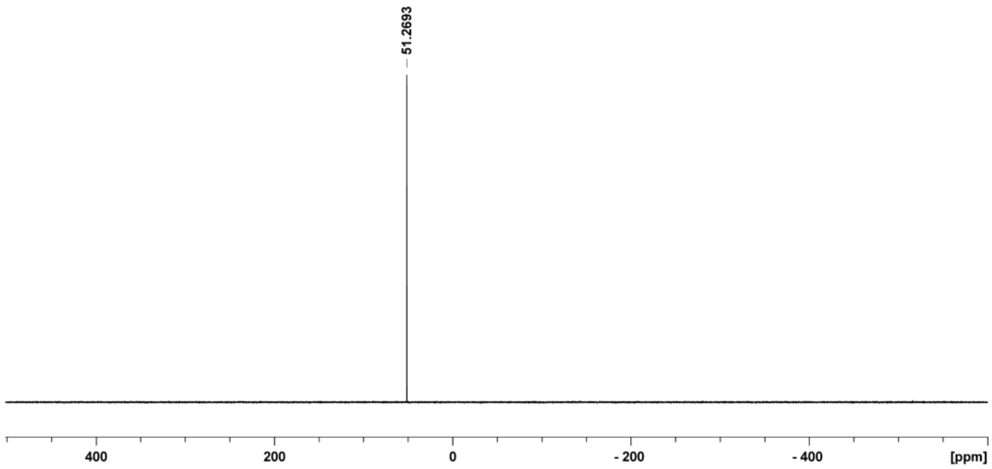

Supplementary Figure 30.
Experimental $^{31}P\{^{1}H\}$ NMR (161.98 MHz, C_6D_6) spectrum of **4a’**.

SP^{t}BuMeBnz (**4b’**)

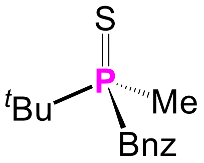

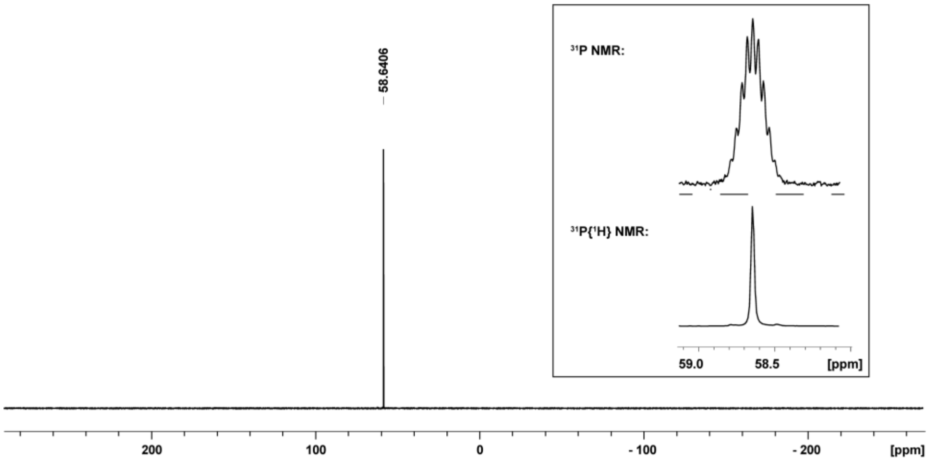

Supplementary Figure 31.
Experimental $^{31}P\{^{1}H\}$ NMR (161.98 MHz, C_6D_6) spectrum of **4b’**.

SPPhMeBnz (**4c’**)

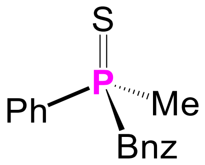

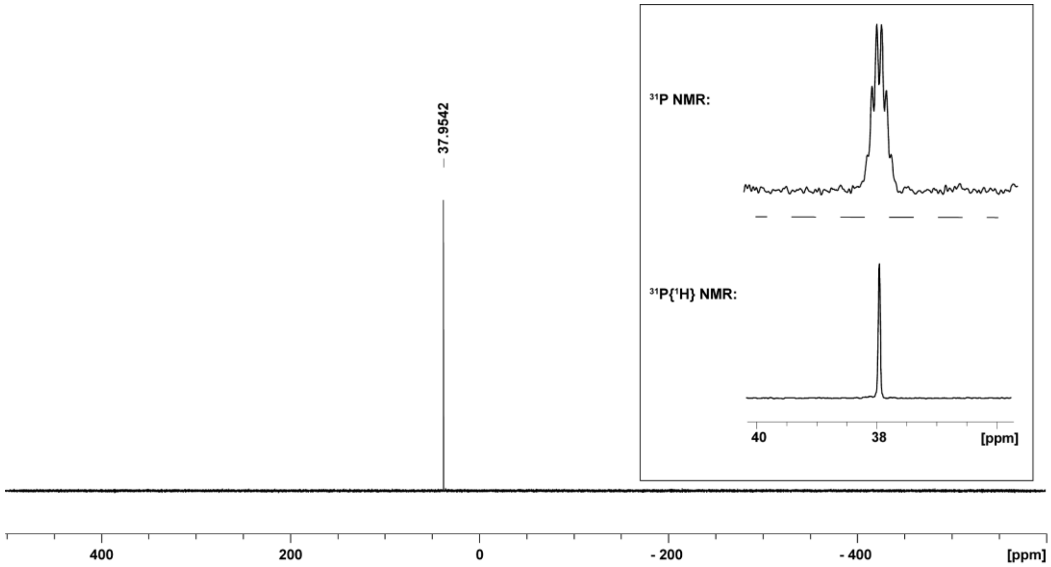

Supplementary Figure 32.
Experimental $^{31}P\{^{1}H\}$ NMR (161.98 MHz, C_6D_6) spectrum of **4c’**.

[K(18c6)(thf)][$Cp^*Fe(\eta^4-P_4)$] (**5**)

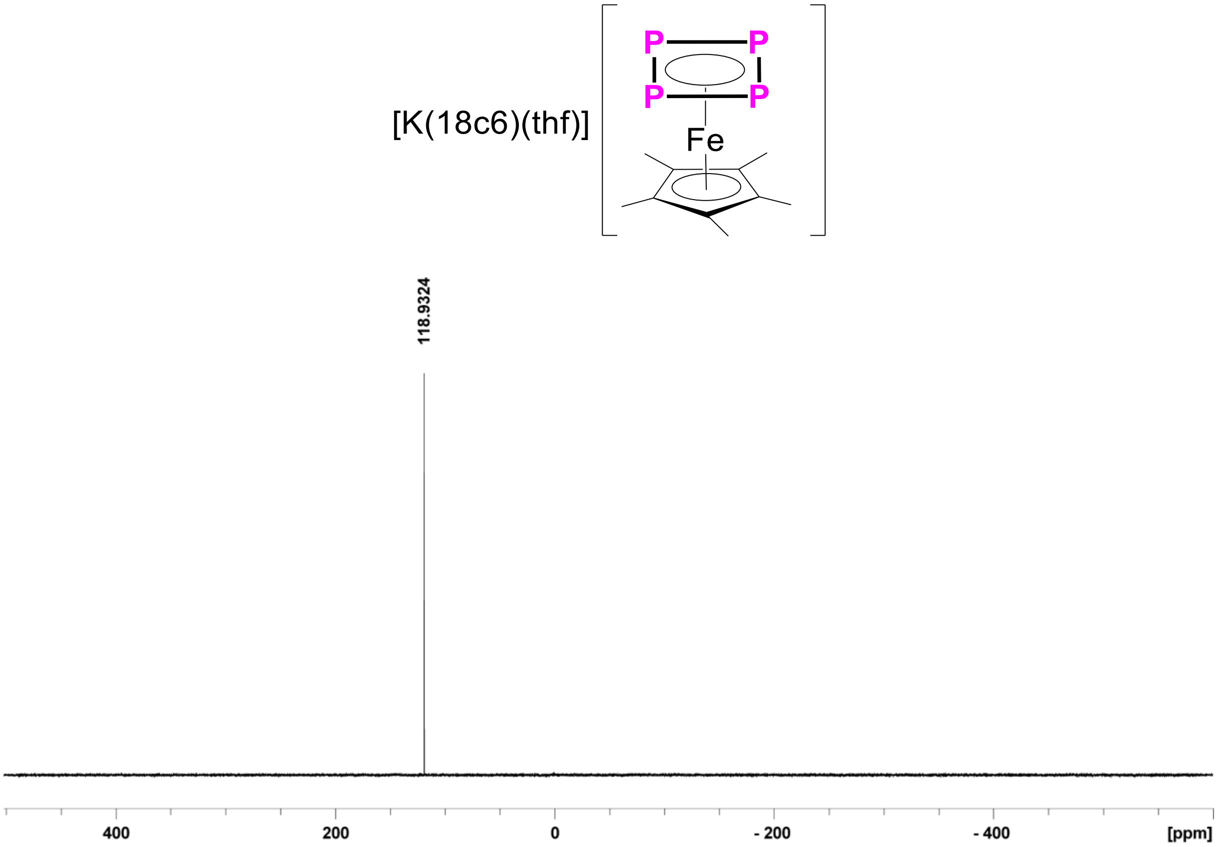

Supplementary Figure 33.
Experimental $^{31}P\{^{1}H\}$ NMR (161.98 MHz, THF-d_8) spectrum of **5.**

^{13}C NMR Spectra:

PMe^{i}PrBnz (**4a**)

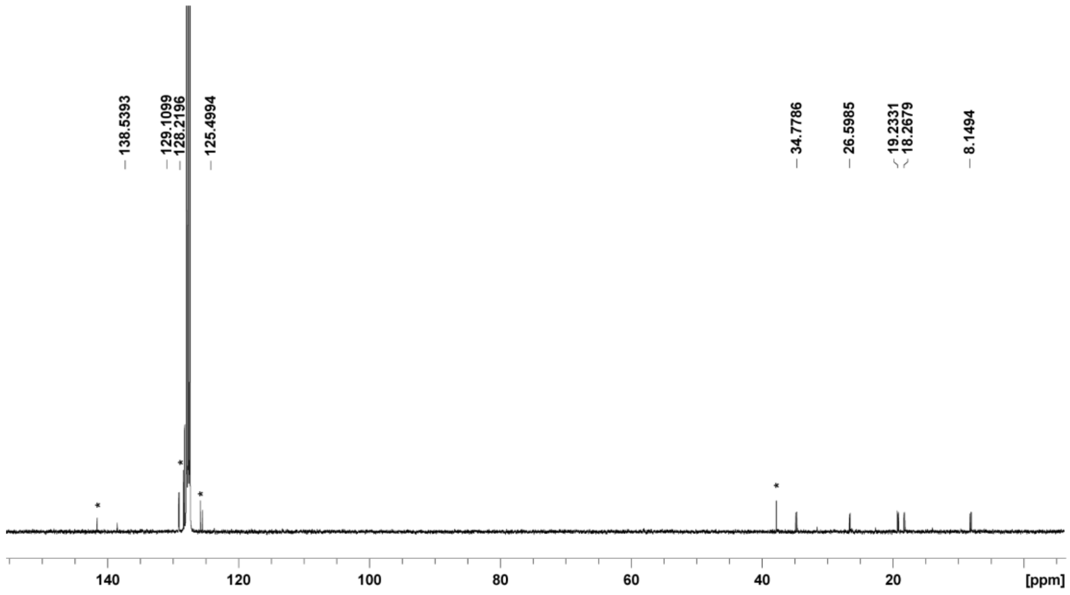

Supplementary Figure 34.

Experimental $^{13}C\{^1H\}$ NMR (100.61 MHz, C_6D_6) spectrum of **4a** (* = 1,2-diphenylethane: decomposition product of KBnz).

P^{t}BuMeBnz (**4b**)

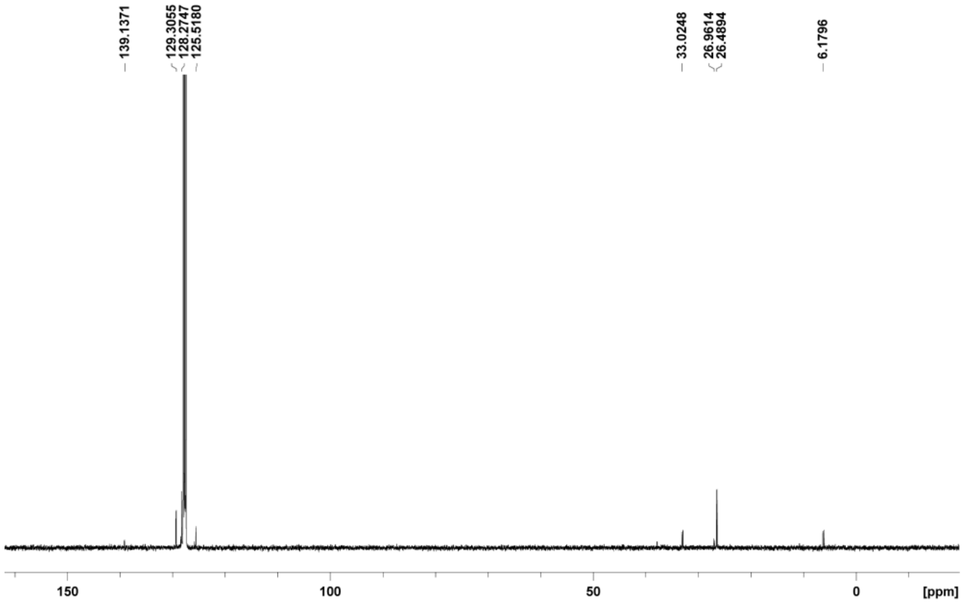

Supplementary Figure 35.

Experimental $^{13}C\{^1H\}$ NMR (100.61 MHz, C_6D_6) spectrum of **4b** (* = starting material **3e**).

PPhMeBnz (**4c**)

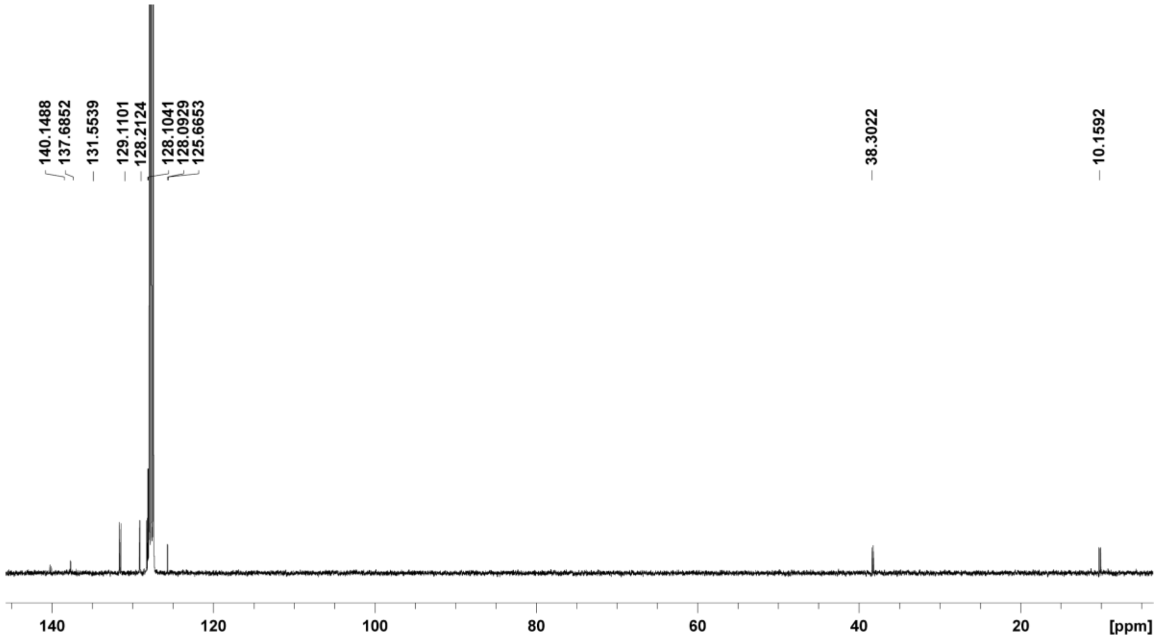

Supplementary Figure 36.
Experimental $^{13}C\{^1H\}$ NMR (100.61 MHz, C_6D_6) spectrum of **4c**.

SPMe^{i}PrBnz (**4a'**)

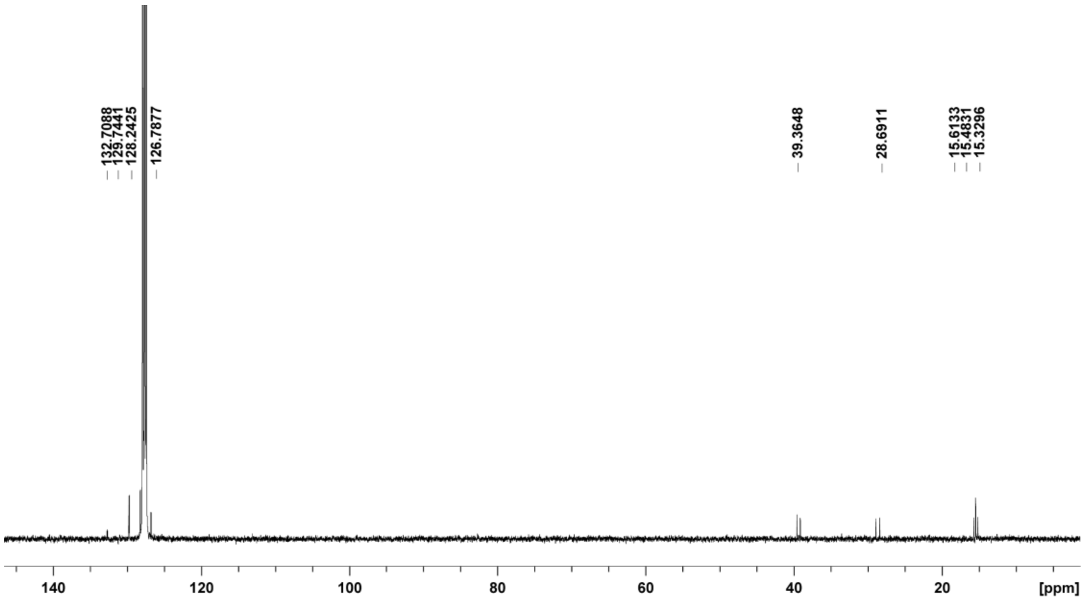

Supplementary Figure 37
Experimental $^{13}C\{^1H\}$ NMR (100.61 MHz, C_6D_6) spectrum of **4a'**.

SP^{t}BuMeBnz (**4b’**)

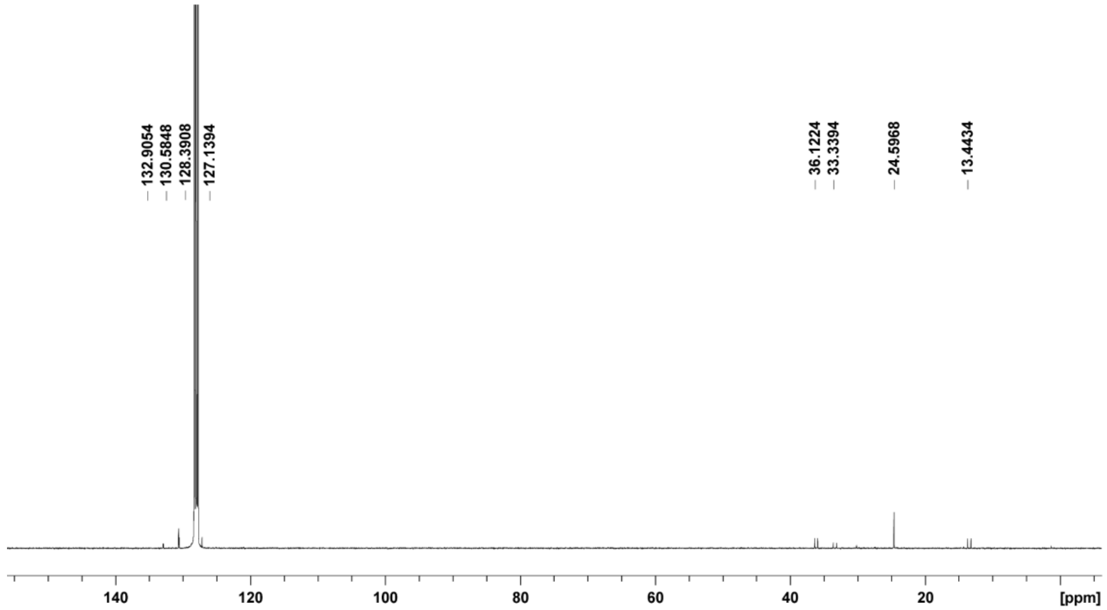

Supplementary Figure 38.
Experimental $^{13}C\{^{1}H\}$ NMR (100.61 MHz, C_6D_6) spectrum of **4b’**.

SPPhMeBnz (**4c’**)

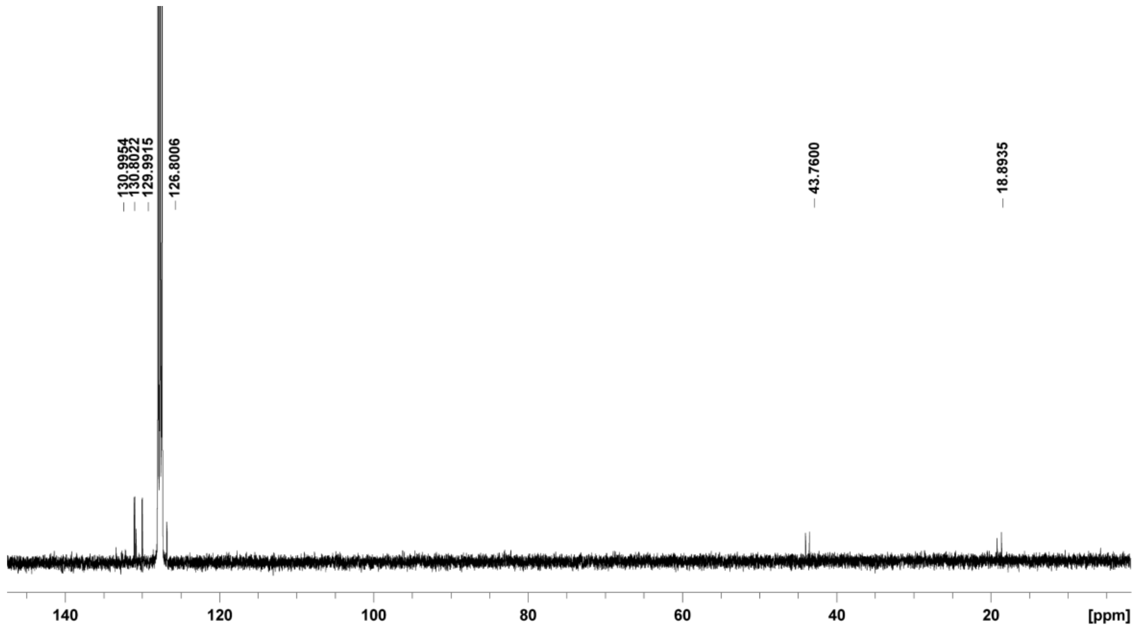

Supplementary Figure 39.
Experimental $^{13}C\{^{1}H\}$ NMR (100.61 MHz, C_6D_6) spectrum of **4c’**.

Crystallographic details

SuiSupplementary Table ingle crystals were selected and measured on a Xcalibur Gemini Ultra diffractometer equipped with an AtlasS2 CCD detector (**2c**, **2d**, **3b**), on a XtaLAB Synergy R DW system equipped with a HyPix-Arc 150 detector (**2e**, **3c**, **3f**, **4c**), on a SuperNova diffractometer equipped with an Atlas CCD detector (**3a**), on a GV50 diffractometer equipped with a TitanS2 CCD detector (**3d**, **3e**, **5**), on a Xcalibur Gemini Ultra diffractometer equipped with a TitanS2 CCD detector (**4a**) or on a SuperNova Dualflex diffractometer equipped with a TitanS2 CCD detector (**4b**). The crystals were kept at T = 123(1) K or 203(2) K (**2c**) during data collection. Data collection and reduction were performed with **CrysAlisPro**. Using **Olex2**, the structures were solved with **ShelXT** and a least-square refinement on F^2 was carried out with **ShelXL**. All non-hydrogen atoms were refined anisotropically unless stated otherwise. Hydrogen atoms at the carbon atoms were located in idealized positions and refined isotropically according to the riding model. Figures were created with **Olex2**.

[Li(dme)$_3$][Cp*Fe(η^4-P$_5$Me)] (2c): The asymmetric unit contains one molecule of **2c**. The P_5 middle deck is disordered over two positions with a distribution of 95:5. To describe the disorder the SADI and SIMU restrains were applied. Further, is one of the DME molecules, which are coordinated to the lithium ion, disordered over two positions with an occupancy refined to 67:33. To describe this disorder the SIMU restrain was applied. The structure in solid state is given in Supplementary Figure 40 and S41. Crystallographic and refinement data are summarized in Supplementary Table 11.

[Li(12c4)$_2$][Cp*Fe(η^4-P$_5{}^t$Bu)] (2d): The asymmetric unit contains one molecule of **2d** without any disorder. The structure in solid state is given in Supplementary Figure 42. Crystallographic and refinement data are summarized in Supplementary Table 11.

[Li(12c4)(thf)][Cp*Fe(η^4-P$_5$Ph)] (2e): The asymmetric unit contains one molecule of **2e** without any disorder. Compound **2e** crystalizes in the acentric spacegroup *Pn* with a flack parameter of -0.004(3). The structure in solid state is given in Supplementary Figure 43. Crystallographic and refinement data are summarized in Supplementary Table 11.

[Cp*Fe{η^4-P$_5$(NMe$_2$)Me}] (3a): The asymmetric unit contains two molecules of **3a** without any disorder. The structure in solid state is given in Supplementary Figure 44. Crystallographic and refinement data are summarized in Supplementary Table 11.

[Cp*Fe{η^4-P$_5$(CH$_2$SiMe$_3$)Me}] (3b): The asymmetric unit contains one molecule of **3b**. The Cp* ligand and the P_5 middle deck with the methyl substituent and the CH_2SiMe_3 substituent are disordered over two positions with a distribution of approximately 66:34. To describe these disorders the restraints SADI and SIMU were applied. The structure in solid state is given in Supplementary Figure 45 and S46. Crystallographic and refinement data are summarized in Supplementary Table 11.

[Cp*Fe(η^4-P$_5$Me$_2$)] (3c): The asymmetric unit contains three molecules of **3c**. One of them without any disorder, its structure in solid state is given in Supplementary Figure 47.

The other two show disorder: The P_5 middle deck is disordered over two positions with a distribution of 83:17 / 91:9, respectively. To describe the disorder the SADI and SIMU restrains were applied. Crystallographic and refinement data are summarized in Supplementary Table 12.

[Cp*Fe(η^4-P_5Me^{i}Pr)] (3d): The asymmetric unit contains one molecule of **3d** without any disorder. Since the measured crystal was twinned, a HKLF 5 refinement was applied (twin law: -0.9993 0.0012 0.0018 0.0018 -0.9987 0.0041 0.0931 0.0377 1.0006; BASF 0.263(2)). The structure in solid state is given in Supplementary Figure 48. Crystallographic and refinement data are summarized in Supplementary Table 12.

[Cp*Fe(η^4-P_5^{t}BuMe)] (3e): The asymmetric unit contains half a molecule of **3d** without any disorder. Compound **3d** crystalizes in the acentric space group *Im* with a flack parameter of 0.000(3). The structure in solid state is given in Supplementary Figure 49. Crystallographic and refinement data are summarized in Supplementary Table 12.

[Cp*Fe(η^4-P_5PhMe)] (3f): The asymmetric unit contains two molecules of **3f** without any disorder. The structure in solid state of one molecule is depicted in Supplementary Figure 50. Crystallographic and refinement data are summarized in Supplementary Table 12.

SPMe^{i}PrBnz (**4a')**: The asymmetric unit contains one molecule of **4a'**. The P(S)Me^{i}Pr unit is disordered over two positions with a distribution of 93:7. Due to the low occupancy of the second part, were only the sulphur and the phosphorus atoms anisotropically refined. The structure in solid state is given in Supplementary Figure 51 and S52. Crystallographic and refinement data are summarized in Supplementary Table 12.

SP^{t}BuMeBnz (**4b')**: The asymmetric unit contains one molecule of **4b'** without any disorder. Since the measured crystal was twinned, a HKLF 5 refinement was applied (twin law: -0.5569 0.0005 0.4439 -0.0003 -1.0008 -0.0001 1.5524 -0.0002 0.5587; BASF 0.1610(7)) The structure in solid state is given in Supplementary Figure 53. Crystallographic and refinement data are summarized in Supplementary Table 13.

SPPhMeBnz (**4c')**: The asymmetric unit contains one molecule of **4c'** without any disorder. The structure in solid state of one molecule is depicted in Supplementary Figure 54. Crystallographic and refinement data are summarized in Supplementary Table 13.

[K(18c6)(thf)][Cp*Fe(η^4-P_4)] (5): The asymmetric unit contains half a molecule of **5**. The symmetry of the molecule is lower than the site symmetry (mirror plane), which causes the disorder of the Cp* ligand and one THF molecule over two positions. The complete structure in solid state is given in Supplementary Figure 55. Crystallographic and refinement data are summarized in Supplementary Table 13.

The X-ray crystallographic coordinates for structures reported in this study have been deposited at the Cambridge Crystallographic Data Centre (CCDC), under deposition numbers CCDC-2041977 (**2c**), CCDC-2041978 (**2d**), CCDC-2083624 (**2e**), CCDC-2041979 (**3a**), CCDC-2041980 (**3b**), CCDC-2083625 (**3c**), CCDC-2083626 (**3d**), CCDC-

2041981 (**3e**), CCDC-2083627 (**3f**), CCDC-2083628 (**4a’**), CCDC-2083629 (**4b’**), CCDC-2083630(**4c’**) and CCDC-2083631 (**5**). These data can be obtained free of charge from The Cambridge Crystallographic Data Centre via www.ccdc.cam.ac.uk/data_request/cif.

Supplementary Table 11.

Crystallographic details of **2c**, **2d**, **2e**, **3a** and **3b**.

Compound	2c	2d	2e	3a	3b
CCDC	2041977	2041978	2041979	2041979	2041980
Formula	$C_{23}H_{48}FeLiO_6P_5$	$C_{30}H_{56}FeLiO_8P_5$	$C_{28}H_{44}FeLiO_5P_5$	$C_{13}H_{24}FeNP_5$	$C_{15}H_{29}FeP_5Si$
$D_{calc.}$/ g cm^{-3}	1.235	1.326	1.393	1.433	1.353
μ/mm^{-1}	5.980	5.507	6.366	10.388	9.407
Formula Weight	638.25	762.38	678.27	405.03	448.17
Colour	black	dark green	dark green	black	black
Shape	truncated prism	block	block-shaped	prism	plate
Size/mm^3	0.40×0.15×0.10	0.46×0.30×0.12	0.27×0.17×0.13	0.18×0.12×0.08	0.34×0.25×0.04
T/K	203(2)	123.35(10)	123.01(10)	123(2)	122.9(5)
Crystal System	monoclinic	monoclinic	monoclinic	monoclinic	monoclinic
Flack Parameter	/	/	-0.004(3)	/	/
Hooft Parameter	/	/	-0.0039(8)	/	/
Space Group	$P2_1/c$	$P2_1/n$	*Pn*	$P2_1/c$	$P2_1/n$
a/Å	15.7568(2)	9.9189(3)	9.30050(10)	14.3059(2)	8.32648(12)
b/Å	13.1533(2)	19.7484(5)	9.66730(10)	29.8376(4)	9.12374(11)
c/Å	16.9675(2)	19.5371(7)	18.5722(2)	8.86570(10)	29.0199(4)
α/°	90	90	90	90	90
β/°	102.5080(10)	93.863(3)	104.4500(10)	97.1750(10)	93.6816(12)
γ/°	90	90	90	90	90
V/Å^3	3433.12(8)	3818.3(2)	1617.02(3)	3754.72(8)	2200.05(5)
Z	4	4	2	8	4
Z'	1	1	1	2	1
Wavelength/Å	1.54178	1.54184	1.54184	1.54178	1.54184
Radiation type	CuK_α	CuK_α	CuK_α	CuK_α	CuK_α
θ_{min}/°	4.292	4.478	4.574	4.299	5.083
θ_{max}/°	66.704	73.095	74.468	74.224	66.767
Measured Refl's.	25941	14979	26196	19702	25018
Ind't Refl's	6042	7468	5149	7371	3882
Refl's with I > 2 σ(I)	4741	5663	5093	6535	3568
R_{int}	0.0273	0.0404	0.0183	0.0168	0.0485
Parameters	439	414	366	377	389
Restraints	82	0	2	0	139
Largest Peak	0.367	0.570	0.411	0.252	0.693
Deepest Hole	-0.221	-0.379	-0.179	-0.322	-0.547
GooF	0.966	1.018	1.084	1.013	1.083
wR_2 (all data)	0.0754	0.1329	0.0694	0.0549	0.1134
wR_2	0.0738	0.1199	0.0692	0.0543	0.1093
R_1 (all data)	0.0372	0.0737	0.0256	0.0238	0.0438
R_1	0.0290	0.0510	0.0253	0.0208	0.0400

Supplementary Table 12.

Crystallographic details of **3c**, **3e**, **3e**, **3f** and **4a'**.

Compound	3c	3d	3e	3f	4a'
CCDC	2083625	2083626	2041981	2083627	2083628
Formula	$C_{12}H_{21}FeP_5$	$C_{14}H_{25}FeP_5$	$C_{15}H_{27}FeP_5$	$C_{17}H_{23}FeP_5$	$C_{11}H_{17}PS$
$D_{calc.}$/ g cm^{-3}	1.471	1.401	1.382	1.456	1.210
μ/mm^{-1}	11.427	10.168	9.711	9.800	3.382
Formula Weight	375.99	404.04	418.06	438.05	212.27
Colour	dark green	dark red	metallic dark green	dark green	clear colourless
Shape	block	block	block	plate-shaped	needle-shaped
Size/mm^3	0.20×0.10×0.07	0.20×0.08×0.07	0.23×0.11×0.06	0.20×0.12×0.10	0.21×0.07×0.04
T/K	123.00(10)	123.01(11)	123.01(10)	123.01(10)	123.00(10)
Crystal System	monoclinic	monoclinic	monoclinic	monoclinic	monoclinic
Flack Parameter	/	/	0.000(2)		
Hooft Parameter	/	/	0.0067(13)		
Space Group	$P2_1/c$	$P2_1/n$	*Cm*	$P2_1/c$	$P2_1/c$
a/Å	19.5498(3)	7.8177(3)	7.86145(10)	14.33040(10)	11.6109(14)
b/Å	9.20470(10)	9.1917(4)	12.31599(13)	31.3940(3)	6.2158(7)
c/Å	28.3406(4)	26.6502(9)	10.48456(11)	8.90470(10)	16.623(2)
α/°	90	90	90	90	90
β/°	93.0600(10)	90.614(3)	98.3685(10)	94.1640(10)	103.810(13)
γ/°	90	90	90	90	90
V/Å^3	5092.62(12)	1914.92(13)	1003.86(4)	3995.55(7)	1165.0(3)
Z	12	4	2	8	4
Z'	3	1	0.5	2	1
Wavelength/Å	1.54184	1.54184	1.54184	1.54184	1.54184
Radiation type	CuK$_\alpha$	CuK$_\alpha$	CuK$_\alpha$	CuK$_\alpha$	CuK$_\alpha$
θ_{min}/°	2.263	3.317	5.576	2.815	3.921
θ_{max}/°	75.411	74.131	74.027	74.510	66.762
Measured Refl's.	39361	17620	10956	32976	5824
Ind't Refl's	10304	5931	1997	8063	2042
Refl's with I > 2 σ(I)	8981	5184	1978	7219	1679
R_{int}	0.0609	0.1457	0.0359	0.0257	0.0466
Parameters	601	190	110	426	154
Restraints	127	0	26	0	0
Largest Peak	0.999	0.850	0.268	1.105	0.250
Deepest Hole	-0.767	-0.584	-0.229	-0.739	-0.389
GooF	1.054	1.070	1.057	1.035	1.045
wR_2 (all data)	0.1527	0.2104	0.0539	0.1023	0.1109
wR_2	0.1463	0.2002	0.0538	0.0986	0.1042
R_1 (all data)	0.0601	0.0714	0.0204	0.0458	0.0543
R_1	0.0537	0.0667	0.0203	0.0402	0.0432

Supplementary Table 13.

Crystallographic details of **4b'**, **4c'** and **5.**

**Compound**	**4b'**	**4c'**	**5**
CCDC	2083629	2083630	2083631
Formula	$C_{12}H_{19}PS$	$C_{14}H_{15}PS$	$C_{26}H_{47}FeKO_7P_4$
$D_{calc.}$/ g cm^{-3}	1.170	1.291	1.352
μ/mm^{-1}	3.098	3.193	6.773
Formula Weight	226.30	246.29	690.46
Colour	colourless	colourless	light green
Shape	block	needle-shaped	plate
Size/mm^3	0.20×0.18×0.14	0.24×0.03×0.02	0.14×0.13×0.02
T/K	123.00(10)	123.01(10)	122.97(12)
Crystal System	monoclinic	triclinic	orthorhombic
Flack Parameter	/		/
Hooft Parameter	/		/
Space Group	$P2_1/n$	P-1	$Pnnm$
a/Å	11.3685(4)	6.2144(2)	10.9945(2)
b/Å	6.3729(2)	9.3867(3)	25.8230(4)
c/Å	18.2100(6)	11.7876(5)	11.9441(2)
α/°	90	108.326(3)	90
β/°	103.385(4)	98.328(3)	90
γ/°	90	97.786(3)	90
V/Å^3	1283.48(8)	633.74(4)	3391.06(10)
Z	4	2	4
Z'	1	1	0.5
Wavelength/Å	1.54184	1.54184	1.54184
Radiation type	CuK_α	CuK_α	CuK_α
θ_{min}/°	4.194	4.032	3.423
θ_{max}/°	66.947	74.326	73.841
Measured Refl's.	19609	8650	19093
Ind't Refl's	4201	2532	3563
Refl's with I > 2 σ(I)	3679	2206	3277
R_{int}	0.0525	0.0260	0.0459
Parameters	132	146	258
Restraints	0	0	34
Largest Peak	0.359	0.471	0.543
Deepest Hole	-0.366	-0.413	-0.429
GooF	1.041	1.049	1.150
wR_2 (all data)	0.1067	0.0990	0.1190
wR_2	0.1044	0.0936	0.1170
R_1 (all data)	0.0405	0.0431	0.0573
R_1	0.0362	0.0364	0.0531

[Li(dme)3][Cp*Fe(η4-P5Me)] (**2c)**

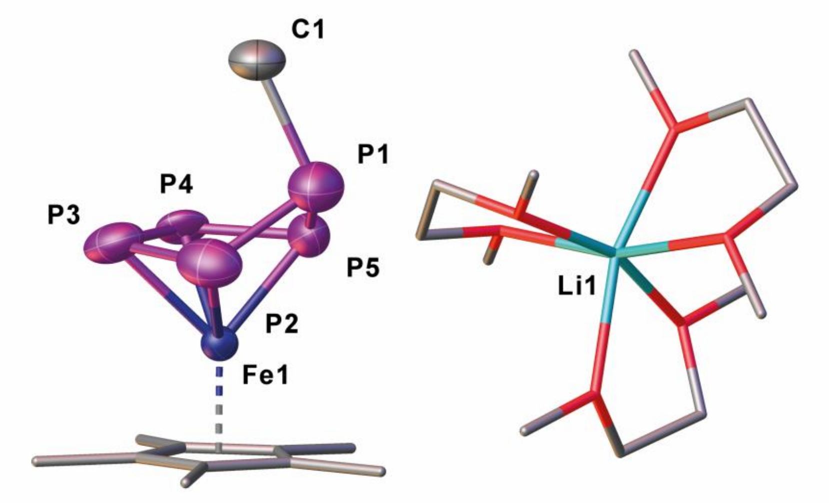

Supplementary Figure 40.

Molecular structure of **2c** with thermal ellipsoids at 50% probability level. The hydrogen atoms and disordered solvents molecules are omitted for clarity. The Cp* ligand and the cation $[Li(dme)_3]^+$ are drawn in the wire frame model.

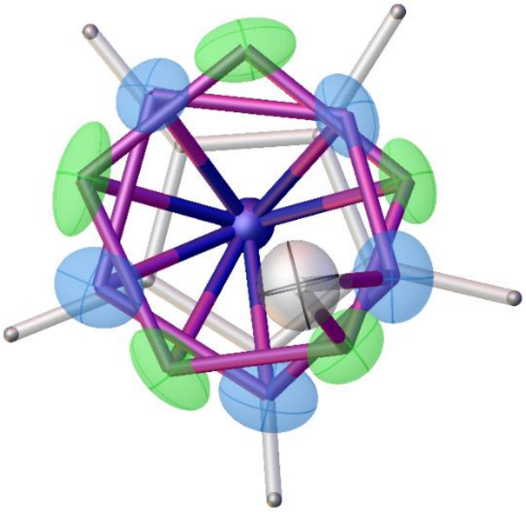

Supplementary Figure 41.

Top view of the molecular structure of **2c** with thermal ellipsoids at 50 % probability level. The disordered P_5 middle deck is highlighted blue (Part 1) and green (Part 2). The Cp* ligands are drawn in the wire frame model.

Supplementary Table 14.
Selected bond length of **2c**.

Atom-Atom	Length [Å]
**Part 1**	
P1–P2	2.050(14)
P2–P3	2.048(14)
P3–P4	2.041(13)
P4–P5	1.993(13)
P1–P5	2.009(14)
P1–C1	2.081(15)
**Part 2**	
P1A–P2A	2.1576(10)
P2A–P3A	2.1458(14)
P3A–P4A	2.1277(15)
P4A–P5A	2.1397(12)
P1A–P5A	2.1521(10)
P1A–C1	1.849(2)

Atom-Atom	Length [Å]
**Part 1**	
Fe1–P2	2.321(13)
Fe1–P3	2.217(12)
Fe1–P4	2.185(9)
Fe1–P5	2.146(11)
Fe1–P1	3.028(14)
**Part 2**	
Fe1–P2A	2.3056(7)
Fe1–P3A	2.3166(7)
Fe1–P4A	2.3357(7)
Fe1–P5A	2.3222(6)
Fe1–P1A	3.1817(7)

Supplementary Table 15.
Selected angles of **2c**.

Atom-Atom-Atom	Angle [°]
**Part 1**	
P1–P2–P3	102.8(8)
P2–P3–P4	106.8(7)
P4–P5–P1	110.4(7)
P2–P1–C1	98.2(7)
**Part 2**	
P1A–P2A–P3A	105.73(4)
P2A–P3A–P4A	103.51(4)
P4A–P5A–P1A	106.53(4)

Atom-Atom-Atom	Angle [°]
**Part 1**	
P3–P4–P5	99.4(7)
P5–P1–P2	93.6(7)
P5–P1–C1	113.0(7)
**Part 2**	
P3A–P4A–P5A	102.79(4)
P5A–P1A–P2A	92.11(3)
P5A–P1A–C1	107.66(9)
P2A–P1A–C1	108.17(9)

[Li(12c4)$_2$][Cp*Fe(η^4-P$_5$^{t}Bu)] (**2d**)

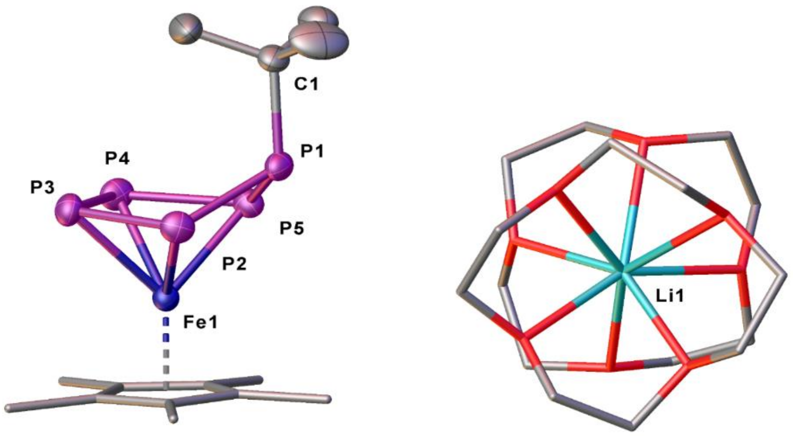

Supplementary Figure 42.
Molecular structure of **2d** with thermal ellipsoids at 50% probability level. The hydrogen atoms are omitted for clarity. The Cp* ligand and the cation [Li(12c4)$_2$]$^+$ are drawn in the wire frame model.

Supplementary Table 16.
Selected bond length of **2d**.

Atom-Atom	Length [Å]	Atom-Atom	Length [Å]
P1–P2	2.1564(12)	Fe1–P2	2.3149(9)
P2–P3	2.1552(12)	Fe1–P3	2.3234(10)
P3–P4	2.1279(12)	Fe1–P4	2.3191(10)
P4–P5	2.1590(12)	Fe1–P5	2.3225(10)
P1–P5	2.1510(11)	Fe1–P1	3.1302(10)
P1–C1	1.898(3)		

Supplementary Table 17.
Selected angles of **2d**.

Atom-Atom-Atom	Angle [°]	Atom-Atom-Atom	Angle [°]
P1–P2–P3	108.99(5)	P3–P4–P5	103.22(5)
P2–P3–P4	103.59(5)	P5–P1–P2	93.14(4)
P4–P5–P1	109.45(5)	P5–P1–C1	113.40(12)
P2–P1–C1	113.91(11)		

[Li(12c4)(thf)][Cp*Fe(η^4-P_5Ph)] (**2e**)

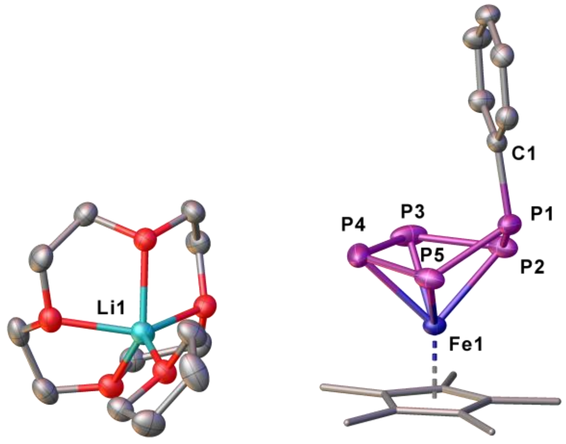

Supplementary Figure 43.

Molecular structure of **2e** with thermal ellipsoids at 50% probability level. The hydrogen atoms are omitted for clarity. The Cp* ligand is drawn in the wire frame model.

Supplementary Table 18.

Selected bond length of **2e**.

Atom-Atom	Length [Å]
P1–P2	2.1554(12)
P2–P3	2.1474(15)
P3–P4	2.1300(16)

Atom-Atom	Length [Å]
P4–P5	2.1463(14)
P1–P5	2.1671(11)
P1–C1	1.841(3)

Supplementary Table 19.

Selected angles of **2e**.

Atom-Atom-Atom	Angle [°]
P1–P2–P3	107.94(5)
P2–P3–P4	103.55(5)
P4–P5–P1	107.37(5)
P2–P1–C1	111.35(11)

Atom-Atom-Atom	Angle [°]
P3–P4–P5	103.27(5)
P5–P1–P2	92.65(4)
P5–P1–C1	111.92(9)

[Cp*Fe(η^4-P_5NMe_2Me)] (**3a**)

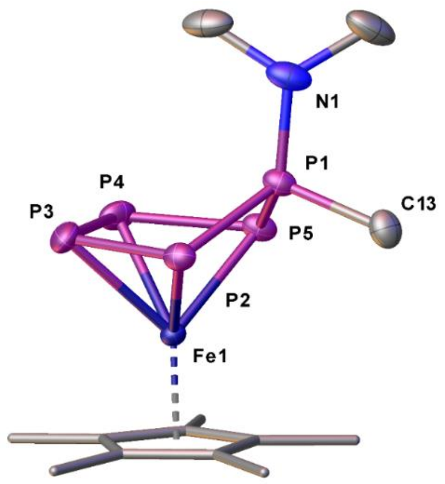

Supplementary Figure 44.

Molecular structure of **3a** with thermal ellipsoids at 50% probability level. The hydrogen atoms are omitted for clarity. The Cp* ligand is drawn in the wire frame model.

Supplementary Table 20.

Selected bond length of **3a**

Atom-Atom	Length [Å]
P1–P2	2.1347(5)
P2–P3	2.1342(5)
P3–P4	2.1519(6)
P4–P5	2.1360(5)
P1–P5	2.1426(5)
P1–N1	1.6779(12)

Atom-Atom	Length [Å]
Fe1–P2	2.3303(4)
Fe1–P3	2.3198(4)
Fe1–P4	2.3399(4)
Fe1–P5	2.3168(4)
Fe1–P1	2.9843(7)
P1–C13	1.8168(14)

Supplementary Table 21.

Selected angles of **3a**.

Atom-Atom-Atom	Angle [°]
P1–P2–P3	102.06(2)
P2–P3–P4	105.44(2)
P4–P5–P1	101.54(2)
P2–P1–N1	117.53(5)
P5–P1–N1	122.92(5)

Atom-Atom-Atom	Angle [°]
P3–P4–P5	105.14(2)
P5–P1–P2	100.084(19)
N1-P1-C13	103.77(7)
P2–P1–C13	104.76(5)
P5–P1–C13	106.16(5)

[Cp*Fe{η[4]-$P_5(CH_2SiMe_3)Me$}] (**3b**)

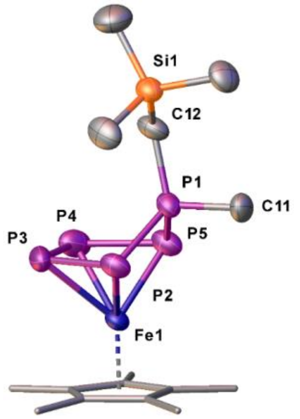

Supplementary Figure 45.
Molecular structure of **3b** with thermal ellipsoids at 50% probability level. The hydrogen atoms are omitted for clarity. The Cp* ligands are drawn in the wire frame model.

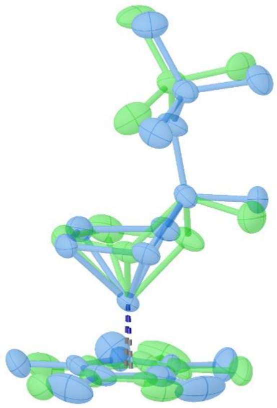

Supplementary Figure 46.
Side view of the molecular structure of **3b** with thermal ellipsoids at 50 % probability level. The disorder is highlighted blue (Part 1) and green (Part 2). The hydrogen atoms are omitted for clarity.

Supplementary Table 22.
Selected bond length of **3b**.

Atom-Atom	Length [Å]	Atom-Atom	Length [Å]
**Part 1**		**Part 1**	
P1–P2	2.089(3)	Fe1–P2	2.376(3)
P2–P3	2.132(3)	Fe1–P3	2.3679(15)
P3–P4	2.135(3)	Fe1–P4	2.3250(15)
P4–P5	2.142(3)	Fe1–P5	2.288(2)
P1–P5	2.164(2)	Fe1–P1	3.0848(8)
P1–C11	1.785(5)	P1–C12	1.796(3)

Part 2	
P1–P2A	2.230(6)
P2A–P3A	2.128(5)
P3A–P4A	2.130(5)
P4A–P5A	2.131(5)
P1–P5A	2.108(4)
P1–C11A	1.922(11)

Part 2	
Fe1–P2A	2.189(6)
Fe1–P3A	2.272(3)
Fe1–P4A	2.373(3)
Fe1–P5A	2.352(3)
Fe1–P1	3.0848(8)
P1–C12	1.796(3)

Supplementary Table 23.

Selected angles of **3b**.

Atom-Atom-Atom	Angle [°]
**Part 1**	
P1–P2–P3	98.85(13)
P2–P3–P4	105.01(11)
P4–P5–P1	98.07(10)
P2–P1–C12	122.20(13)
P2–P1–C11	111.3(2)
P5–P1–C11	108.2(2)
**Part 2**	
P1–P2A–P3A	94.7(2)
P2A–P3A–P4A	104.1(2)
P4A–P5A–P1	95.45(16)
P2A–P1–C11A	100.4(4)
C12–P1–C11A	113.3(4)

Atom-Atom-Atom	Angle [°]
**Part 1**	
P3–P4–P5	103.97(9)
P5–P1–P2	97.79(10)
P5–P1–C12	113.76(12)
C12–P1–C11	103.2(2)
P1–C12–Si1	116.76(18)
**Part 2**	
P3A–P4A–P5A	104.85(16)
P5A–P1–P2A	94.80(17)
P5A–P1–C12	125.48(16)
P2A–P1–C12	116.50(18)
P1–C12–Si1A	129.8(2)
P5A–P1–C11A	102.3(4)

[Cp*Fe(η^4-P_5Me_2)] (**3c**)

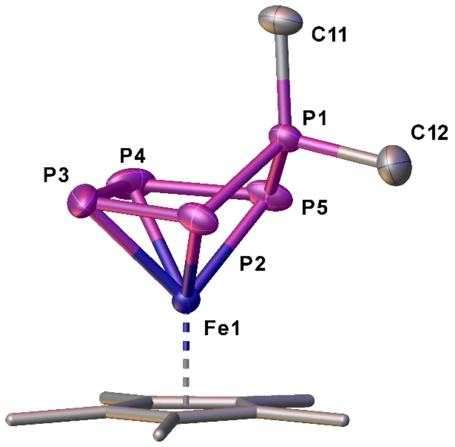

Supplementary Figure 47.

Molecular structure of **3c** with thermal ellipsoids at 50% probability level. The hydrogen atoms are omitted for clarity. The Cp* ligands are drawn in the wire frame model.

Supplementary Table 24.

Selected bond length of **3c**.

Atom-Atom	Length [Å]	Atom-Atom	Length [Å]
P1-P2	2.1398(12)	P2-P3	2.1420(16)
P1-P5	2.1336(12)	P5-P4	2.1370(17)
P1-C11	1.818(3)	P3-P4	2.1472(19)
P1-C12	1.816(4)		

Supplementary Table 25.

Selected angles of **3c**.

Atom-Atom-Atom	Angle [°]	Atom-Atom-Atom	Angle [°]
P5-P1-P2	98.20(5)	P1-P2-P3	99.53(6)
C11-P1-P2	120.43(13)	P1-P5-P4	98.69(6)
C11-P1-P5	119.27(13)	P2-P3-P4	104.10(6)
C12-P1-P2	106.54(15)	P5-P4-P3	105.21(6)
C12-P1-P5	107.20(15)	C12-P1-C11	104.21(19)

[Cp*Fe(η^4-P_5Me^{i}Pr)] (**3d**)

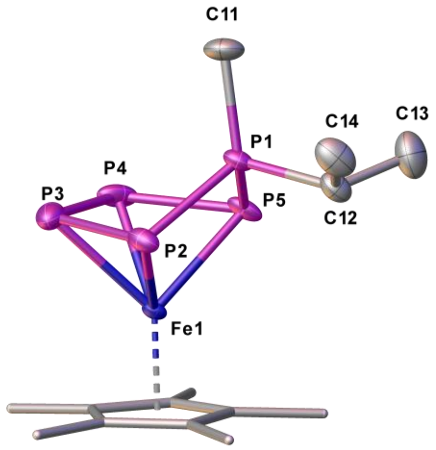

Supplementary Figure 48.

Molecular structure of **3d** with thermal ellipsoids at 50% probability level. The hydrogen atoms are omitted for clarity. The Cp* ligand is drawn in the wire frame model.

Supplementary Table 26.

Selected bond length of **3c**.

Atom-Atom	Length [Å]	Atom-Atom	Length [Å]
P5-P1	2.1490(19)	P1-C11	1.811(6)
P5-P4	2.143(2)	P2-P3	2.137(2)
P1-P2	2.138(2)	P4-P3	2.158(3)
P1-C12	1.840(6)		

Supplementary Table 27.

Selected angles of **3d**.

Atom-Atom-Atom	Angle [°]	Atom-Atom-Atom	Angle [°]
P4-P5-P1	98.96(9)	C11-P1-P2	117.7(2)
P2-P1-P5	97.86(8)	C11-P1-C12	105.8(3)
C12-P1-P5	107.3(2)	P3-P2-P1	98.88(9)
C12-P1-P2	108.0(2)	P5-P4-P3	104.31(8)
C11-P1-P5	119.5(2)	P2-P3-P4	104.75(9)

[Cp*Fe(η^4-$P_5{}^t$BuMe)] (**3e**)

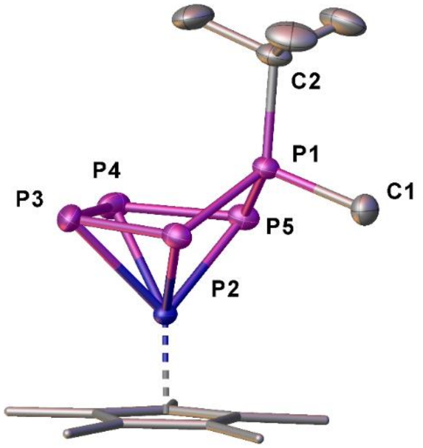

Supplementary Figure 49.

Molecular structure of **3e** with thermal ellipsoids at 50% probability level. The hydrogen atoms are omitted for clarity. The Cp* ligand is drawn in the wire frame model.

Supplementary Table 28.

Selected bond length of **3e**.

Atom-Atom	Length [Å]	Atom-Atom	Length [Å]
P1–P2	2.1462(8)	Fe1–P2	2.3301(6)
P2–P3	2.1360(1)	Fe1–P3	2.3390(8)
P3–P4	2.1476(16)	Fe1–P4	2.3390(8)
P4–P5	2.1360(1)	Fe1–P5	2.3301(6)
P1–P5	2.1462(8)	P1–C2	1.863(4)
P1–C1	1.828(4)		

Supplementary Table 29.

Selected angles of **3e**.

Atom-Atom-Atom	Angle [°]	Atom-Atom-Atom	Angle [°]
P1–P2–P3	101.40(4)	P3–P4–P5	105.20(3)
P2–P3–P4	105.20(3)	P5–P1–P2	99.15(5)
P4–P5–P1	101.40(4)	P5–P1–C1	105.20(9)
P2–P1–C1	105.20(9)	P2–P1–C2	119.84(7)
C1-P1-C2	106.04(19)	P5–P1–C2	119.84(7)

[Cp*Fe(η^4-P_5PhMe)] (**3f**)

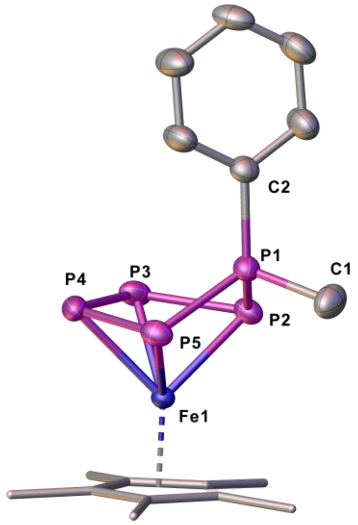

Supplementary Figure 50.

Molecular structure of **3f** with thermal ellipsoids at 50% probability level. The hydrogen atoms are omitted for clarity. The Cp* ligand is drawn in the wire frame model.

Supplementary Table 30.

Selected bond length of **3f**.

Atom-Atom	Length [Å]	Atom-Atom	Length [Å]
P1–P2	2.1498(10)	P4–P5	2.1420(11)
P2–P3	2.1433(11)	P1–P5	2.1444(10)
P3–P4	2.1454(11)	P1–C1	1.812(3)
P1–C2	1.819(3)		

Supplementary Table 31.

Selected angles of **3f**.

Atom-Atom-Atom	Angle [°]	Atom-Atom-Atom	Angle [°]
P1–P2–P3	99.65(4)	P3–P4–P5	104.89(4)
P2–P3–P4	104.87(4)	P5–P1–P2	98.20(4)
P4–P5–P1	99.84(4)	P5–P1–C1	107.55(13)
P2–P1–C1	106.27(12)	P2–P1–C2	120.76(10)
C1-P1-C2	105.52(14)	P5–P1–C2	117.59(10)

SPMe^{i}PrBnz (**4a’**)

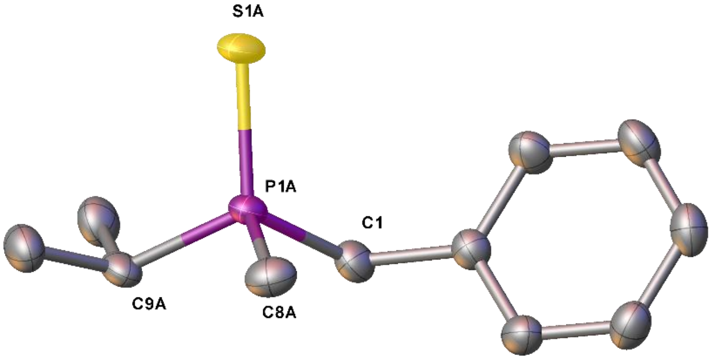

Supplementary Figure 51.

Molecular structure of **4a’** with thermal ellipsoids at 50% probability level. The hydrogen atoms are omitted for clarity.

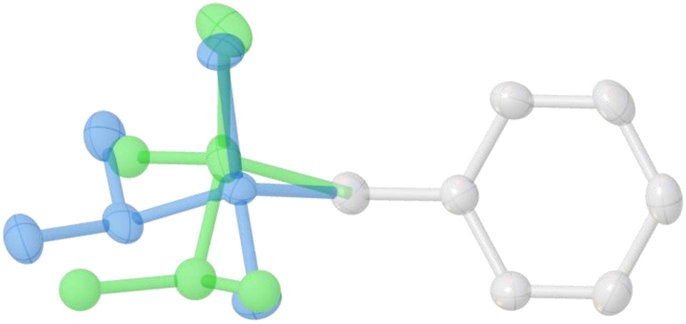

Supplementary Figure 52.

Molecular structure of **4a’** with thermal ellipsoids at 50 % probability level. The disorder is highlighted blue (Part 1) and green (Part 2) (grey = Part 0). The hydrogen atoms are omitted for clarity.

Supplementary Table 32.

Selected bond length of **4a’**.

Atom-Atom	Length [Å]
**Part 1**	
$P1_A$–$S1_A$	1.9615(15)
$P1_A$–C1	1.833(3)
**Part 2**	
$P1_B$–$S1_B$	1.91(2)
$P1_A$– $C8_B$	1.84(7)

Atom-Atom	Length [Å]
**Part 1**	
$P1_A$–$C9_A$	1.830(3)
$P1_A$–$C8_A$	1.797(3)
**Part 2**	
$P1_A$– $C9_B$	1.84(4)

Supplementary Table 33.
Selected angles of **4a'**.

Atom-Atom-Atom	Angle [°]
**Part 1**	
$C1–P1_A–S1_A$	114.79(10)
$C9_A–P1_A–S1_A$	112.86(11)
$C9_A–P1_A–C1$	103.15(12)
**Part 2**	
$C1–P1_B–S1_B$	112.6(9)
$C9_B–P1_B–S1_B$	113.8(14)
$C9_B–P1_B–C1$	98.7(14)

Atom-Atom-Atom	Angle [°]
**Part 1**	
$C8_A–P1_A–S1_A$	112.80(12)
$C8_A–P1_A–C1$	106.32(14)
$C8_A–P1_A–C9_A$	106.05(15)
**Part 2**	
$C8_B–P1_B–S1_B$	113(2)
$C8_B–P1_B–C1$	110(2)
$C8_B–P1_B–C9_B$	107(3)

SP^{t}BuMeBnz (**4b’**)

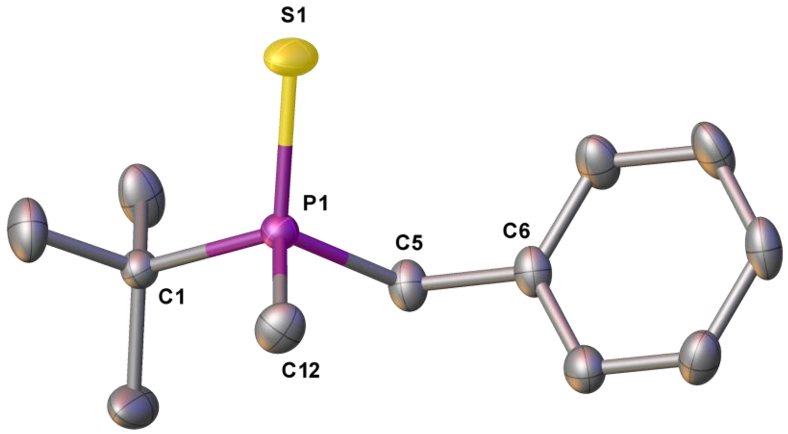

Supplementary Figure 53.

Molecular structure of **4b’** with thermal ellipsoids at 50% probability level. The hydrogen atoms are omitted for clarity.

Supplementary Table 34.

Selected bond length of **4b’**.

Atom-Atom	Length [Å]
P1–S1	1.9563(7)
P1-C1	1.855(2)
P1-C5	1.828(2)

Atom-Atom	Length [Å]
C6-C11	1.388(3)
C6-C5	1.512(3)
P1-C12	1.805(2)

Supplementary Table 35.

Selected angles of **4b’**.

Atom-Atom-Atom	Angle [°]
C1-P1-S1	113.03(7)
C5-P1-S1	113.52(7)
C5-P1-C1	105.43(9)
C12-P1-S1	111.69(8)
C12-P1-C1	107.32(10)

Atom-Atom-Atom	Angle [°]
C12-P1-C5	105.28(10)
C3-C1-P1	108.42(14)
C4-C1-P1	110.89(14)
C2-C1-P1	108.48(13)
C6-C5-P1	114.37(14)

SPPhMeBnz (**4c’**)

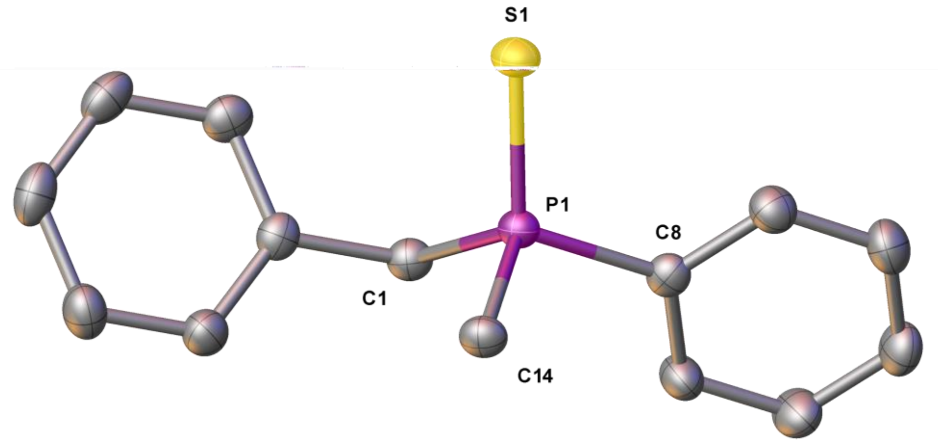

Supplementary Figure 54.

Molecular structure of **4c’** with thermal ellipsoids at 50% probability level. The hydrogen atoms are omitted for clarity.

Supplementary Table 36.

Selected bond length of **4c’**.

Atom-Atom	Length [Å]
P1–S1	1.9595(6)
P1-C8	1.8186(19)

Atom-Atom	Length [Å]
P1-C1	1.8280(19)
P1-C14	1.7987(18)

Supplementary Table 37.

Selected angles of **4c’**.

Atom-Atom-Atom	Angle [°]
C1-P1-S1	112.95(6)
C14-P1-S1	113.08(6)
C1-P1-C14	106.10(9)

Atom-Atom-Atom	Angle [°]
C1-P1-C8	112.78(6)
C8-P1-S1	105.85(9)
C14-P1-C8	105.43(9)

[K(18c6)(thf)][Cp*Fe(η^4-P_4)] (**5**)

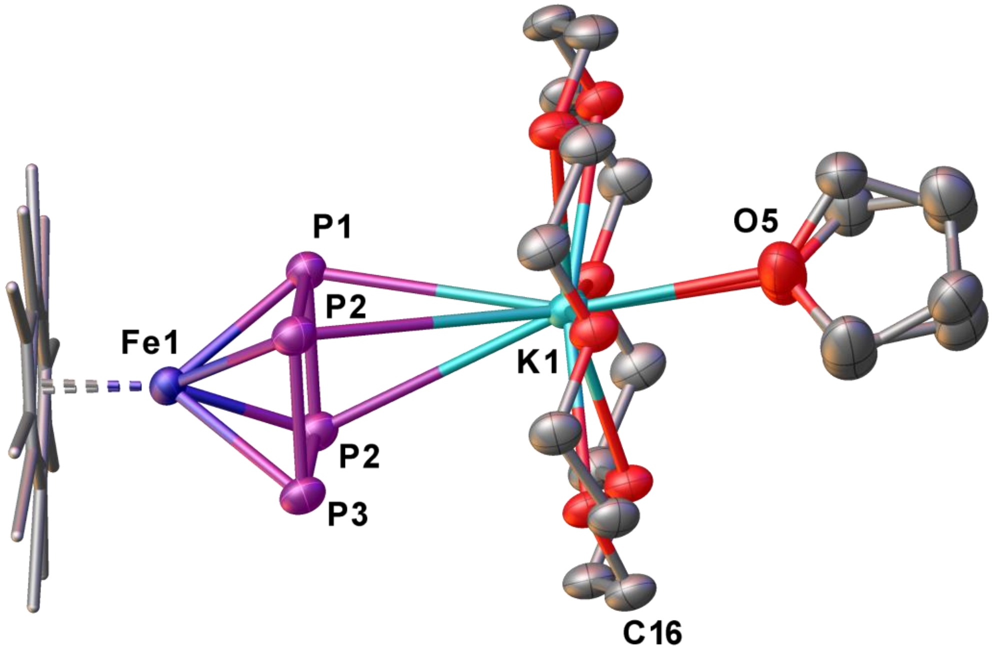

Supplementary Figure 55.
Molecular structure of **5** with thermal ellipsoids at 50% probability level. The hydrogen atoms are omitted for clarity. The Cp* ligand is drawn in the wire frame model.

Supplementary Table 38.
Selected bond length of **5**.

Atom-Atom	Length [Å]
Fe1-P1	2.3139(15)
Fe1-P3	2.3191(14)
Fe1-P2[1]	2.3129(11)
Fe1-P2	2.3130(11)
K1-P1	3.3850(16)
K1-P2[1]	3.7722(13)

Atom-Atom	Length [Å]
K1-P2	3.7722(13)
P1-P2	2.1751(13)
P1-P2[1]	2.1752(13)
P3-P2	2.1666(13)
P3-P2[1]	2.1667(13)

[1] = +x, +y, 1-z

Supplementary Table 39.
Selected angles of **5**.

Atom-Atom-Atom	Angle [°]
P2-P1-P2[1]	89.25(7)
P2-P3-P2[1]	89.69(7)

Atom-Atom-Atom	Angle [°]
P3-P2-P1	90.53(5)
P3-P2[1]-P1	90.53(5)

[1] = +x, +y, 1-z

Data availability

Reaction of **3c** with MeLi (Supplementary Figure 56) and *n*-BuLi (Supplementary Figure 57):

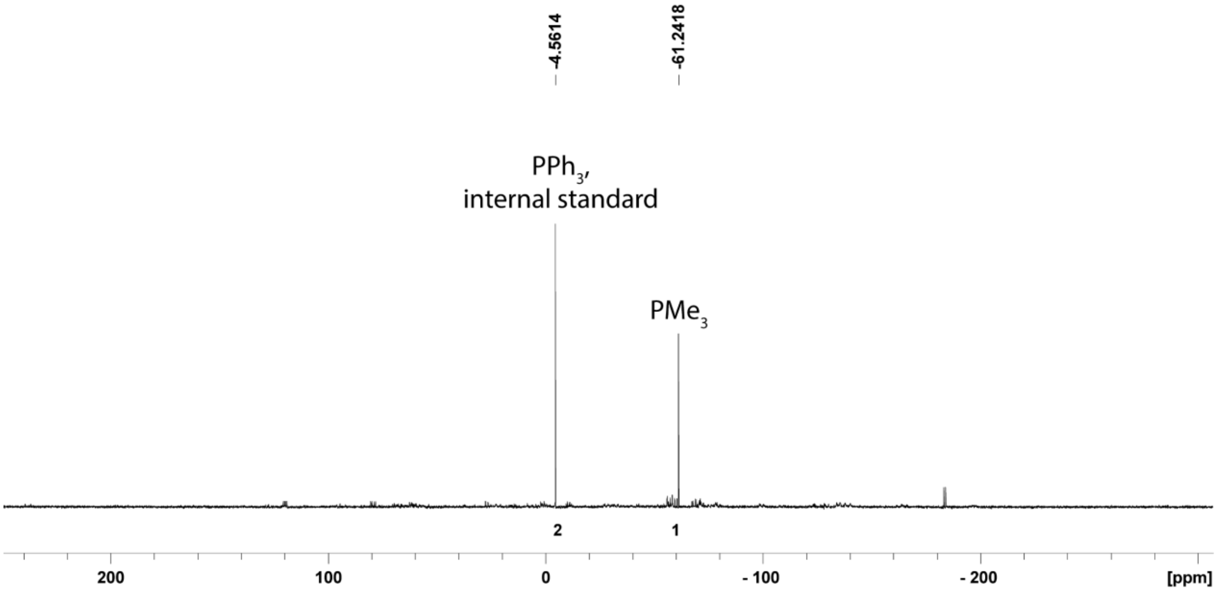

Supplementary Figure 56.

Experimental $^{31}P\{^{1}H\}$ NMR (161.98 MHz, DME|tol-d_8 capillary with PPh_3 as internal standard; c = 0.2 mol/L) spectrum of the reaction of **3c** with MeLi after five hours[1].

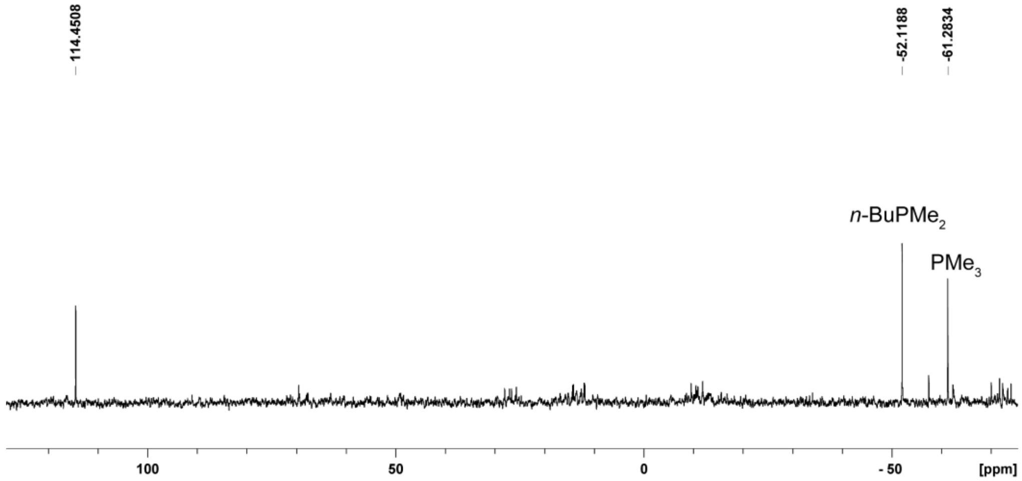

Supplementary Figure 57.

Experimental $^{31}P\{^{1}H\}$ NMR (161.98 MHz, DME|C_6D_6 capillary) spectrum of the reaction of **3c** with *n*-BuLi after one hour[2].

Reaction of **3e** with KBnz:

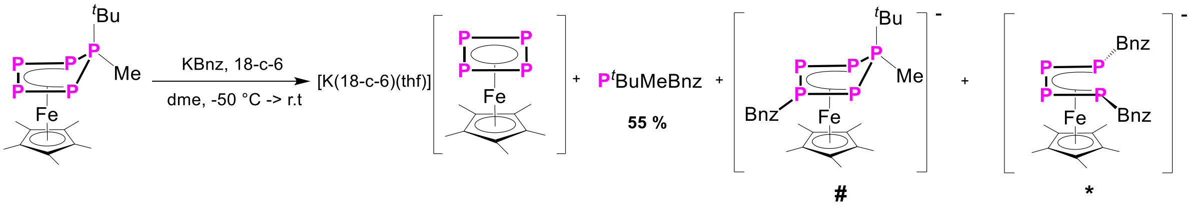

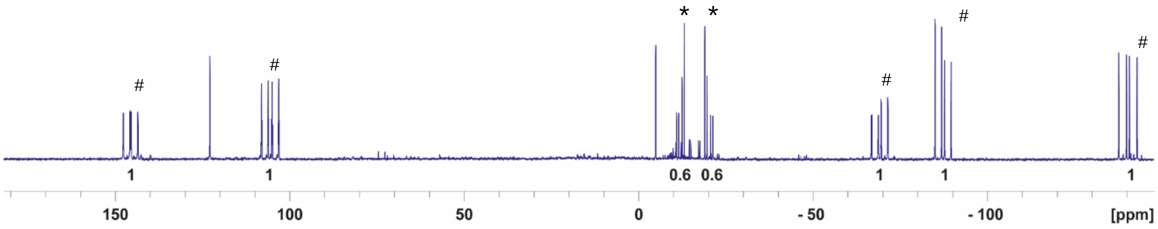

Supplementary Figure 58.

$^{31}P\{^1H\}$ NMR (161.98 MHz, THF-d8, 298 K) spectrum of the reaction of **3e** with 1.5 eq of KBnz/18c6 and after (partial) extraction of the phosphine.

Excerpt:

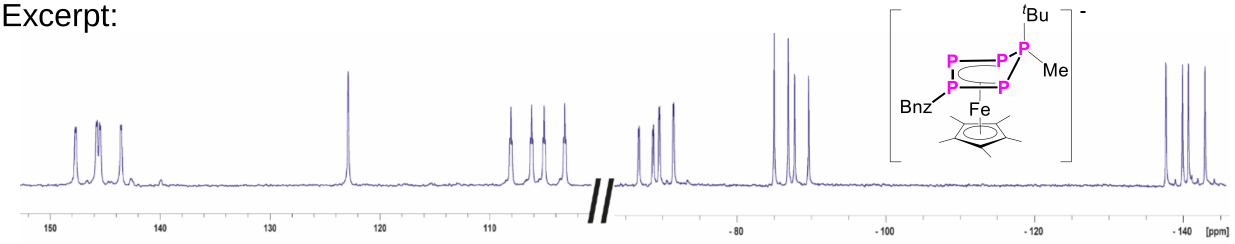

Supplementary Figure 59.

Cutout of $^{31}P\{^1H\}$ NMR (161.98 MHz, THF-d8, 298 K) spectrum of the reaction of **3e** (Supplementary Figure 59) with 1.5 eq of KBnz/18c6 and after (partial) extraction of the phosphine and postulated side products.

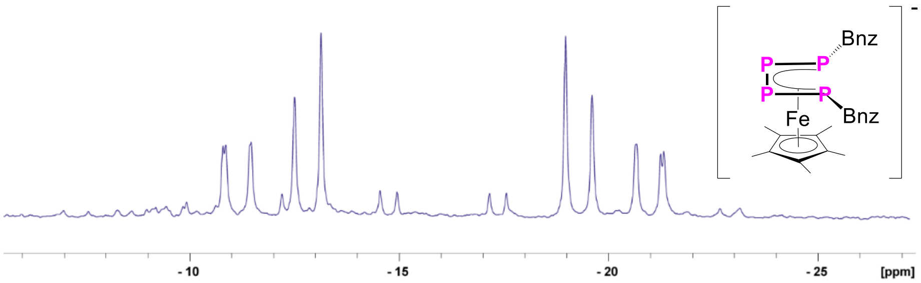

Supplementary Figure 60.

Section of $^{31}P\{^1H\}$ NMR (161.98 MHz, THF-d8, 298 K) spectrum of the reaction of **3e** (Supplementary Figure 59) with 1.5 eq of KBnz/18c6 and after (partial) extraction of the phosphine and postulated side products.

a) Reaction of **3b**, **3c**, **3d** and **3e** with; KBnz overview:

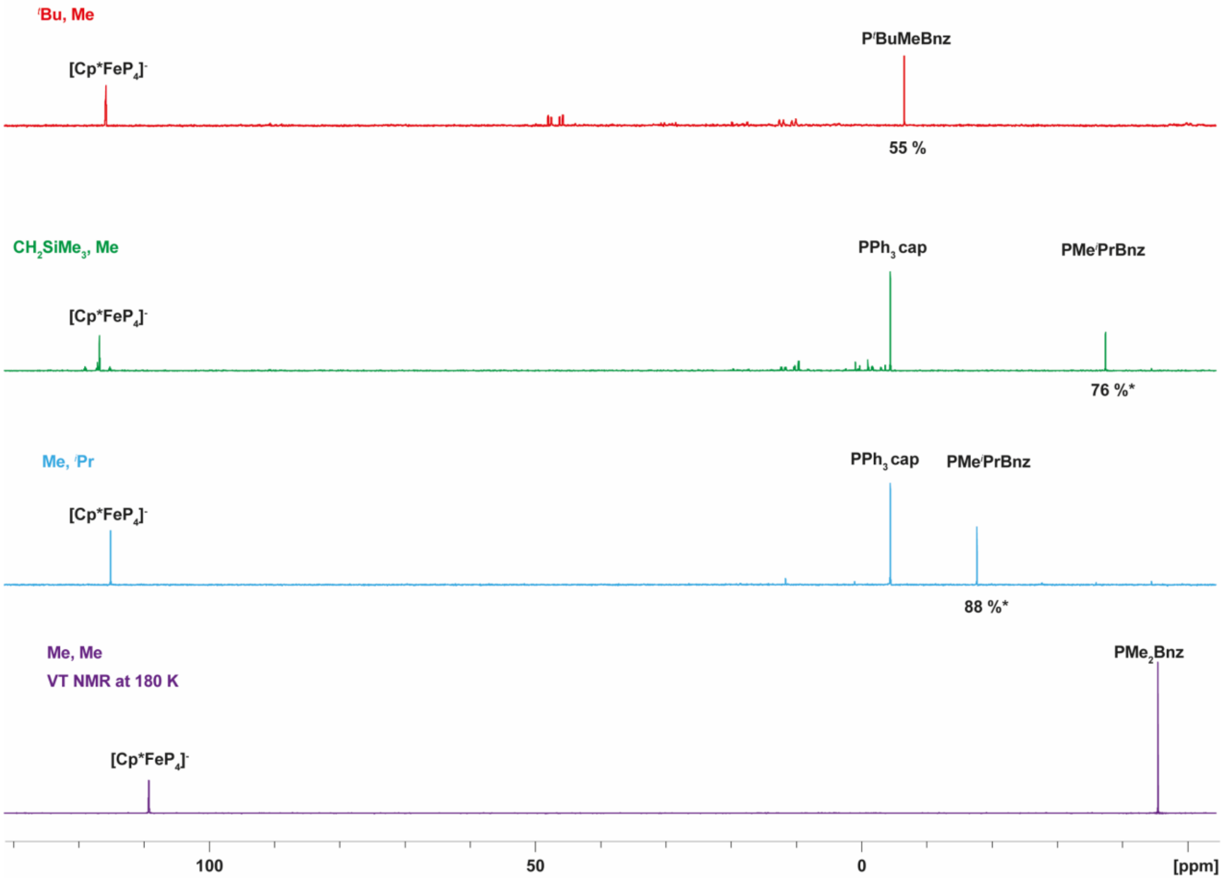

Supplementary Figure 61.

Experimental $^{31}P\{^{1}H\}$ NMR (161.98 MHz, THF-d_8) spectra of the reaction solutions of **3b, 3c, 3d** and **3e** with KBnz (* = NMR yield, according to 0.2 mol/L PPh_3 capillary as internal standard).

b) Reaction of *in situ* generated [Cp*Fe(η^4-P_5Et^{i}Pr)] (**1** with 1. EtLi, 2. 2-Iodopropane) with KBnz.

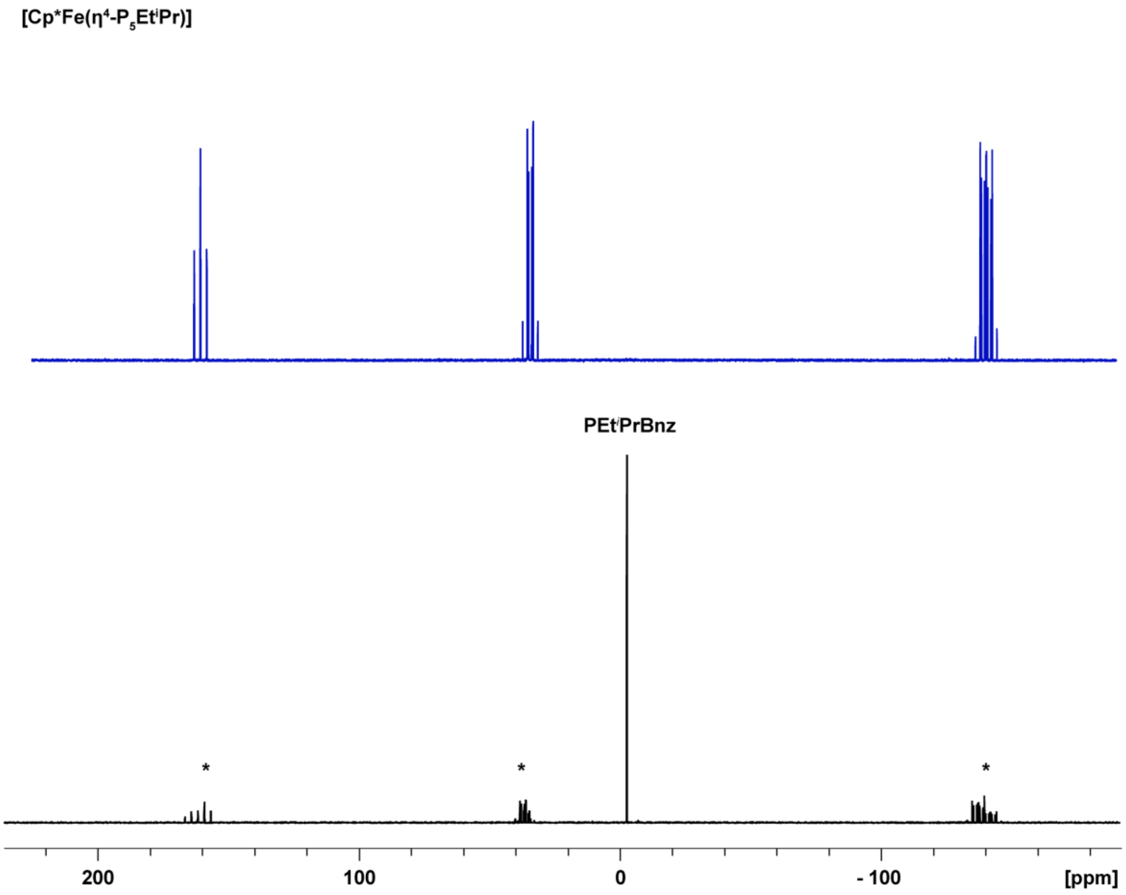

Supplementary Figure 62.
Experimental $^{31}P\{^1H\}$ NMR (161.98 MHz, C_6D_6) spectra of the reaction of **1** with 1. EtLi, 2. 2−Iodopropane (top); Reaction of [Cp*Fe(η^4-P_5Et^{i}Pr)] with KBnz (bottom).

c) Reaction of **3c** with KBnz and subsequent thermolysis with white phosphorus in tetraglyme.

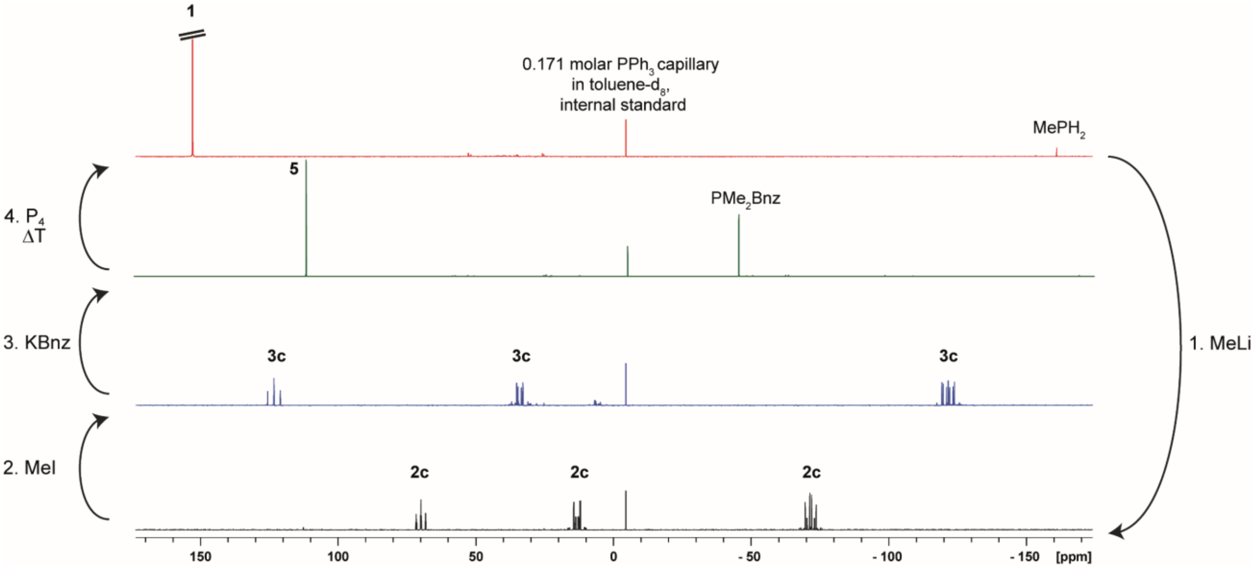

Supplementary Figure 63.
Experimental $^{31}P\{^1H\}$ NMR (161.98 MHz, tetraglyme|tol-d_8 capillary with PPh_3 as internal standard; c = 0.2 mol/L) spectrum of the one-pot reaction of **1** with 1. MeLi, 2. MeI, 3. KBnz; after thermolysis with white phosphorus: first cycle[1].

Supplementary Table 40.
Number of cycles in the reaction of **1** with 1. MeLi, 2. MeI, 3. Bnz, 4. P_4 and the corresponding yield of the distilled phosphine (PMe_2Bnz).

Cycle	Yield (PMe_2Bnz) [%]
1	87
2	82
3	67

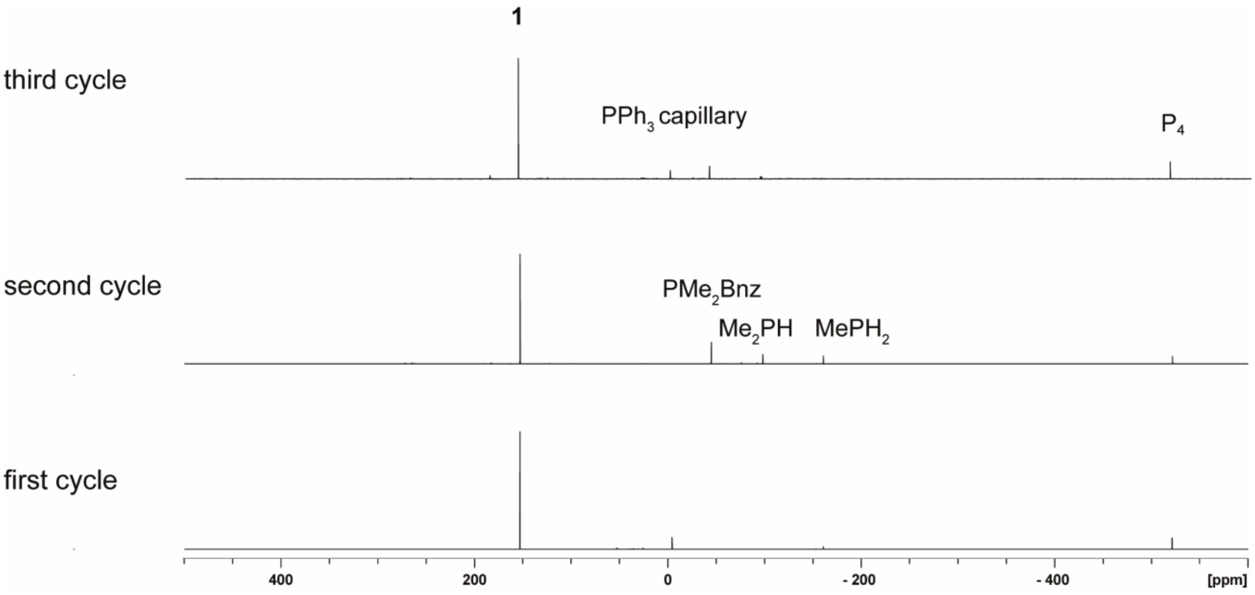

Supplementary Figure 64.

Experimental $^{31}P\{^1H\}$ NMR (161.98 MHz, tetraglyme|C_6D_6 / tol-d_8 capillary with PPh_3 as internal standard; c = 0.2 mol/L) spectrum of the reaction of **1** with 1. MeLi, 2. MeI, 3. KBnz; after thermolysis with white phosphorus.

d) Reaction of **3c** with KBnz and subsequent thermolysis with white phosphorus in triglyme.

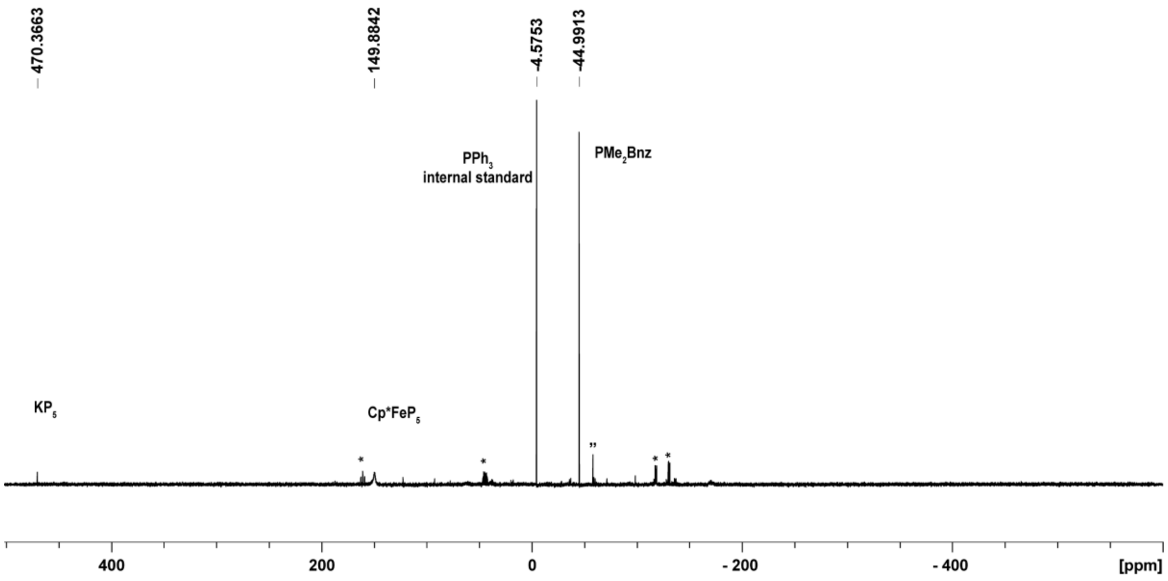

Supplementary Figure 65.
Experimental $^{31}P\{^{1}H\}$ NMR (161.98 MHz, triglyme|tol-d_8 capillary with PPh_3 as internal standard; c = 0.2 mol/L) spectrum of the reaction of **1** with 1. MeLi, 2. MeI, 3. KBnz; after thermolysis with white phosphorus (* = unidentified polyphosphides, " = PMe_3).

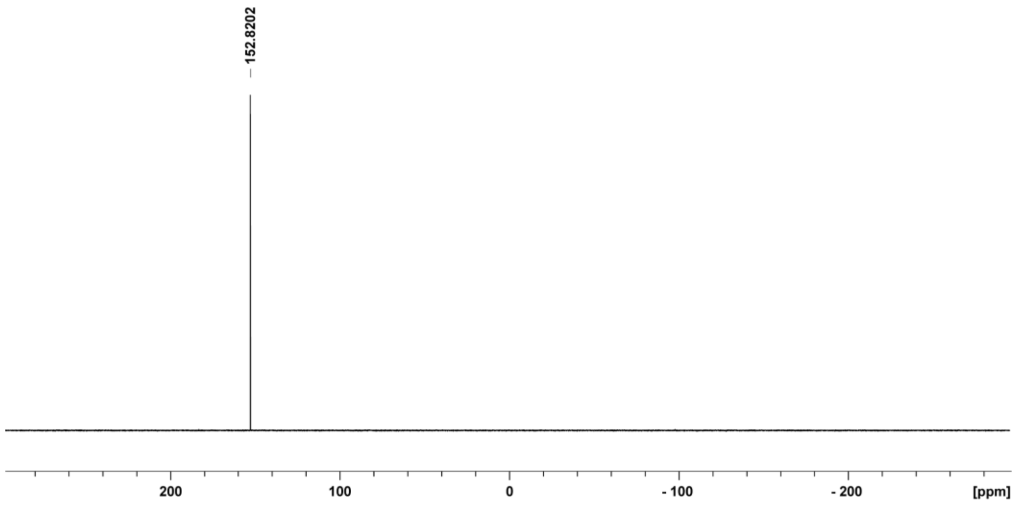

Supplementary Figure 66.
Experimental $^{31}P\{^{1}H\}$ NMR (161.98 MHz, C_6D_6) spectrum of **1**; obtained from the reaction of **1** with 1. MeLi, 2. MeI, 3. KBnz; after the thermolysis white phosphorus and chromatographic workup.

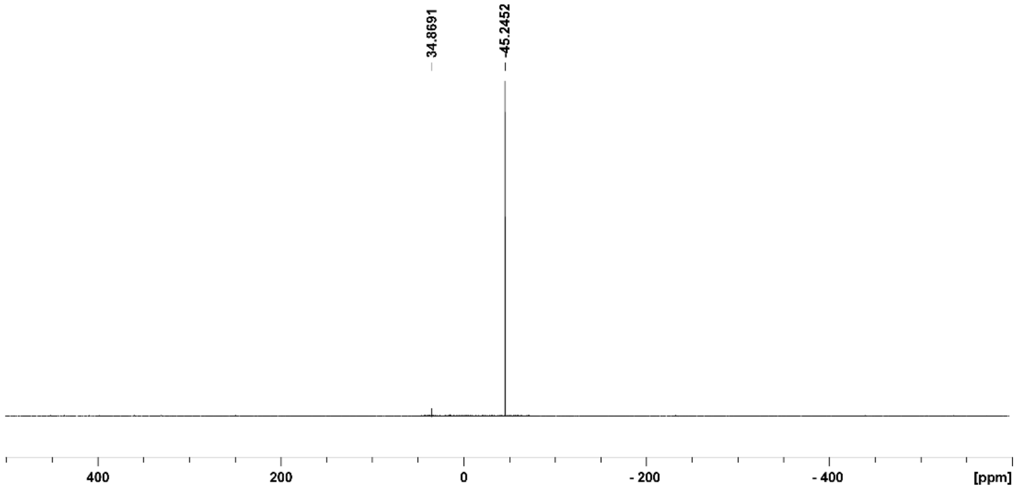

Supplementary Figure 67.

Experimental $^{31}P\{^1H\}$ NMR (161.98 MHz, C_6D_6) spectrum of the reaction of **3c** with KBnz after the thermolysis white phosphorus and distillation of PMe_2Bnz (signal at 34.9 ppm = $OPMe_2Bnz$).

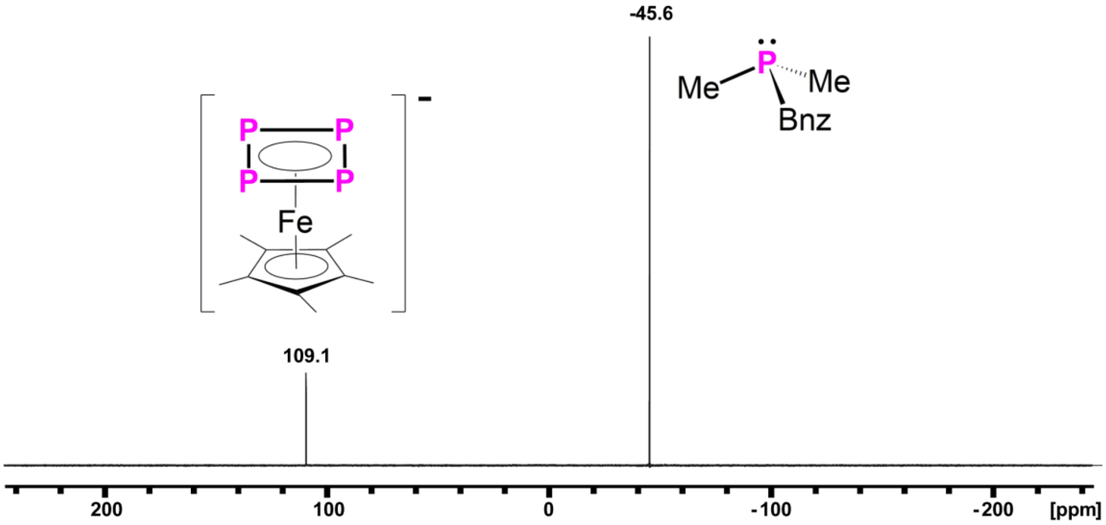

Supplementary Figure 68.

Experimental $^{31}P\{^1H\}$ NMR (242.90 MHz, THF-d_8) spectrum of the crude reaction solution (low temperature reaction) of **3c** with KBnz at 180 K.

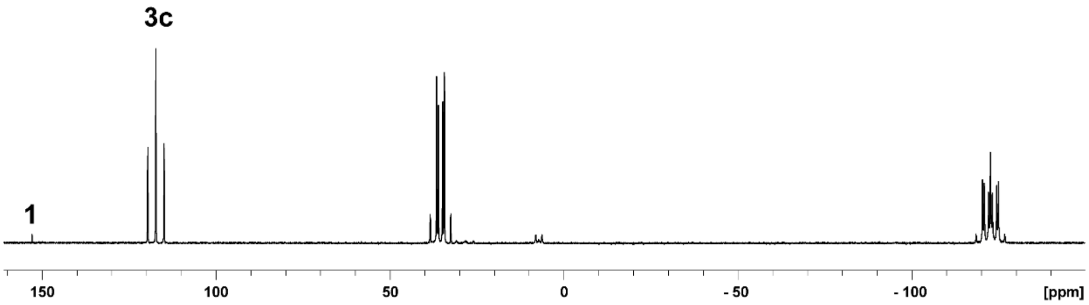

Supplementary Figure 69.

Experimental $^{31}P\{^1H\}$ NMR (161.98 MHz, THF|C_6D_6 capillary) spectrum of the reaction mixture of **1’** with 2 eq MeI.

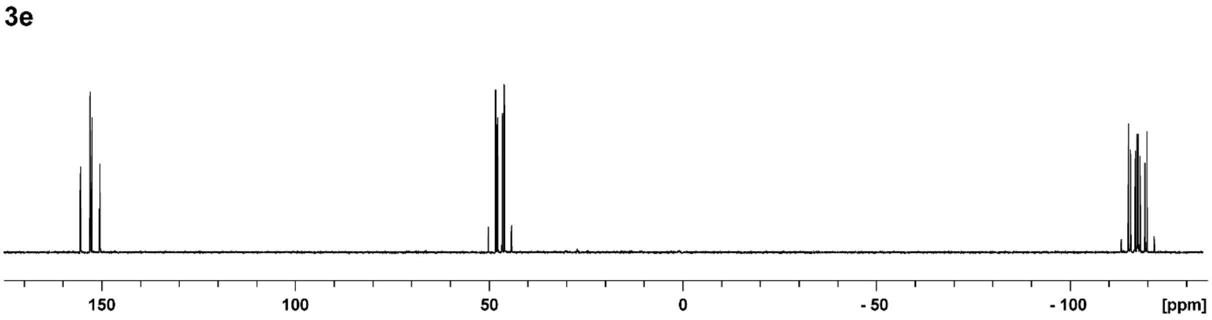

Supplementary Figure 70.

Experimental $^{31}P\{^1H\}$ NMR (161.98 MHz, THF|C_6D_6 capillary) spectrum of the reaction mixture of **1’** with 1. ^{t}BuI and 2. MeI.

Computational details

Supplementary Table 41.
Thermodynamic parameters of selected compounds calculated at D3(BJ)-B3LYP/def2TZVPP level of theory.

	Bnz^{-}	PMe_2Bnz	$[Cp^*FeP_4]^{-}$	$[Cp^*FeP_5Me_2]$	$[Cp^*FeP_5Me_2Bnz]^{-}$ (TS)
Sum of electronic and zero-point Energies [Ha]	-271.031677	-692.166444	-3019.60713	-3440.715264	-3711.755175
Sum of electronic and thermal Energies [Ha]	-271.025692	-692.155524	-3019.58813	-3440.691006	-3711.724149
Sum of electronic and thermal Enthalpies [Ha]	-271.024748	-692.15458	-3019.58719	-3440.690062	-3711.723204
Sum of electronic and thermal Free Energies [Ha]	-271.060933	-692.203688	-3019.65461	-3440.767184	-3711.816824
Zero-point vibrational energy [J/Mol]	294163.2	509535.7	602647.2	808498.3	1108593.2

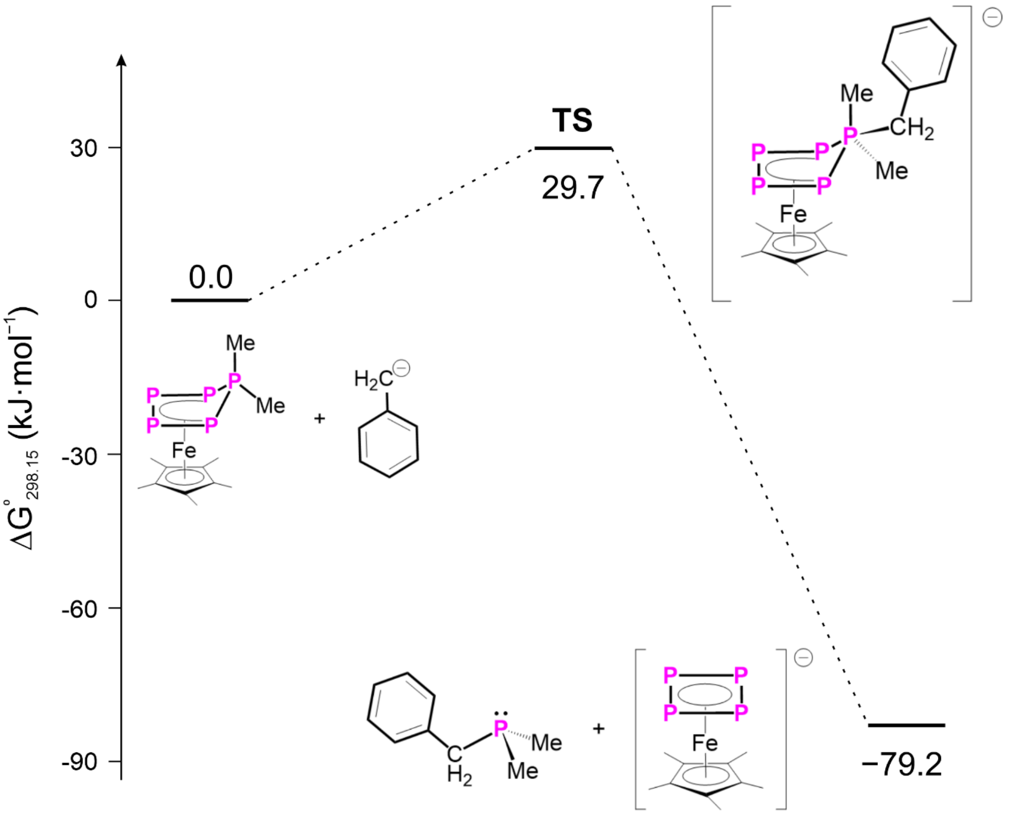

Supplementary Figure 71.
Gibbs free energy profile of the reaction of **3c** with KBnz, calculated at the B3LYP-D3(BJ)/def2-TZVPP (PCM = THF) level of theory.

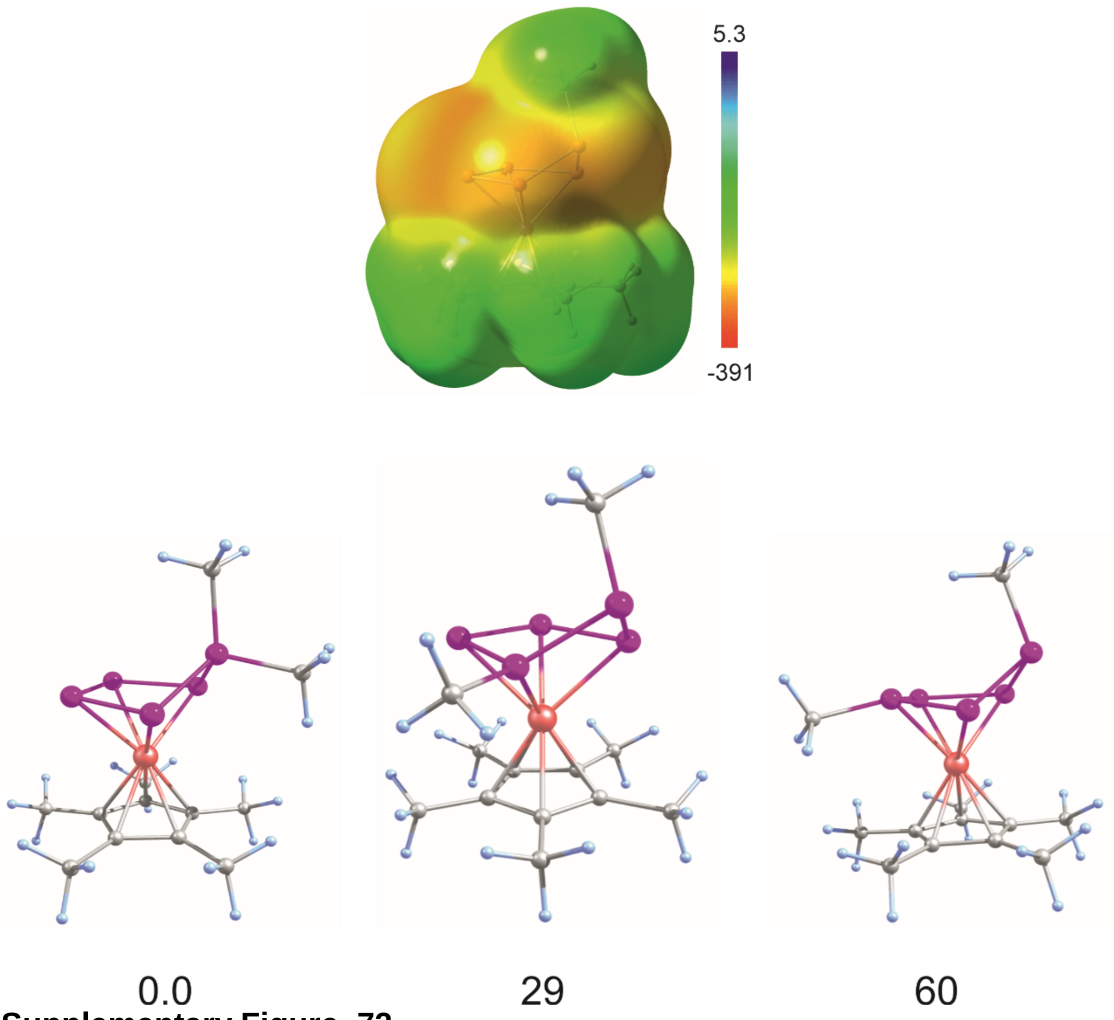

Supplementary Figure 72.
Top: Electrostatic potential mapped on electron density (isovalue = 0.001) for 2c. Colour code (blue = positive, red = negative) in kJ·mol^{-1}; Bottom: Optimised geometries of selected isomers of 3c and their relative energy in kJ·mol^{-1}. Calculated at the B3LYP-D3(BJ)/def2-TZVPP (PCM = THF) level of theory.

Supplementary Table 42. Cartesian coordinates of the optimized geometry of Bnz^{-}.

H	-1.410854000	0.228519000	2.005526000
C	-0.436567000	2.592105000	1.070860000
H	-1.045908000	2.737939000	1.953960000
H	0.004613000	3.468873000	0.613645000
C	-0.224698000	1.324357000	0.545470000
C	0.580163000	1.079798000	-0.627479000
C	0.786146000	-0.185447000	-1.137982000
C	0.222591000	-1.327070000	-0.547811000
C	-0.569729000	-1.127914000	0.593133000
C	-0.789444000	0.127552000	1.121427000
H	1.038776000	1.931081000	-1.120388000
H	1.404949000	-0.294548000	-2.023712000
H	0.388859000	-2.314764000	-0.955344000
H	-1.027092000	-1.984486000	1.079360000

Supplementary Table 43. Cartesian coordinates of the optimized geometry of PMe_2Bnz.

P	-0.030046000	-0.118633000	-0.039984000
C	1.495706000	-1.150022000	0.085844000
H	1.644871000	-1.697955000	-0.843574000
H	1.442335000	-1.861441000	0.912367000
H	2.355645000	-0.497172000	0.235636000
C	-0.176269000	0.444937000	1.713170000
H	0.622736000	1.153283000	1.932355000
H	-0.113402000	-0.387160000	2.417124000
H	-1.128185000	0.957161000	1.853669000
H	-2.328754000	-0.791640000	-2.457906000
C	-1.329246000	-1.463218000	-0.022776000
H	-1.171707000	-2.100771000	0.849271000
H	-2.289439000	-0.959515000	0.100986000
C	-1.316607000	-2.275570000	-1.284207000
C	-0.718157000	-3.535848000	-1.318208000
C	-0.671713000	-4.273816000	-2.495971000
C	-1.223228000	-3.760416000	-3.664286000
C	-1.821651000	-2.504219000	-3.643858000
C	-1.864935000	-1.769811000	-2.465551000
H	-0.287891000	-3.943062000	-0.412249000
H	-0.205819000	-5.250321000	-2.499531000
H	-1.189855000	-4.333419000	-4.580979000
H	-2.255838000	-2.096324000	-4.547043000

Supplementary Table 44. Cartesian coordinates of the optimized geometry of $[Cp^*FeP_5Me_2Bnz]^-$ (TS).

Fe	1.928627000	-0.429941000	22.755086000
P	1.730448000	-2.728201000	20.482216000
P	3.195071000	-2.267506000	22.079020000
P	3.872499000	-0.335975000	21.463288000
P	2.073043000	0.575156000	20.655060000
P	0.572066000	-0.930434000	20.923965000
C	2.659406000	-3.100257000	18.964065000
H	2.893209000	-2.156618000	18.474677000
H	2.099521000	-3.739551000	18.288444000
H	3.594275000	-3.578363000	19.261087000
C	0.984972000	-4.256100000	21.128432000
H	1.780694000	-4.955272000	21.383817000
H	0.323138000	-4.700325000	20.391831000
H	0.429254000	-4.010471000	22.032043000
C	1.616358000	-1.046959000	24.740351000
C	0.418411000	-0.485403000	24.212049000
C	0.693939000	0.864136000	23.827989000
C	2.637141000	-0.048945000	24.684466000
C	2.067507000	1.132854000	24.117634000
C	1.758795000	-2.412322000	25.328577000
H	1.087334000	-3.124462000	24.851413000
H	1.524224000	-2.399923000	26.397333000
H	2.773021000	-2.790220000	25.214980000
C	-0.913311000	-1.158834000	24.150646000
H	-1.507666000	-0.790961000	23.316369000
H	-1.481299000	-0.981049000	25.068872000
H	-0.810119000	-2.236135000	24.030445000
C	4.036762000	-0.209700000	25.180011000
H	4.408150000	-1.217454000	24.996081000
H	4.097104000	-0.026735000	26.256918000
H	4.714101000	0.484338000	24.685764000
C	2.772231000	2.432921000	23.909112000
H	3.826808000	2.277979000	23.686495000
H	2.707770000	3.063852000	24.800317000
H	2.339910000	2.986802000	23.077516000
C	-0.300073000	1.828940000	23.269623000
H	0.186443000	2.596484000	22.670379000
H	-0.855558000	2.329081000	24.068340000

H	-1.024898000	1.325205000	22.630895000
H	-1.709748000	-5.167956000	19.322824000
C	-0.476267000	-2.872026000	18.568758000
H	-1.201625000	-2.708471000	19.354232000
H	-0.129938000	-1.986058000	18.052549000
C	-0.428799000	-4.115576000	17.924254000
C	0.339864000	-4.344183000	16.737472000
C	0.459658000	-5.596651000	16.164181000
C	-0.175323000	-6.715727000	16.712696000
C	-0.952722000	-6.524407000	17.860326000
C	-1.082258000	-5.278911000	18.445459000
H	0.834284000	-3.498643000	16.273005000
H	1.056545000	-5.707356000	15.265310000
H	-0.079034000	-7.692358000	16.258689000
H	-1.472493000	-7.368323000	18.300967000

Supplementary Table 45. Cartesian coordinates of the optimized geometry of [Cp*FeP_5Me_2].

Fe	6.845963000	1.173527000	17.434821000
P	8.061706000	-0.999535000	19.212215000
P	8.985547000	0.725627000	18.329035000
P	8.653404000	0.277803000	16.249292000
P	6.794668000	-0.809458000	16.193325000
P	6.146987000	-0.931349000	18.243413000
C	8.975225000	-2.571586000	19.179665000
H	9.162981000	-2.842984000	18.143248000
H	8.380226000	-3.349309000	19.658778000
H	9.923521000	-2.456495000	19.704582000
C	7.799405000	-0.635008000	20.976046000
H	8.761937000	-0.537260000	21.475294000
H	7.233919000	-1.443482000	21.436003000
H	7.245334000	0.295716000	21.070519000
C	6.537721000	3.023668000	18.378523000
C	5.309849000	2.306859000	18.314153000
C	5.017860000	2.047538000	16.938721000
C	7.011501000	3.213349000	17.042346000
C	6.070325000	2.609833000	16.152472000
C	7.174866000	3.558163000	19.618522000
H	6.975856000	2.920092000	20.477880000
H	6.786085000	4.553838000	19.850051000
H	8.254198000	3.640160000	19.509362000
C	4.437409000	1.959767000	19.474998000
H	3.848943000	1.065832000	19.279419000
H	3.742305000	2.776752000	19.688240000
H	5.021832000	1.781678000	20.376049000
C	8.242616000	3.963375000	16.654113000
H	9.037820000	3.827821000	17.386240000
H	8.039366000	5.035334000	16.583746000
H	8.621714000	3.631680000	15.689470000
C	6.147133000	2.610026000	14.661710000
H	7.180072000	2.620509000	14.318835000
H	5.648055000	3.490397000	14.248207000
H	5.668816000	1.727515000	14.240791000
C	3.795618000	1.362001000	16.423629000
H	3.972374000	0.916364000	15.446753000
H	2.966046000	2.067360000	16.325274000
H	3.472679000	0.567809000	17.095765000

Supplementary Table 46. Cartesian coordinates of the optimized geometry of [Cp*FeP_4]$^{-}$.

Fe	0.224383000	-0.012924000	0.168183000
P	2.242601000	0.829041000	-0.669842000
P	1.499327000	-1.037371000	-1.508980000
P	0.403023000	1.828806000	-1.266914000
P	-0.341354000	-0.038103000	-2.101826000

C	-1.548383000	-0.025731000	1.208389000
C	-0.591703000	0.779427000	1.903426000
C	0.525781000	-0.046435000	2.231938000
C	0.264796000	-1.362031000	1.742096000
C	-1.017614000	-1.352345000	1.108033000
C	-1.702823000	-2.530878000	0.496479000
H	-0.982779000	-3.233393000	0.078552000
H	-2.302508000	-3.071848000	1.235503000
H	-2.366939000	-2.224813000	-0.310183000
C	1.150866000	-2.551956000	1.919944000
H	2.202964000	-2.269948000	1.906224000
H	0.954900000	-3.050636000	2.874728000
H	0.998128000	-3.281537000	1.126767000
C	1.729360000	0.375118000	3.010866000
H	1.957394000	1.427937000	2.852616000
H	1.573804000	0.226674000	4.084472000
H	2.610282000	-0.196024000	2.722245000
C	-0.755616000	2.217255000	2.276200000
H	-1.391701000	2.740656000	1.564601000
H	-1.208128000	2.318963000	3.267951000
H	0.203123000	2.733975000	2.297248000
C	-2.888302000	0.424881000	0.724462000
H	-3.207237000	-0.148779000	-0.144319000
H	-3.651656000	0.307109000	1.500312000
H	-2.872682000	1.475039000	0.435453000

Supplementary References

1. Kühl, O. *Phosphorus-31 NMR Spectroscopy*. *Phosphorus-31 NMR Spectroscopy* (Springer Berlin Heidelberg, 2009).
2. Quin, L. D., Gordon, M. D. & Lee, S. O. Effects of some phosphorus substituents on the carbon-13 chemical shifts of alkyl chains. *Org. Magn. Reson.* **6**, 503–507 (1974).
